# Metastasis-initiating cells induce and exploit a fibroblast niche to fuel malignant colonization of the lungs

**DOI:** 10.1038/s41467-020-15188-x

**Published:** 2020-03-20

**Authors:** Maren Pein, Jacob Insua-Rodríguez, Tsunaki Hongu, Angela Riedel, Jasmin Meier, Lena Wiedmann, Kristin Decker, Marieke A. G. Essers, Hans-Peter Sinn, Saskia Spaich, Marc Sütterlin, Andreas Schneeweiss, Andreas Trumpp, Thordur Oskarsson

**Affiliations:** 1 Heidelberg Institute for Stem Cell Technology and Experimental Medicine (HI-STEM gGmbH), 69120 Heidelberg, Germany; 2 Division of Stem Cells and Cancer, German Cancer Research Center (DKFZ), 69120 Heidelberg, Germany; 3 Faculty of Biosciences, University of Heidelberg, Heidelberg, Germany; 4 Division of Inflammatory Stress in Stem Cells, German Cancer Research Center (DKFZ), 69120 Heidelberg, Germany; 5 DKFZ-ZMBH Alliance, 69120 Heidelberg, Germany; 6 Institute of Pathology, University of Heidelberg, 69120 Heidelberg, Germany; 7 Department of Obstetrics and Gynaecology, University Medical Centre Mannheim, Heidelberg University, 68167 Mannheim, Germany; 8 National Center for Tumor Diseases, Heidelberg University Hospital, German Cancer Research Center, 69120 Heidelberg, Germany; 9 German Cancer Consortium (DKTK), 69120 Heidelberg, Germany

**Keywords:** Breast cancer, Cancer microenvironment

## Abstract

Metastatic colonization relies on interactions between disseminated cancer cells and the microenvironment in secondary organs. Here, we show that disseminated breast cancer cells evoke phenotypic changes in lung fibroblasts, forming a supportive metastatic niche. Colonization of the lungs confers an inflammatory phenotype in metastasis-associated fibroblasts. Specifically, IL-1α and IL-1β secreted by breast cancer cells induce CXCL9 and CXCL10 production in lung fibroblasts via NF-κB signaling, fueling the growth of lung metastases. Notably, we find that the chemokine receptor CXCR3, that binds CXCL9/10, is specifically expressed in a small subset of breast cancer cells, which exhibits tumor-initiating ability when co-transplanted with fibroblasts and has high JNK signaling that drives IL-1α/β expression. Importantly, disruption of the intercellular JNK-IL-1-CXCL9/10-CXCR3 axis reduces metastatic colonization in xenograft and syngeneic mouse models. These data mechanistically demonstrate an essential role for the molecular crosstalk between breast cancer cells and their fibroblast niche in the progression of metastasis.

## Abstract (web summary)

How cancer cells engage the microenvironment to establish metastasis is poorly understood. Here, the authors show that CXCR3-expressing breast cancer cells secrete IL-1 to induce a paracrine crosstalk with fibroblasts in the lung, which involves CXCL9/10 production and results in colonization of the lung.

## Introduction

Metastasis remains the primary threat to the lives of cancer patients with few effective therapeutic options^1^. Growing evidence indicates that metastatic colonization is not only determined by genetic and epigenetic networks in cancer cells, but also by the microenvironment^2,3^. Disseminated cancer cells that successfully colonize distant organs manage to reeducate stromal cells and generate a metastatic niche that supports secondary tumor growth^4^. Thus, the tumor microenvironment likely offers opportunities for therapeutic intervention in metastatic cancer patients^5^.

Fibroblasts form a heterogeneous group of mesenchymal cells that are commonly found within the tumor microenvironment. Cancer-associated fibroblasts (CAFs) have received considerable attention in recent years as regulators of tumor development^6^. In breast cancer, CAFs provide a cytokine and extracellular matrix (ECM) milieu that promotes growth and progression of primary tumors^7–10^. However, the precise molecular function of stromal fibroblasts at metastatic sites and their effect on metastatic progression is poorly understood. This is particularly relevant when considering the dynamic stromal changes that occur during reprogramming of the microenvironment from early colonization to the growth of overt metastasis.

In this study, we explore the dynamic molecular interactions between disseminated breast cancer cells and fibroblasts during different stages of lung metastasis. We identify a paracrine interaction between metastatic breast cancer cells and metastasis-associated fibroblasts (MAFs). We find that breast cancer cells that have invaded the lungs secrete interleukin 1 alpha/beta (IL-1α/β) to induce the chemokines CXCL9/10 in MAFs. Moreover, we demonstrate that a subset of breast cancer cells with high metastatic potential expresses the cell surface receptor CXCR3, which binds CXCL9/10. CXCR3^+^ breast cancer cells express high levels of IL-1α/β via JNK signaling, suggesting that they can both induce CXCL9/10 in MAFs and benefit from these chemokines. Importantly, using mouse models we show that these molecular interactions play a crucial role in supporting proliferation of breast cancer cells and development of metastasis. Together, our data reveal an important crosstalk between breast cancer cells and MAFs that promotes metastatic initiation and progression to overt lung metastasis.

## Results

### Metastatic breast cancer cells activate fibroblasts in the lungs

**Fig. 1:**
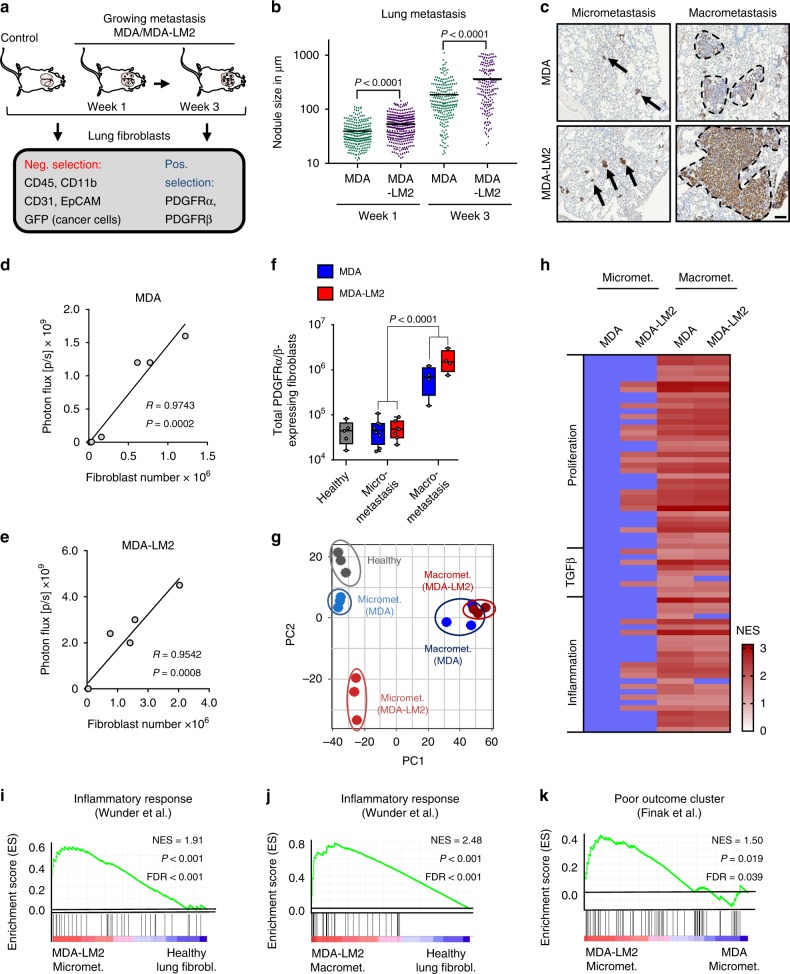
Metastatic breast cancer cells promote activation of fibroblasts during lung colonization. **a** Experimental setup for selection of fibroblasts from lungs of mice at different time points after intravenous injection of MDA-MB-231 (MDA) and MDA-MB-231-LM2 (MDA-LM2) breast cancer cells. **b** Size of metastatic nodules at week 1 or week 3 after intravenous injection of indicated breast cancer cells. Week 1, *n* = 257 (MDA) and *n* = 330 (MDA-LM2) metastatic nodules from six mice per group. Week 3, *n* = 236 (MDA) and *n* = 171 (MDA-LM2) nodules from six mice per group. **c** Representative examples of MDA and MDA-LM2 micrometastases (week 1) and macrometastases (week 3) expressing human vimentin as cancer cell-marker. Scale bar, 100 µm. Arrows point to micrometastases and dashed lines indicate margins of macrometastases. Correlation of total numbers of lung fibroblasts with lung bioluminescence from MDA (**d**) and MDA-LM2 (**e**) metastases. **f** Total number of fibroblasts from healthy and metastatic (MDA and MDA-LM2) lungs at micrometastatic and macrometastatic stages. Healthy fibroblasts, *n* = 5 mice; fibroblasts from micrometastasis MDA, *n* = 8 mice and MDA-LM2, *n* = 7 mice; fibroblasts from macrometastasis (MDA or MDA-LM2), *n* = 4 mice each group. Boxes depict median with upper and lower quartiles. Whiskers indicate minimum and maximum values and data points show biological replicates. *P* value was determined by unpaired two-tailed *t* test. **g** Principal component (PC) analysis of transcriptome of fibroblasts from metastatic or healthy lungs. **h** Overview of GSEA using numerous gene signatures representing proliferation, TGFβ- and inflammatory signaling. Heatmap shows normalized enrichment scores (NES) for signatures that were significantly changed, FDR < 0.1. Changes that are not significant when compared with healthy lung fibroblasts are indicated by blue color. Gene Sets are provided in Supplementary Table 1. **i**, **j** Enrichment of an inflammatory response signature^62^ in fibroblasts from MDA-LM2 micro- or macrometastasis compared with fibroblasts from healthy lungs. **k** Enrichment of a poor outcome gene cluster^63^ in fibroblasts isolated from MDA-LM2 compared with MDA micrometastases. **i**–**k** NES normalized enrichment score, FDR false discovery rate. *P* values were determined by random permutation tests.

To investigate evolution of fibroblasts during metastatic colonization, we established experimental lung metastases by injecting MDA-MB-231 (MDA) human breast cancer cells or the highly metastatic derivative MDA-MB-231-LM2 (MDA-LM2) cells^11^ intravenously into immunocompromised mice. At 1 week post injection (when lungs harbor primarily micrometastases) and at 3 weeks post injection (when macrometastases are prominent and widespread), we isolated fibroblasts using fluorescence-activated cell sorting (FACS) (Fig. 1a–c). Considering the different capacity of MDA and MDA-LM2 cells to grow metastasis in the lungs, the experimental approach was designed to address the qualitative difference between MDA and MDA-LM2 associated fibroblasts in metastasis at each time point. Therefore, we selected individual mice with comparable MDA or MDA-LM2 metastatic loads for our analysis based on in vivo bioluminescence imaging (Supplementary Fig. 1a). Lung fibroblasts were isolated by FACS using two positive selection markers, platelet-derived growth factor receptor (PDGFR)α and PDGFRβ, and a panel of negative selection markers (Fig. 1a and Supplementary Fig. 1b–d). Purity of sorted fibroblasts was confirmed (Supplementary Fig. 2a, b). Fibroblasts isolated from lungs with growing metastases were compared with fibroblasts from lungs of healthy, age-matched mice. We observed a striking correlation between the number of fibroblasts and metastatic burden (Fig. 1d, e). Lungs harboring macrometastases exhibited a substantial increase in the number of fibroblasts compared with lungs with micrometastases or healthy lungs (Fig. 1f). These data suggested that the fibroblast population in lung stroma expands extensively during metastatic colonization by breast cancer cells.

To determine whether stromal lung fibroblasts phenotypically evolve as lung metastases progress, we performed transcriptomic analysis of purified fibroblasts. Principal component analysis (PCA) showed that biological replicates from each group cluster together (Fig. 1g). Interestingly, fibroblasts from MDA-derived micrometastases, but not MDA-LM2-derived micrometastases, clustered close to healthy fibroblasts, whereas fibroblasts from macrometastases by both lines clustered away from healthy fibroblasts (Fig. 1g). Gene set enrichment analysis (GSEA) showed that MDA-LM2 breast cancer cells uniquely induced fibroblast activation at the micrometastatic stage, based on early signs of proliferation and inflammation as well as TGFβ-signaling (Fig. 1h and Supplementary Table 1). At the macrometastatic stage; however, proliferation and inflammation signatures were strongly induced in MAFs by both breast cancer cell lines (Fig. 1h). Inflammatory response signatures were also observed in fibroblasts from MDA-LM2-derived micrometastases and were further enriched in macrometastases (Fig. 1h–j). Gene Ontology (GO) analysis revealed similar results in that the top genes driving the PCA shift between MDA-LM2- and MDA-associated MAFs were notably involved in cell contraction, proliferation, and inflammation (Supplementary Fig. 2c). Enhanced cell contractility in MDA-LM2-associated MAFs was functionally confirmed in vitro, as lung fibroblasts demonstrated a significant increase in collagen gel contraction upon stimulation with conditioned medium (CM) from MDA-LM2 cells compared with CM from MDA cells or control medium (Supplementary Fig. 2d). Importantly, immunohistochemical staining of paraffin sections of human lung metastases from breast cancer patients revealed that 11/12 samples exhibited expression of alpha smooth muscle actin (αSMA), a marker of contractile fibroblasts (Supplementary Fig. 3a–c), indicating that reactive MAFs are also implicated in human metastases. Interestingly, fibroblasts associated with MDA-LM2 micrometastases showed a significant enrichment of genes comprising a stromal-derived “poor outcome” signature from breast cancer patients when compared with fibroblasts from lungs with MDA micrometastases (Fig. 1k). This signature was further enriched in fibroblasts isolated from lungs with MDA and MDA-LM2 macrometastases (Supplementary Table 2). These data support a model in which the phenotype of MAFs is influenced on one hand by the stage of metastatic progression and on the other by the metastatic potential of associated cancer cells. Moreover, these data indicate that transcriptomic changes in MAFs are linked to poor outcome in breast cancer patients.

### CXCL9/10 are induced in MAFs and promote lung metastasis

**Fig. 2:**
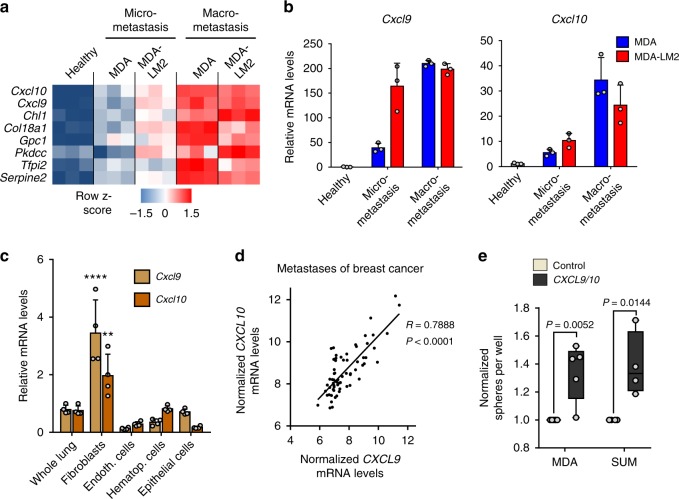
*CXCL9/10* expression is induced in metastasis-associated fibroblasts and drives oncosphere formation. **a** Heatmap showing normalized mRNA expression of genes encoding secreted proteins that are induced in fibroblasts from MDA-LM2 micrometastasis and further induced in MDA- and MDA-LM2 macrometastasis. **b** RT-qPCR validation of *Cxcl9* and *Cxcl10* induction in isolated fibroblasts from MDA- and MDA-LM2-derived lung metastases compared with expression in fibroblasts from healthy lungs. Shown are means from three mice per group with SD. **c** *Cxcl9* and *Cxcl10* expression analyzed by RT-qPCR in indicated cell types isolated from MDA-LM2-macrometastatic lungs relative to overall expression in whole lungs. Data show mean from four mice with SD. *P* values were calculated by two-way ANOVA with Tukey’s multiple comparisons test to compare *Cxcl9/10* expression in all indicated cell types. Shown are summarized *P* values of *Cxcl9/10* upregulation in fibroblasts compared with all other cell types. ** *P* < 0.01, **** *P* < 0.0001. Individual *P* values for expression of *Cxcl9/Cxcl10* in fibroblasts versus indicated cell populations are: whole lung: *P* < 0.0001/*P* = 0.0048, endothelial cells: *P* < 0.0001/*P* < 0.0001, hematopoietic cells: *P* < 0.0001/*P* = 0.0080, epithelial cells: *P* < 0.0001/*P* < 0.0001. *P* values in all other comparisons were not significant (*P* > 0.05). **d** Correlation analysis of *CXCL9* and *CXCL10* expression in dissected human metastases from breast cancer patients (GSE14020). Linear regression with Pearson correlation *r* and two-tailed *P* value, *n* = 65. **e** Oncosphere formation of MDA or SUM breast cancer cells overexpressing *CXCL9/10* or a vector control. Data represent five and four independent experiments, respectively, with quantification of ten wells per condition. Boxes depict median with upper and lower quartiles. Data points show values of biological replicates and whiskers indicate minimum and maximum values. *P* values were calculated by unpaired two-tailed *t*-tests on biological replicates.

Our findings led us to hypothesize that changes in stromal fibroblasts during metastatic colonization of the lungs may support the growth of metastasis. To address this, we aimed to identify genes expressed in MAFs that are involved in direct crosstalk with disseminated cancer cells and that are functionally relevant for metastatic growth in the lungs. Transcriptomic analysis of fibroblasts revealed that many genes encoding collagens, ECM glycoproteins, and ECM modifying enzymes were markedly induced in lungs with  macrometastases (Supplementary Fig. 4). Several of these genes, such as *Tnc, Fn1, Thbs2, Lox*, and *Serpinb2*, have been shown to promote metastatic progression^12–16^. Since early transcriptomic changes in MDA-LM2-associated fibroblasts were linked to poor outcome (Fig. 1k), we reasoned that genes upregulated early in MDA-LM2-associated fibroblasts and further induced in macrometastases would be viable prometastatic candidates. Of the 115 genes that were induced in MAFs associated with MDA-LM2 micrometastases, 50 were also induced in MAFs from lungs harboring MDA- and MDA-LM2-derived macrometastases (Supplementary Fig. 5a, b), and this group comprised a number of genes encoding proteins that are secreted or membrane-bound but exposed to the extracellular space. We prioritized these genes for further analysis and identified eight genes that represented candidates for a potential crosstalk between metastatic breast cancer cells and MAFs (Fig. 2a).

**Fig. 3:**
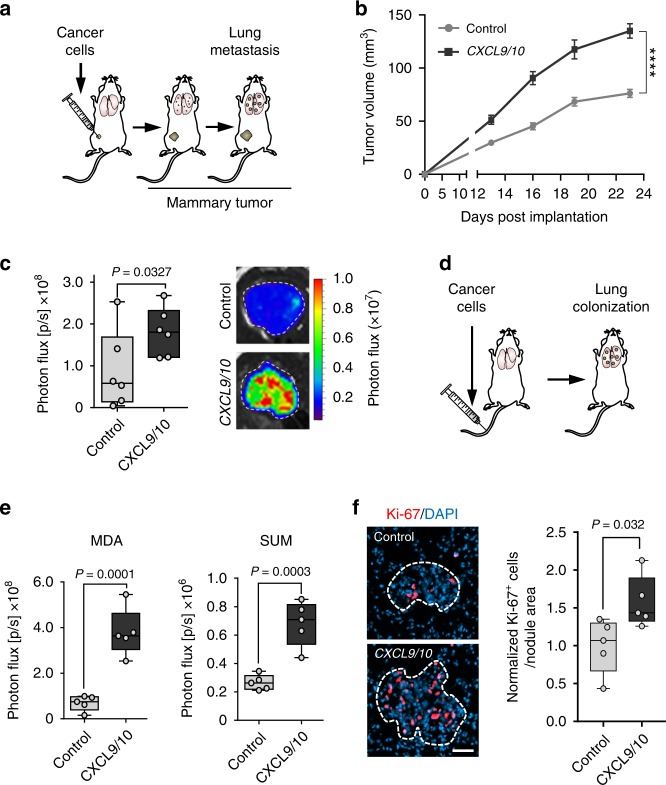
CXCL9/10 chemokines promote lung metastasis in mouse models. **a** Simplified illustration of orthotopic metastasis assay in mice, where cancer cells are implanted bilaterally into the 4th mammary fat pads. **b** Mammary tumor growth upon fat pad implantation of MDA cells overexpressing *CXCL9/10* or a vector control, *n* = 12 tumors from six mice per group. Values are mean with SEM; *t*-test was conducted for statistical analysis. **** *P* < 0.0001. **c** Ex vivo lung bioluminescence from spontaneous lung metastasis in **b**. Example ex vivo bioluminescence images are shown on the right. **d** Schematic of the lung colonization assay, where cancer cells are injected intravenously into mice. **e** Lung colonization determined by bioluminescence in mice 16 days after intravenous injection of MDA or SUM breast cancer cells overexpressing *CXCL9/10* or a vector control; *n* = 5 mice per group. **f** Immunofluorescence analysis of Ki-67 expression in MDA metastatic lung nodules from **e**. Ki-67 expressing cancer cells were quantified in metastatic foci per nodule area and normalized to the average in the control group. **c**, **e**, **f** Data points show values of biological replicates, whiskers indicate minimum and maximum values, and *P* values were calculated using unpaired two-tailed *t*-tests.

Among the earliest and most highly upregulated genes encoding secreted proteins were the chemokine (C-X-C motif) ligands 9 and 10 (*Cxcl9/10*) (Fig. 2a, b). CXCL9/10 protein levels were significantly induced in lungs harboring metastases compared with healthy lungs (Supplementary Fig. 5c, d). Expression analysis of different cell types isolated from lungs with growing metastases revealed that fibroblasts are the main source of CXCL9/10 (Fig. 2c and Supplementary Fig. 5e–h). Importantly, expression of *CXCL9* and *CXCL10* correlated markedly in dissected distant metastases samples from breast cancer patients (Fig. 2d), indicating co-expression of these cytokines in human metastasis. Therefore, to address the functional role of CXCL9/10, we ectopically expressed the two genes together in parental MDA breast cancer cells as well as in SUM159 (SUM) cells, a second human breast cancer cell line (Supplementary Fig. 6a, b). Combined overexpression of *CXCL9*/*10* in MDA and SUM parental breast cancer cells significantly increased their ability to form spheres when cultured under serum-free low adhesive conditions (Fig. 2e). These results suggested that CXCL9/10 can act on breast cancer cells with stem cell properties, as the ability to form oncospheres is associated with enrichment of stem cells in culture^17^. To analyze the role of these chemokines in metastasis, we implanted MDA breast cancer cells co-expressing *CXCL9* and *CXCL10* bilaterally into the fourth mammary fat pads of female, nonobese, diabetic-severe combined immunodeficiency gamma (NSG) mice (Fig. 3a). Notably, ectopic expression of *CXCL9/10* significantly promoted growth of mammary tumors and lung metastasis (Fig. 3b, c). Moreover, intravenous injection of *CXCL9/10*-expressing MDA or SUM cancer cells resulted in enhanced metastatic colonization as compared with the respective control cells (Fig. 3d, e and Supplementary Fig. 6c, d). We next analyzed Ki-67 expression in metastatic nodules and observed a significant increase in Ki-67^+^ cells in *CXCL9/10*-expressing nodules compared with controls (Fig. 3f). These results indicate that CXCL9/10 promote proliferation and directly fuel metastatic outgrowth in the lung. To address whether both *CXCL9* and *CXCL10* contribute to oncosphere formation and lung metastasis, we overexpressed the genes individually in MDA or SUM cancer cells (Supplementary Fig. 7a). Indeed, both *CXCL9* and *CXCL10* promoted sphere formation and lung colonization by breast cancer cells (Supplementary Fig. 7b, c). Together, these data show that CXCL9/10 represent components of the metastatic niche that are co-induced in activated MAFs in lungs and support lung colonization.

### IL-1α/β induce *CXCL9/10* expression in lung fibroblasts

**Fig. 4:**
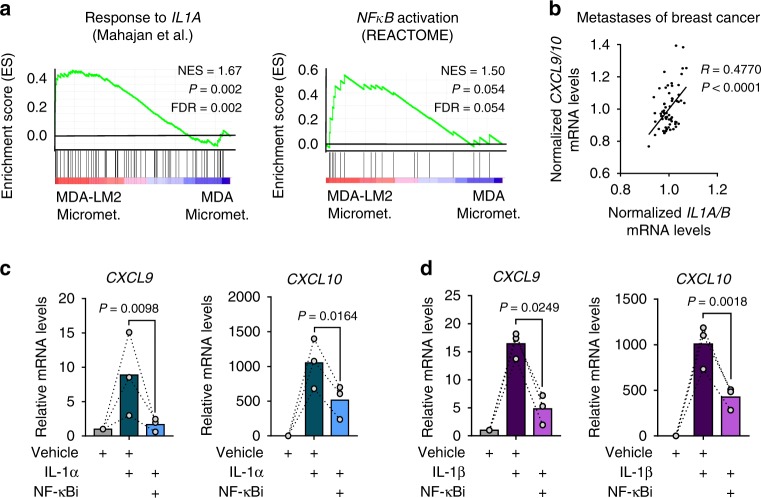
*CXCL9/10* expression in fibroblasts is induced by IL-1α/β via NF-κB signaling. **a** GSEA of IL-1α (ref. ^64^) and NF-κB (REACTOME) signatures in fibroblasts isolated from lungs bearing MDA-LM2 micrometastases compared with MDA parental micrometastases. NES normalized enrichment score, FDR false discovery rate. *P* values were determined by random permutation tests. **b** Correlation analysis of mean *CXCL9/10* and mean *IL1A/B* expression levels in data sets from human breast cancer metastases. Linear regression with Pearson correlation *r* and two-tailed *P* value. *n* = 65. *CXCL9* and *CXCL10* expression in MRC-5 human lung fibroblasts treated with 1 ng/ml recombinant human IL-1α (**c**) or IL-1β (**d**) alone or in combination with 5 µM JSH-23 (NF-κBi) for 48 h. Linked sets of values are relative expression in biological replicates and bars show mean. *P* values were determined by ratio-paired one-tailed *t*-tests; *n* = 3 independent experiments. Expression was analyzed by RT-qPCR.

We next examined how *CXCL9/10* are induced in lung fibroblasts during metastatic colonization. GSEA revealed a significant enrichment of pro-inflammatory IL-1 cytokine response and NF-κB signaling in fibroblasts from MDA- and MDA-LM2-derived macrometastases (Figs. 1h, 4a; and Supplementary Table 1). Notably, enrichment of this signaling was observed already at a micrometastatic stage in MDA-LM2-associated fibroblasts, thus analogous to the observed induction of *Cxcl9/10* (Figs. 1h, 2a, 4a; and Supplementary Table 1). In line with this, we observed a significant correlation between *CXCL9/10* and *IL1A/B* expression in dissected metastases samples from breast cancer patients (Fig. 4b). Therefore, we hypothesized that IL-1α/β present in metastatic lungs may induce *Cxcl9/10* expression in lung fibroblasts. Indeed, stimulation with recombinant IL-1α or IL-1β induced expression of *CXCL9/10* in MRC-5 human lung fibroblasts, and this induction was mediated by NF-κB activity (Fig. 4c, d). Secretion of CXCL10 by IL-1-treated fibroblasts was confirmed by ELISA on CM (Supplementary Fig. 8a). Moreover, blockade of IL-1 receptor (IL-1R) signaling through the use of an inhibitory human anti-IL-1R monoclonal antibody blunted the induction of *CXCL9/10* in fibroblasts by recombinant IL-1α or IL-1β (Supplementary Fig. 8b, c). These results indicated that IL-1α/β-mediated induction of CXCL9/10 in lung fibroblasts occurs via activation of IL-1R and downstream NF-κB signaling.

### Metastatic breast cancer cells are a direct source of IL-1

**Fig. 5:**
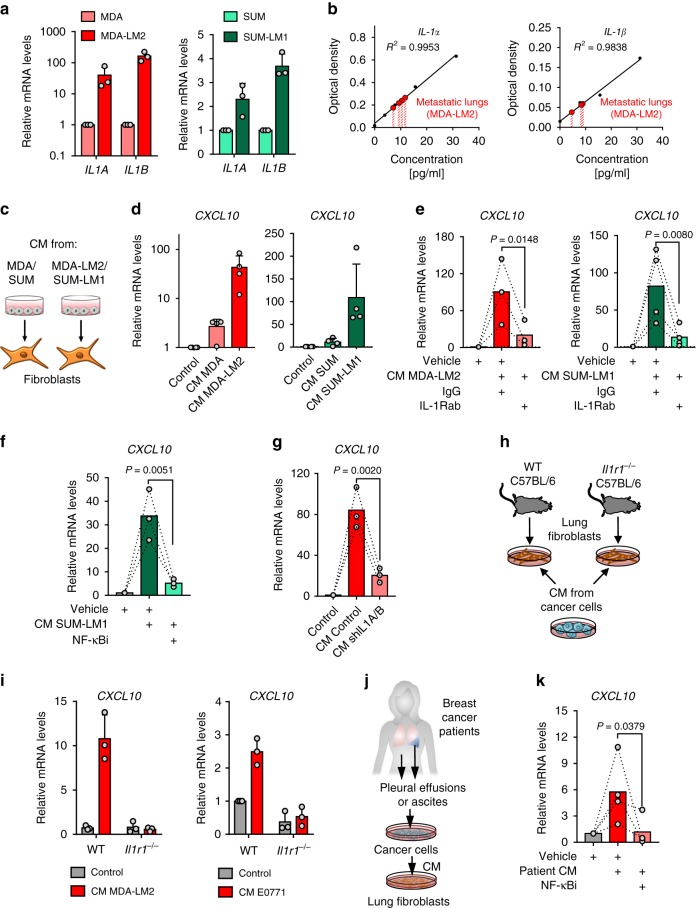
Metastatic breast cancer cells express IL-1α/β to directly induce *CXCL9/10* in reactive fibroblasts. **a** *IL1A* and *IL1B* expression in MDA and SUM parental breast cancer cells and their metastatic derivatives MDA-LM2 and SUM-LM1 analyzed by RT-qPCR; *n* = 3. **b** Human IL-1α and IL-1β protein levels in the lungs from xenograft mouse models after intravenous injection of MDA-LM2 cancer cells measured by ELISA. Shown are standard curves and interpolated IL-1α and IL-1β concentrations in lung lysates from four mice (IL-1α) and 3 mice (IL-1β). Analyzed samples were diluted 1:2. **c** Schematic of setup for experiments using cancer cell conditioned medium (CM) to stimulate fibroblasts. **d** *CXCL10* expression in MRC-5 fibroblasts treated with CM from MDA/SUM cells or MDA-LM2/SUM-LM1 for 48 h; *n* = 4 experiments. *CXCL10* expression in MRC-5 fibroblasts treated with MDA-LM2 or SUM-LM1 cancer cell CM alone or co-treated with 20 µg/ml IL-1R1 blocking antibody (IL-1Rab) or isotype control (IgG) (**e**), or 5 µM JSH-23 (NF-κBi) or vehicle (**f**) for 48 h. MDA-LM2; *n* = 3; SUM-LM1, *n* = 4 (**e**) and *n* = 3 (**f**). **g** *CXCL10* expression in fibroblasts treated with CM from control or *IL1A/B* knockdown (shIL1A/B) MDA-LM2 cancer cells for 48 h; *n* = 3 experiments. **h** Schematic of setup where fibroblasts isolated from lungs of wild type (WT) and IL-1 receptor deficient (*Il1r1*^−/−^ C57BL/6) mice were treated with CM from MDA-LM2 or E0771 cells or control medium. **i** *CXCL10* mRNA levels determined by RT-qPCR in CM-treated WT or *Il1r1*^−/−^ fibroblasts as in **h**; *n* = 3 experiments. **a**, **d**, **i** Data points depict expression in biological replicates, bars show mean with SD. **j** Schematic setup where lung fibroblasts were treated with CM from primary cancer cells derived from pleural effusions or ascites of metastatic breast cancer patients, alone or in combination with 5 µM JSH-23 (NF-κBi) for 48 h. **k** *CXCL10* mRNA levels determined by RT-qPCR in lung fibroblasts as in (**j**). CM from *n* = 4 different patient-derived cancer cell samples were used. **e**–**g**, **k** The linked set of values are relative expression in independent experiments. Bars depict mean. *P* values in were determined by ratio-paired one-tailed *t*-tests.

We considered whether breast cancer cells may be a direct source of IL-1 ligands to induce *CXCL9/10* in MAFs. Indeed, *IL1A*/*B* were expressed by MDA and SUM breast cancer cell lines, and *IL1A/B* expression levels were significantly increased in the respective lung metastatic derivatives, MDA-LM2 and SUM-LM1, suggesting an association with metastatic potential (Fig. 5a). Moreover, analyses of secreted IL-1α/β in CM confirmed a striking increase in MDA-LM2 and SUM-LM1 cells compared with parental counterparts (Supplementary Fig. 9a). Human specific ELISAs on whole lung homogenates from mice bearing MDA-LM2-derived lung metastases further confirmed expression of cancer cell-derived IL-1α/β in metastatic lungs (Fig. 5b). Since IL-1α/β are secreted cytokines, we investigated whether CM of cultured breast cancer cells drives induction of *CXCL9/10* in fibroblasts in vitro. We treated MRC-5 cells with CM from parental (MDA/SUM) or highly metastatic (MDA-LM2/SUM-LM1) breast cancer cells (Fig. 5c). In line with the observed high *IL1A/B* mRNA and secreted protein levels by metastatic breast cancer cells (Fig. 5a and Supplementary Fig. 9a), treatment with CM from MDA-LM2 or SUM-LM1 cancer cells induced a stronger upregulation of *CXCL10* in MRC-5 fibroblasts compared to CM from parental cell counterparts (Fig. 5d). Moreover, addition of a blocking antibody against IL-1R or an NF-κB inhibitor to the CM of MDA-LM2 and SUM-LM1 cells prevented induction of *CXCL10* in MRC-5 fibroblasts (Fig. 5e, f and Supplementary Fig. 9b). Although breast cancer cells express IL-1R, they did not respond to IL-1 treatment by upregulating *CXCL9/10* like fibroblasts (Supplementary Fig. 9c). Instead, IL-1 stimulation induced further expression of *IL1A*/*B* in breast cancer cells, indicating a positive autocrine feedback loop (Supplementary Fig. 9d).

Importantly, treatment of lung fibroblasts with CM from MDA-LM2 cells transduced with shRNA against *IL1A/B* or treatment of IL-1R knockout (*Il1r1*^−/−^) fibroblasts with CM from MDA-LM2 cells also prevented upregulation of *CXCL10* in the fibroblasts (Fig. 5g–i, Supplementary Fig. 9e). These experiments confirm that IL-1α/β are indeed the factors contained within CM from metastatic breast cancer cells that drive *CXCL10* expression in lung fibroblasts via IL-1R. Notably, similar effects were observed when we stimulated fibroblasts with CM from patient-derived cancer cells that were collected from pleural effusions or ascites of metastatic breast cancer patients, inducing *CXCL10* in fibroblasts in an NF-κB-dependent manner (Fig. 5j, k). These findings indicate that metastatic breast cancer cells secrete IL-1α/β, which activate IL-1R on lung fibroblasts to induce NF-κB-dependent expression of *CXCL9/10*.

### IL-1 signaling promotes tumor initiation and lung colonization

**Fig. 6:**
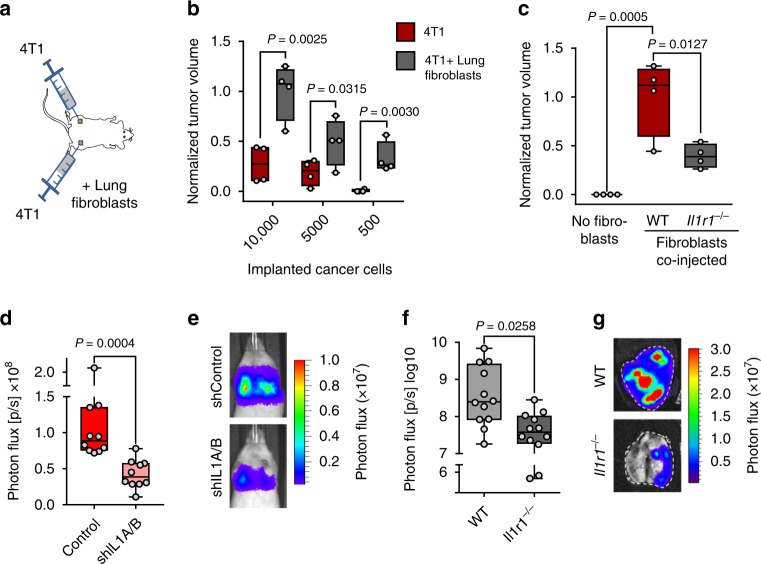
IL-1 signaling promotes tumor initiation and metastatic colonization of the lung. **a** Diagram of tumor initiation experiment where 4T1 mouse mammary tumor cells were subcutaneously implanted alone or co-implanted with primary mouse lung fibroblasts into the flanks BALB/c mice. **b** Relative tumor sizes in NSG mice after subcutaneous implantation of 4T1 cells in limiting dilutions, alone or in combination with lung fibroblasts; *n* = 4 mice per group. Tumor sizes were normalized to average tumor size established by 10,000 4T1 cells co-injected with lung fibroblasts. *P* values were calculated by unpaired one-tailed *t*-tests. **c** Relative tumor sizes in NSG mice after subcutaneous injection of 50 MDA-LM2 breast cancer cells alone or in combination with lung fibroblasts obtained from WT or *Il1r1*^−/−^ C57BL/6 mice; *n* = 4 mice per group. Tumor sizes were normalized to average tumor size established by MDA-LM2 cancer cells co-injected with WT lung fibroblasts. *P* values were calculated by ordinary one-way ANOVA with Tukey’s multiple comparisons test. **d** Lung colonization in mice injected intravenously with control or shIL1A/B*-*transduced MDA-LM2 breast cancer cells as determined by bioluminescence. *P* value was calculated by unpaired one-tailed *t*-test; *n* = 10 mice per group. **e** Representative luminescence images from each group in **d**. **f** Lung colonization in WT or age-matched *Il1r1*^−/−^ C57BL/6 mice injected intravenously with E0771 mammary cancer cells as determined by ex vivo lung bioluminescence 24 days post injection. *P* value was calculated by unpaired one-tailed *t*-test; *n* = 12 mice per group from three independent experiments. **b**–**d**, **f** Boxes depict median with upper and lower quartiles, whiskers indicate minimum and maximum values, and data points show biological replicates. **g** Representative ex vivo bioluminescence images from each group in **f**.

To address whether fibroblast interaction via IL-1/IL-1R axis plays a functional role in breast cancer progression and lung metastatic colonization, we used syngeneic and xenograft mouse models. 4T1 mouse mammary tumor cells were implanted subcutaneously into the flanks of female BALB/c mice in limiting dilutions. Cancer cells were implanted alone on one side of the mice and co-injected with lung fibroblasts from age-matched mice on the other side (Fig. 6a). Tumors from 4T1 cancer cells co-injected with lung fibroblasts grew significantly larger compared with tumors from cancer cells alone, and the population maintained increased tumor-initiating ability upon limiting dilution, indicating that fibroblasts promote cancer stem cell properties (Fig. 6b). The increase in tumor-initiating ability and growth, mediated by cancer cells co-implanted with fibroblasts, was also observed using MDA-LM2 cells; however, this induction was significantly restrained when *Il1r1*^−/−^ fibroblasts were co-implanted (Fig. 6c). This suggests that fibroblasts promote tumor initiation and growth via IL-1R. To determine whether IL-1 expressed in cancer cells is required for metastatic colonization of the lung, we injected control and shIL1A/B*-*transduced MDA-LM2 cancer cells intravenously into NSG mice and measured lung colonization. *IL1A/B* knockdown cancer cells showed significantly reduced ability to colonize the lung, indicating that IL-1 cytokines are required for the growth of lung metastasis (Fig. 6d, e). In line with this, when E0771 mouse mammary tumor cells were injected into syngeneic *Il1r1*^−/−^ mice, metastatic colonization of the lungs was also significantly reduced (Fig. 6f, g). Together, the results suggest a crucial role for IL-1R signaling in lung metastasis.

### JNK signaling drives IL-1 production in breast cancer cells

**Fig. 7:**
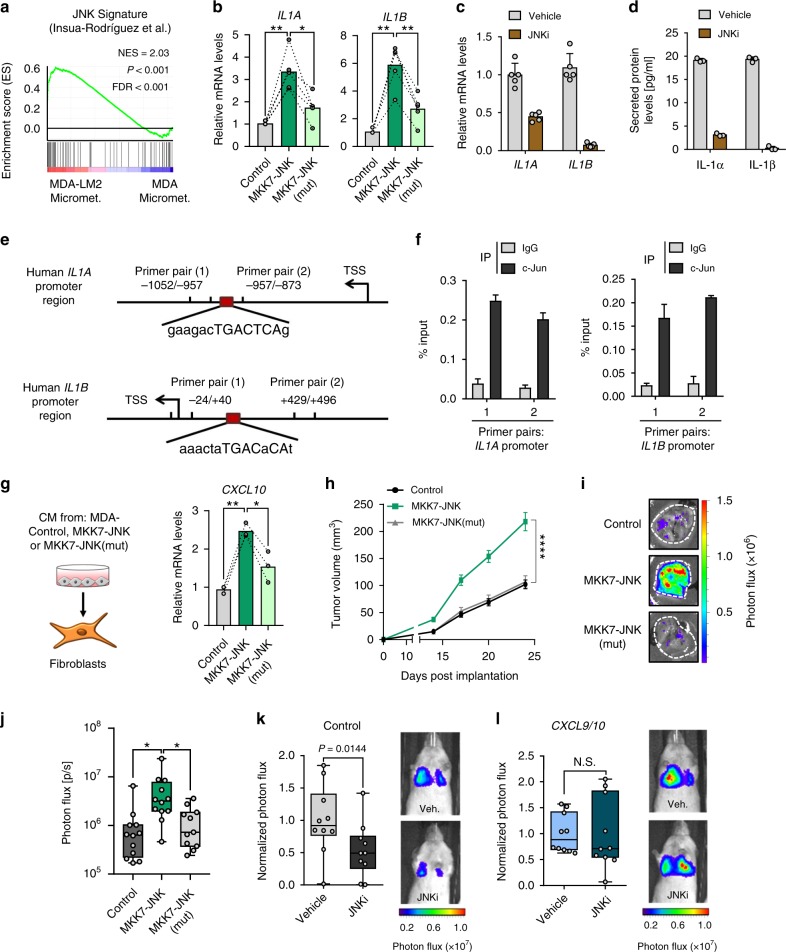
JNK signaling in breast cancer cells induces *IL1A/B*. **a** Enrichment of JNK signature^18^ in MDA-LM2 cells compared with parental MDA cells, isolated from lung micrometastases. NES normalized enrichment score, FDR false discovery rate. *P* value was determined by random permutation test. **b** *IL1A* and *IL1B* mRNA levels in MDA cells transduced with activated JNK (MKK7-JNK), a mutated version of JNK (MKK7-JNK(mut)), or vector control, determined by RT-qPCR. *n* = 5. *IL1A/B* mRNA (**c**) and protein (**d**) levels in MDA-LM2 cells or MDA-LM2 conditioned medium (CM) respectively, after treatment with 5 µM CC-401 JNK inhibitor (JNKi) or DMSO (vehicle) for 48 h. Data points depict expression in biological replicates, bars show mean with SD; *n* = 5 (mRNA), *n* = 3 (protein). **e** Maps of *IL1A* and *IL1B* promoter regions showing c-Jun consensus binding sites and primer positions for ChIP-qPCR. TSS transcription start site. **f** ChIP-qPCR analysis of *IL1A* and *IL1B* promoter chromatin bound to c-Jun in MDA-LM2 cells. Bars depict mean with SD. **g** *CXCL10* expression in MRC-5 fibroblasts treated with CM from MDA cells expressing MKK7-JNK, MKK7-JNK(mut), or control vector; *n* = 3. **b**, **g** Linked sets of values show expression in independent experiments and bars depict mean. *P* values were calculated by repeated measures one-way ANOVA with Tukey’s multiple comparisons tests. * *P* < 0.05, ** *P* < 0.01. **h** Primary tumor growth upon injection of MDA-control, MKK7-JNK, or MKK7-JNK(mut) cells into mammary fat pads of NSG mice; *n* = 24 (control, MKK7-JNK) tumors from 12 mice per group and *n* = 22 (MKK7-JNK(mut)) tumors from 11 mice, from two independent experiments. Values are mean with SEM. Multiple *t*-tests were conducted for statistical analysis. **** *P* < 0.0001. **i** Representative ex vivo lung bioluminescence images from spontaneous metastasis in **h**. **j** Quantification of ex vivo lung bioluminescence from **h**, **i**. Ordinary one-way ANOVA with Tukey’s multiple comparisons tests was conducted for statistical analysis. * *P* < 0.05. Lung colonization of control (**k**) or *CXCL9/10*-overexpressing MDA-LM2 cancer cells (**l**) pretreated with 5 µM JNKi or vehicle for 48 h, quantified by lung bioluminescence. Representative bioluminescence images are shown. *P* values were calculated by unpaired one-tailed *t*-tests; *n* = 10 mice per group from two independent experiments. **j**–**l** Data points depict biological replicates and whiskers show minimum and maximum.

We previously demonstrated that JNK signaling promotes lung metastasis via induction of the ECM glycoproteins osteopontin (SPP1) and tenascin C (TNC)^18^. These studies also revealed that JNK activity in breast cancer cells induces expression of *IL1A*/*B*. As *IL1A*/*B* expression levels were significantly greater in the highly metastatic MDA-LM2 cancer cells compared with the MDA parental cells (Fig. 5a), we hypothesized that this may be due to higher JNK activity. Indeed, a JNK response signature^18^ was significantly enriched in MDA-LM2 cells compared to MDA cells, both in vivo and in vitro (Fig. 7a and Supplementary Fig. 9f). To confirm regulation of IL-1 by JNK in breast cancer cells, we measured *IL1A/B* mRNA and secreted protein levels upon expression of a constitutively active form of JNK, consisting of a protein fusion between JNK1 and its upstream MAPK kinase (MAPKK) activator MKK7 (MKK7-JNK), or a mutated version (MKK7-JNK(mut)), in which the phosphorylation motif Thr180-Pro-Tyr182 in JNK1 has been replaced with Ala-Pro-Phe, thereby preventing its activation by MKK7 (refs. ^19,20^). In line with our previous observations, *MKK7-JNK* expression in MDA cells significantly induced both *IL1A* and *IL1B*, and this induction was blunted in *MKK7-JNK(mut)*-expressing cells (Fig. 7b and Supplementary Fig 9g). Moreover, treatment with a JNK inhibitor (JNKi) reduced endogenous levels of *IL1A/B* mRNA and secreted protein in MDA-LM2 cells (Fig. 7c, d). Induction of IL-1 expression in cancer cells by recombinant IL-1 was also dependent on JNK activity (Supplementary Fig. 9h). To determine whether JNK induces *IL1A*/*B* via the transcription factor c-Jun, we performed chromatin immunoprecipitation (ChIP). qPCR analysis of c-Jun-bound chromatin confirmed that c-Jun binds to *IL1A* and *IL1B* promoters in breast cancer cells (Fig. 7e, f and Supplementary Fig. 9i).

Consistent with JNK-driven expression of *IL1A/B* in cancer cells, treatment with CM from *MKK7-JNK*-expressing MDA breast cancer cells increased the production of *CXCL10* in fibroblasts compared with CM from MDA-control cells, and this increase was blunted when fibroblasts were treated with CM from *MKK7-JNK(mut)-*expressing cells (Fig. 7g). Importantly, these findings suggested that inhibition of JNK activity in breast cancer cells may impair their ability to induce a prometastatic crosstalk with lung fibroblasts. To test this hypothesis, we implanted MDA-LM2 breast cancer cells transduced with a control vector, MKK7-JNK or MKK7-JNK (mut), orthotopically into the fourth mammary fat pads of NSG mice. Mammary tumor growth and development of lung metastases were significantly promoted by expression of activated JNK but not with the inactive mutated version of JNK (Fig. 7h–j). To directly address the link between CXCL9/10 and JNK in metastatic colonization, we pretreated MDA-LM2 cells overexpressing *CXCL9*/*10* or a control vector (Supplementary Fig. 9j) with JNKi and injected the cells intravenously into NSG mice. In mice injected with MDA-LM2 control cells, pretreatment with JNKi significantly reduced metastatic colonization (Fig. 7k and ref. ^18^). However, in mice injected with cancer cells overexpressing *CXCL9/10*, lung colonization was not affected by JNKi pretreatment (Fig. 7l). Together, these results indicate that JNK-driven production of IL-1α/β by metastatic cancer cells induces CXCL9/10 in pulmonary MAFs to form a supportive metastatic niche.

### CXCR3 and active c-Jun mark metastasis-initiating cells

**Fig. 8:**
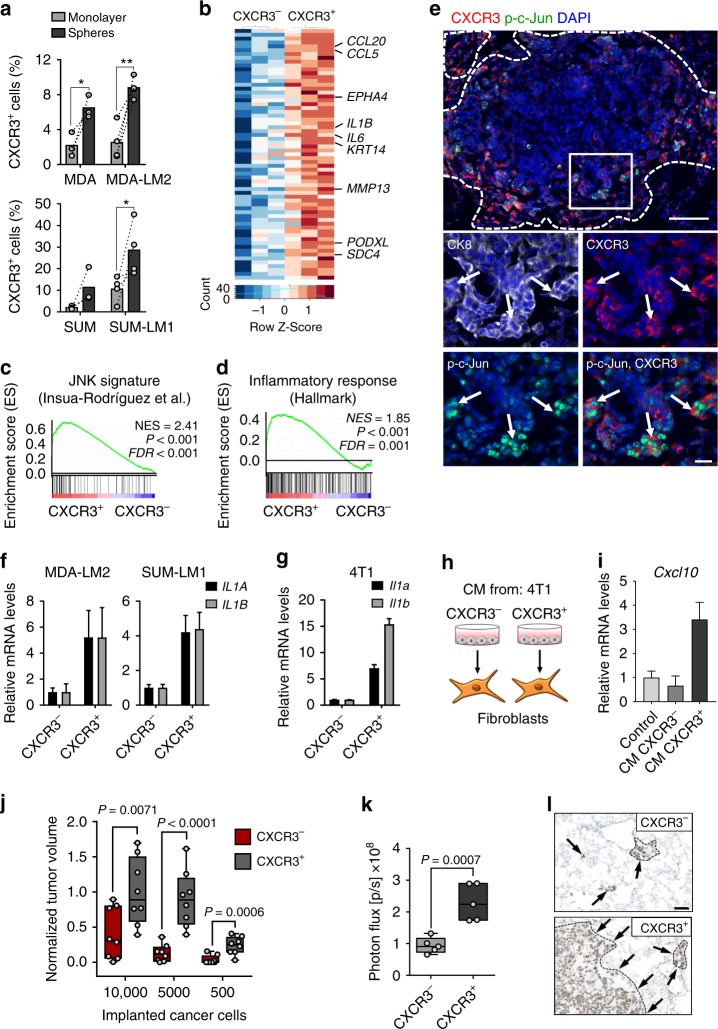
CXCR3^+^ breast cancer cells induce and benefit from paracrine crosstalk with lung fibroblasts. **a** Proportion of CXCR3^+^ MDA, MDA-LM2, SUM, and SUM-LM1 cells in monolayer or oncosphere cultures, determined by flow cytometry. Values are percentage of CXCR3^+^ cells in independent experiments. Bars show mean. *P* values were calculated by paired one-tailed *t*-tests; biological replicates, *n* = 3 (MDA, SUM); *n* = 4 (MDA-LM2, SUM-LM1). * *P* < 0.05, ** *P* < 0.01. **b** Heatmap showing normalized expression of genes induced in isolated CXCR3^+^ compared with CXCR3^–^ SUM-LM1 cells. Shown are selected genes. **c**, **d** Enrichment of indicated gene sets in CXCR3^+^ SUM-LM1 cells. NES normalized enrichment score, FDR false discovery rate. *P* values were determined by random permutation tests. **e** Immunofluorescence analysis of Cxcr3, phospho-c-Jun (p-c-Jun), cytokeratin 8 (CK8), and DAPI (nuclei) in a metastatic nodule in the lungs after intravenous injection of 4T1 cells into BALB/c mice. Scale bars, 100 μm (top), 20 μm (bottom). Dashed line indicates margin of metastasis, arrows indicate Cxcr3^+^ p-c-Jun^+^ cancer cells at the invasive front. *IL1A/B* expression in sorted CXCR3^+^ human breast cancer cells (MDA-LM2, SUM-LM1) (**f**) and in mouse mammary tumor cells (4T1) (**g**). RT-qPCR data are representative of three independent replicates each. **h** Diagram of stimulation of primary NSG mouse lung fibroblasts with conditioned medium (CM) from CXCR3^+^/CXCR3^−^ sorted 4T1 cells for 48 h. **i** *Cxcl10* expression in fibroblasts as in **h**, determined by RT-qPCR. **f**, **g**, **i** Values are mean with upper and lower limits. **j** Quantification of tumor sizes 3 weeks after subcutaneous injection of CXCR3^+^/CXCR3^−^ 4T1 cancer cells and lung fibroblasts in limiting dilutions into either flank of BALB/c mice; *n* = 8 mice per group from two independent experiments. Tumor sizes were normalized to average after co-injection of 10,000 CXCR3^+^ 4T1 cells with lung fibroblasts. **k** Bioluminescence analysis of lung colonization by sorted CXCR3^+^/CXCR3^−^ MDA-LM2 cancer cells 16 days after intravenous injection into NSG mice; *n* = 5 mice per group. **j**, **k** Data points are biological replicates. Boxes show median with upper and lower quartiles and whiskers depict maximum and minimum; *P* values were calculated by unpaired one-tailed *t*-tests. **l** Representative examples of metastases from **k** determined by human vimentin expression. Scale bar, 50 µm.

CXCR3 is the only receptor known to bind and be activated by CXCL9/10 (ref. ^21^). Interestingly, flow cytometric analysis revealed that CXCR3 is expressed by a subpopulation of MDA and SUM cancer cells and their metastatic derivatives (Fig. 8a). Moreover, the proportion of CXCR3^+^ cancer cells was significantly higher when cultured under serum-free sphere conditions, and tended to be higher in lung metastatic derivatives compared with parental counterparts (Fig. 8a). Importantly, CXCR3 was also expressed in subsets (range 3.8–11.1%) of cancer cells isolated with high purity from pleural effusions or ascites of four breast cancer patients (Supplementary Fig. 10a, b). To further characterize the CXCR3^+^ subpopulation of breast cancer cells, we established transcriptomic profiles of FACS-sorted CXCR3^+^ and CXCR3^–^ SUM-LM1 breast cancer cells (Fig. 8b and Supplementary Table 3). Intriguingly, GSEA revealed that CXCR3^+^ cancer cells had increased inflammatory- and JNK signaling and showed characteristics of basal cells and stem cells of the mammary gland (Fig. 8c, d and Supplementary Fig. 11), in line with the increase of CXCR3^+^ cells in sphere cultures (Fig. 8a). To study the relationship between CXCR3^+^ cells and JNK signaling in metastasis, we performed immunofluorescence analysis of CXCR3 and p-c-Jun expression in metastatic nodules. CXCR3 expression was particularly prominent in cells located at the invasive front of metastatic nodules and overlapped substantially with p-c-Jun expression (Fig. 8e and Supplementary Fig. 12a, b). GO term analysis of CXCR3^+^ cancer cells indicated enrichment of genes involved in inflammatory signaling and chemokine production (Supplementary Table 4). We therefore reasoned that CXCR3^+^ cancer cells may secrete higher levels of IL-1α/β, in turn leading to elevated production of *CXCL9/10* in fibroblasts. Indeed, isolated CXCR3^+^ cancer cells expressed higher levels of *IL1A*/*B* compared with CXCR3^−^ counterparts (Fig. 8f, g), and CM from CXCR3^+^ MDA-LM2 cells contained higher levels of secreted IL-1α/β (Supplementary Fig. 12c). In line with this, CM from CXCR3^+^ cancer cells, but not from CXCR3^−^ cells, induced high *CXCL10* expression in mouse and human lung fibroblasts (Fig. 8h, i and Supplementary Fig. 12d). Importantly, sorted CXCR3^+^ 4T1 cells had increased tumor-initiating ability compared with CXCR3^−^ cells when co-injected subcutaneously in limiting dilutions with lung fibroblasts, and resulting tumors were significantly larger (Fig. 8j and Supplementary Fig. 12e). Notably, this was not observed in the absence of lung fibroblasts (Supplementary Fig. 12f). Furthermore, CXCR3^+^ MDA-LM2 cells had significantly increased abilities to establish metastases in the lung microenvironment compared with CXCR3^−^ cells (Fig. 8k, l). Collectively, CXCR3^+^ metastasis-initiating cells with active JNK signaling induce a fibroblast niche in the lung. These findings underscore the importance of a paracrine interaction between breast cancer cells and fibroblasts in tumor initiation and metastasis.

### Inhibition of CXCR3 blocks metastatic colonization of the lungs

**Fig. 9:**
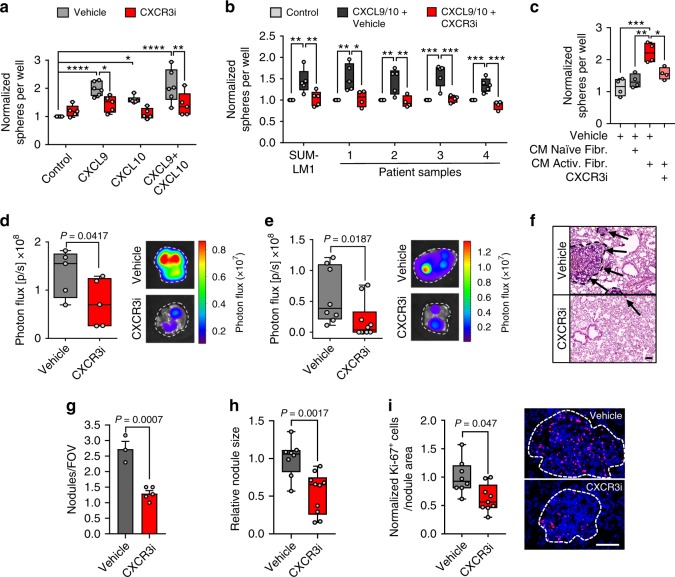
CXCR3 mediates CXCL9/10-induced oncosphere formation and can be targeted to inhibit lung metastasis. Quantification of oncospheres by MDA-LM2 cells (**a**) or SUM-LM1 cells and primary breast cancer cells from pleural fluids or ascites (**b**) after stimulation with 100 ng/ml recombinant CXCL9/10, individually or together. In addition, the cells were treated with 10 μM CXCR3 antagonist (CXCR3i) or vehicle. Biological replicates (**a**) vehicle *n* = 6, CXCR3i *n* = 5; **b** SUM-LM1 *n* = 5, patient samples, control and CXCL9/10 + vehicle *n* = 5 for each group, CXCL9/10 + CXCR3i *n* = 4. Sphere numbers were normalized to the average number in the control group. *P* values were calculated on biological replicates by ordinary one-way ANOVA with Sidak’s multiple comparisons test. **c** Quantification of MDA-LM2 oncospheres in control medium (vehicle), in conditioned medium (CM) from control MRC-5 fibroblasts (naive), or from MRC-5 cells treated with MDA-LM2 cell CM (activated), together with CXCR3i or vehicle; *n* = 4 biological replicates with 5–10 technical replicates each. *P* values were determined by ordinary one-way ANOVA with Tukey’s multiple comparisons test. **a**–**c** * *P* < 0.05, ** *P* < 0.01, *** *P* < 0.001, **** *P* < 0.0001. Lung colonization in NSG mice injected with MDA-LM2 cells (**d**) or BALB/c mice with 4T1 mouse mammary tumor cells (**e**). In both settings, the mice received systemic CXCR3i treatment. Metastatic colonization was quantified by bioluminescence after 13 days. Representative ex vivo lung bioluminescence images are shown. *P* values were calculated by unpaired one-tailed *t*-tests; *n* = 5 mice per group (**d**); *n* = 8 mice (vehicle, (**e**)), *n* = 10 mice (CXCR3i, (**e**)) pooled from two experiments. **f** Examples (H&E) of lung metastases from **e**. Scale bar, 100 μm. **g**, **h** Number and size of metastatic nodules from **e**. Average numbers of nodules per field of view (FOV) and relative nodule size are shown. Values in **g** are mean with SEM. **i** Ki-67 expression in cancer cells in lung metastases from **e**. Results were normalized to average in vehicle group. Right, representative immunofluorescence staining. Scale bar, 100 μm. **a**–**e**, **h**, **i** Boxes show median with upper and lower quartiles, data points show biological replicates, and whiskers represent minimum and maximum. **g**–**i** *P* values were calculated using unpaired two-tailed *t*-tests.

In addition to the increased ability of CXCR3^+^ cancer cells to induce *Cxcl9/10* expression in lung fibroblasts, this subpopulation of cancer cells is also likely to benefit from this crosstalk. To test whether CXCR3 is functionally required for *CXCL9/10*-mediated oncosphere formation and lung metastasis, we utilized the CXCR3 antagonist AMG-487 (CXCR3i). Stimulation of MDA-LM2, SUM-LM1, and primary patient-derived breast cancer cells with recombinant CXCL9 and/or CXCL10 increased oncosphere formation, and this was reversed by addition of CXCR3i (Fig. 9a, b). Furthermore, CM from activated fibroblasts stimulated sphere formation in a CXCR3-dependent manner (Fig. 9c). Importantly, systemic treatment of NSG mice with CXCR3i significantly diminished lung metastatic outgrowth of MDA-LM2 cells (Fig. 9d). Since CXCL9/10 are known to regulate immune responses^22,23^, and CXCR3 expression is associated with activated T cells, we analyzed CXCR3^+^ CD8a^+^ or CD4^+^ T cells by FACS in healthy and metastatic lungs from BALB/c mice. Approximately 4% and 25% of CD8a^+^ and CD4^+^ T cells, respectively, expressed CXCR3, and no increase was observed in lungs with growing 4T1 metastases (Supplementary Fig. 13a). This suggested that T-cell activation may be blocked in this context. Indeed, analysis of two immune checkpoint molecules and exhaustion markers, PD1 and Lag3, revealed increased expression of both markers in T cells from metastatic lungs compared with healthy controls (Supplementary Fig. 13b, c). This increase was also apparent when CXCR3-expressing CD8a^+^ T cells were analyzed specifically (Supplementary Fig 13d). The results indicate that T-cell responses are inhibited in these metastases, which is in line with previously observed immunoediting in basal-like breast cancer^24^. In further support of this, we found that 4T1 cancer cells express significant levels of the immune checkpoint activator PD-L1 (Supplementary Fig. 13e). Considering potential impact of the immune system, we addressed the effect of CXCR3i in an immunocompetent syngeneic mouse model. BALB/c mice were injected intravenously with 4T1 cells and concurrently treated with CXCR3i until the experimental endpoint. Lung metastatic outgrowth was also significantly repressed upon CXCR3 inhibition in the syngeneic setting (Fig. 9e, f), affecting metastatic nodule number and size (Fig. 9g, h). In line with this, cancer cell proliferation was repressed by CXCR3i treatment (Fig. 9i). Furthermore, transcriptomic analysis of *CXCL9/10*-expressing MDA-LM2 cells treated with CXCR3i revealed repression of cell cycle gene signatures (Supplementary Fig. 14a–c). Collectively, our data suggest that systemic antagonism of CXCR3 may be an effective strategy to disrupt cancer cell-fibroblast crosstalk in the lungs in basal-like breast cancers. To analyze whether CXCR3 in cancer cells is specifically required for metastatic colonization, we transduced 4T1 cells with shRNA against CXCR3 and injected the cells intravenously into BALB/c mice. CXCR3 knockdown significantly reduced lung colonization (Supplementary Fig. 15a–c).

**Fig. 10:**
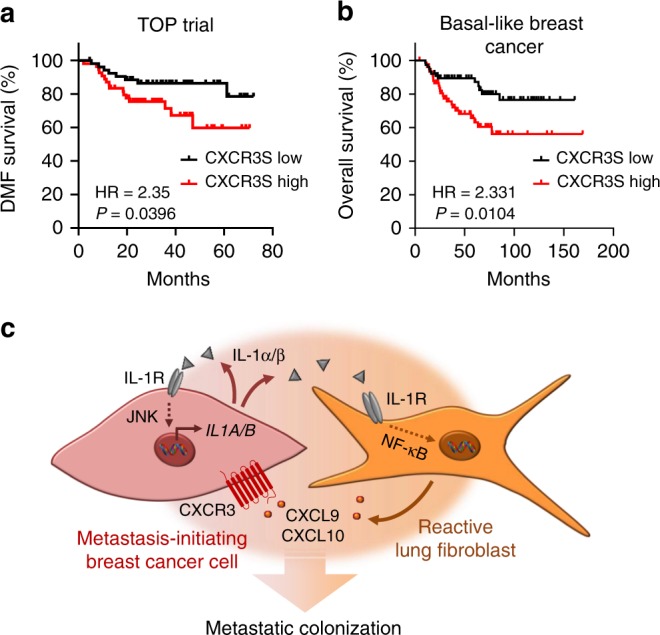
CXCR3^+^ cell signature associates with poor outcome in breast cancer patients. **a**, **b** Kaplan–Meier analyses of breast cancer patients, associating CXCR3^+^ cell signature (CXCR3S) with distant metastasis-free (DMF) survival (**a**, TOP trial data set, *n* = 107 patients) or overall survival (**b**, compiled data set from basal-like breast cancers, KM plotter, *n* = 153). Median cutoff was used to group samples into CXCR3S low and high. HR hazard ratio. *P* values were determined by log-rank test. **c** Model summarizing interactions between metastasis-initiating breast cancer cells and fibroblasts in the lungs. High JNK activity induces IL-1α/β production in metastasis-initiating cells that furthers JNK signaling in an autocrine fashion. The positive feedback loop increases IL-1α/β levels secreted by cancer cells. Fibroblasts respond to IL-1α/β via type I IL-1R signaling and NF-κB-mediated upregulation of CXCL9/10 that promote metastatic colonization via CXCR3 expressed by metastasis-initiating cells.

Finally, to address the putative link between CXCR3^+^ cancer cells and clinical prognosis, we clustered tumor samples from breast cancer patients according to the expression of CXCR3^+^ cell signature (CXCR3S) (Supplementary Table 3). High CXCR3S expression associated with poor relapse-free survival, distant metastasis-free survival, and overall survival (Fig. 10a, b; Supplementary Fig. 16a–c). Taken together, these evidences suggest that CXCR3^+^ metastasis-initiating cells with active JNK signaling not only induce CXCL9/10 in MAFs, but also take advantage of these cytokines to promote metastatic colonization.

## Discussion

Disseminated cancer cells require a supportive niche to successfully form metastases^4^. Indeed, the vast majority of cancer cells face an unfavorable microenvironment at secondary sites and are eliminated following extravasation^25,26^. Resting stroma can be resistant to the establishment of intruding cells^27,28^, and thus cancer cells that arrive in distant organs and are equipped with their own niche components or niche-promoting ability may have a selective advantage^12^. In this study, we show how the ability to induce niche formation by engaging fibroblasts fuels metastatic colonization.

JNK signaling promotes metastasis of breast cancer cells through distinct mechanisms. Our previous study showed that the development of breast cancer metastasis requires JNK-induced expression of the ECM proteins SPP1 and TNC^18^. Here, we reveal that JNK signaling also instigates communication between breast cancer cells and lung fibroblasts and enables metastatic cells to rapidly establish a supportive niche in the lung. Our findings suggest a model (summarized in Fig. 10c) in which JNK activity in metastasis-initiating breast cancer cells induces expression of IL-1α/β, which interact with IL-1R on lung fibroblasts to stimulate NF-κB-mediated induction of *Cxcl9*/*10*. Once secreted from fibroblasts, CXCL9 and CXCL10 bind to CXCR3 on the surface of a subpopulation of breast cancer cells to complete a paracrine loop that promotes initiation and growth of metastasis in the lungs. Concurrently, IL-1α/β interacts with IL-1R on cancer cells to activate an autocrine positive feedback loop. Our results establish a link between stress signaling, the ability of disseminated cancer cells to modify the microenvironment in secondary organs, and their metastatic potential.

The role of inflammatory signaling in cancer, such as IL-1 signaling, is complex and likely context-dependent. At primary sites, IL-1 has been shown to promote tumor growth^7,29^. However, at secondary sites, studies suggest both prometastatic and antimetastatic roles for IL-1 signaling in models of breast cancer^30,31^. The divergent IL-1 responses in metastasis may be explained by different breast cancer subtypes. Our study addresses basal-like breast cancer that has high propensity to metastasize to lung. NF-κB is active in basal-like breast cancer^32,33^, indicating that this subtype can adapt to and take advantage of inflammatory signaling. In line with this, studies have shown that IL-1 secretion and IL-1R response is particularly associated with basal-like (triple-negative) breast cancer compared with other subtypes^34,35^. Moreover, we have previously shown that JNK signaling, that induces IL-1α/β in breast cancer cells, is linked to basal-like breast cancer^18^. Thus, inflammatory signaling mediated by IL-1 may be beneficial for basal-like breast cancer progression and metastasis. Indeed, our results based on IL-1 or IL-1RI loss-of-function indicate that IL-1 promotes metastatic colonization of the lung.

We find that reactive fibroblasts, near or within metastatic lesions in the lung, acquire an inflammatory phenotype reminiscent of fibroblasts in wound healing and primary tumors^7,36^. For example, collagens, ECM glycoproteins (including fibronectin, TNC, and SPP1), and matrix-modifying enzymes (such as serpins and lox-family proteins) are highly induced in MAFs. We find that this inflammatory phenotype is associated with a substantial expansion of fibroblasts during growth of macrometastasis, which is likely derived from the striking increase in fibroblast proliferation. However, CAFs and MAFs represent a heterogeneous group of mesenchymal cells, including resident tissue fibroblasts, pericytes, and bone marrow-derived mesenchymal stromal cells^37^. Accordingly, the expanded fibroblast population in lung metastases may include several subtypes of fibroblasts with diverse origins and functions. Indeed, recent studies suggest that tumors may harbor a number of different fibroblast subtypes^38–40^. Thus, further studies are needed to determine the roles of distinct fibroblast subgroups in metastatic progression.

Growing evidence suggests that CXCR3 may play an important role during breast cancer progression^41–43^. We show that the ability to promote initiation of tumors and metastases is enriched in CXCR3^+^ cancer cells compared with CXCR3^–^ cells. CXCR3^+^ breast cancer cells secrete IL-1α/β to stimulate a crosstalk with lung fibroblasts from which they benefit through their CXCR3 receptor. We find that only a small subset of metastatic breast cancer cells expresses CXCR3, and this subset is characterized by high JNK activity that we previously linked to mammary stem cell properties^18^. Therefore, CXCR3^+^ cancer cells may be enriched in metastatic stem cells that are equipped to exploit the metastasis-promoting paracrine loop with fibroblasts. This conclusion is also supported by previous work showing that metastasis-initiating cells can take advantage of microenvironmental cues, such as the ECM protein periostin, that supports maintenance of stem cell properties and metastatic colonization^44^. Evidence from studies on colon cancer also indicates that fibroblasts play a major role in the maintenance of cancer stem cells^45,46^. As a notable addition to these reports, our findings demonstrate that metastatic stem cells may not only selectively exploit stromal signals at the distant site, but that they may also be selectively efficient in inducing the required stromal signals. Thus, JNK activity and CXCR3 expression may mark a unique population of breast cancer cells that strategically communicate with stromal fibroblasts to establish a supportive metastatic niche tailored to their phenotype.

Previous studies have shown that CXCL9/10-CXCR3 can mediate recruitment and activation of T cells^22^, which may inhibit tumor growth and progression to metastasis in malignancies such as melanoma^23^. Basal-like breast cancers exhibit high infiltration of T cells, however, these tumors also engage in substantial immunoediting and express protein ligands such as PD-L1 to activate immune-repressing checkpoints^24^. CXCR3 marks activated T cells and we find that CD8^+^ and CD4^+^ T cells from metastatic and healthy lungs exhibit no significant difference in CXCR3 expression, indicating a lack of T-cell activation. In line with this, we find increased expression of checkpoint regulators such as PD1 and Lag3 in T cells from metastatic nodules compared with T cells from healthy lungs. Moreover, a significant portion of 4T1 mammary cancer cells express PD-L1, further supporting the notion of T-cell repression. Interestingly, a study using the 4T1 cancer model has shown that IL-1β can cause immunosuppression in cancer by inhibiting activation of CD8^+^ T cells in parallel with PD1-mediated suppression^47^. Together, this suggests that T cells are markedly suppressed in basal-like breast cancer, and complements our results showing that systemic CXCR3 inhibition represses lung metastasis in mice.

We show that fibroblast-derived CXCL9/10 promote lung colonization by directly stimulating growth of CXCR3^+^ cancer cells. Importantly, we detect CXCR3^+^ subsets of cancer cells in primary cultures of pleural effusion and ascites samples from patients with metastatic breast cancer, indicating relevance of this molecular interaction in human metastasis. Our data, revealing that CXCL9/10 are selectively induced in fibroblasts by highly metastatic cancer cells in early metastasis, suggest that these chemokines may confer a crucial metastatic advantage to cancer cells. Ultimately, interruption of the CXCL9/10-CXCR3-mediated paracrine loop through systemic inhibition of JNK or CXCR3 represents a potential future strategy to inhibit metastatic colonization of the lungs in basal-like breast cancer.

## Methods

### Cell lines

MDA-MB-231 (MDA, ATCC), MDA-MB-231-LM2 (MDA-LM2, provided by Joan Massagué, RRID:CVCL_5998)^11^, SUM159 (SUM, Asterand Bioscience), SUM159-LM1 (SUM-LM1)^18^, and 4T1 (ATCC) cells were cultured in D10F medium, consisting of DMEM GlutaMAX medium (ThermoFisher Scientific) supplemented with 10% v/v fetal bovine serum (FBS), 50 U/ml penicillin, 50 µg/ml streptomycin (Sigma-Aldrich), and 1 µg/ml amphotericin B. E0771 cells (CH3 BioSystems) were cultured in RPMI 1640 medium supplemented with 10% v/v FBS, 50 U/ml penicillin, 50 µg/ml streptomycin (Sigma-Aldrich), and 10 mM HEPES (Sigma-Aldrich). MRC-5 cells (ATCC) and primary fibroblasts obtained from lungs of healthy NSG mice, BALB/c mice, wild type (WT) or IL-1R knockout (*Il1r1*^tm1Roml^) C57BL/6 mice 6–8 weeks of age were cultured in Minimum Essential Medium Eagle medium with alpha modification (MEMα) (ThermoFisher) supplemented with 1x MEM Non-essential Amino Acid Solution (Sigma-Aldrich), 10% v/v FBS, 50 U/ml penicillin, 50 µg/ml streptomycin, and 1 µg/ml amphotericin B (US Biological).

### Pleural effusion and ascites samples

Pleural effusion and ascites samples were obtained from breast cancer patients treated at the National Center for Tumor Diseases Heidelberg (NCT) and the Department of Gynecology at the University Clinic Mannheim. Study approval was obtained from the ethical committees of the University of Heidelberg (case number S-295/2009) and the University of Mannheim (case number 2011-380N-MA) and conformed to the principles of the WMA Declaration of Helsinki and the Department of Health and Human Services Belmont Report. Patients gave written informed consent. Samples were processed by centrifugation at 300 *g* for 5 min. Red blood cells were lysed from the pellet using ACK lysis buffer (Lonza) according to manufacturer’s instructions. Cancer cells were washed with PBS (Sigma-Aldrich) before plating for culture.

Cancer cells from pleural effusion and ascites samples were cultured in a 1:1 mix of supplemented M199 medium^48^ and modified M87 medium^49^. M199 is supplemented with 2.5% vol/vol FBS, 10 μg/ml insulin, 0.5 μg/ml hydrocortisone, 20 ng/ml epidermal growth factor (EGF, Sigma-Aldrich), 100 ng/ml cholera toxin (Sigma-Aldrich), 0.5 μg/ml amphotericin B, 2 mM l-Glutamine (Sigma-Aldrich), 50 IU/ml penicillin and 50 μg/ml streptomycin and modified M87 medium contains DMEM/F12 + Glutamax (Life Technologies) supplemented with 2% vol/vol FBS, 0.7x insulin-transferrin-selenium-x (Life Technologies), 50 IU/ml penicillin, 50 μg/ml streptomycin, 5 ng/ml EGF, 0.3 μg/ml hydrocortisone (Sigma-Aldrich), 0.5 μg/ml cholera toxin, 5 nM Triiodo-L-thyronine (T3) (Sigma-Aldrich), 0.5 nM β-estradiol (Sigma-Aldrich), 5 μM isoproterenol (Sigma-Aldrich), 50 nM ethanolamine (Sigma- Aldrich), and 50 nM phosphorylethanolamine (Sigma-Aldrich).

### Oncosphere formation

Cancer cells were seeded into 75 cm^2^ ultra-low attachment cell culture flasks (Corning) in Onco2 medium, consisting of HuMEC-medium (Invitrogen) supplemented 50 U/ml penicillin (Sigma-Aldrich), 50 μg/ml streptomycin (Sigma-Aldrich), 10 ng/ml basic fibroblast growth factor (Invitrogen), 20 ng/ml EGF (Sigma-Aldrich), 5 μg/ml human insulin (Sigma-Aldrich) and 2% vol/vol B27 (Life Technologies) at a density of 25,000 cells/ml (10 ml per flasks) and incubated at 37 °C for 1 week.

To determine the role of CXCL9/10-CXCR3 axis in oncosphere formation, cancer cells were seeded into 96-well ultra-low attachment plates (Corning) in Onco2 medium at a density of 10,000 cells/ml (200 µl per well). Overall, 10 µM CXCR3i (AMG-487, Tocris) or vehicle (0.001% DMSO in Onco2 medium) were added to the medium on day 0 (day of seeding), days 1, 4, and 6. 100 ng/ml recombinant human CXCL9 or CXCL10 (Peprotech) or vehicle (0.1 % BSA in PBS) were added on days 1, 4, and 6. For sphere formation of cancer cells overexpressing CXCL9 and/or CXCL10, cells were seeded as described above without further stimulation.

For sphere formation assays in MRC-5 CM, MDA-LM2 CM was first generated. Briefly, 1 × 10^6^ MDA-LM2 cells were seeded into 10 cm cell culture dishes into D10F medium, and replaced by serum-free MEMα medium the following day. After 48 h, CM was harvested, filtered through a 0.45 µm filter and either stored at −80 °C or used directly to stimulate MRC-5 cells. To generate activated MRC-5 CM, 0.5 × 10^6^ MRC-5 cells were seeded into 10 cm cell culture dishes. The next day, cells were washed once with PBS and then treated for 48 h with either serum-free MEMα or CM generated by MDA-LM2 cells. This treatment was intended to activate MRC-5 fibroblasts using CM from cancer cells. After stimulation of MRC-5 lung fibroblasts for 48 h, medium was removed, cells were washed twice with PBS and 7 ml of Onco2 medium supplemented with 1x Non-Essential Amino acid Solution (Sigma-Aldrich) were added to the cells. Cells were then incubated for 48 h to generate CM from naive or activated MRC-5 fibroblasts. Supernatants from this treatment were harvested, passed through a 0.45 µm filter and either stored at −80 °C or used directly for oncosphere formation assays of MDA231-LM2 cells. Oncospheres were grown for 7 days and stimulated with 0.1% DMSO (vehicle control) or 10 µM CXCR3i on day 0 (day of seeding), days 1, 4, and 6.

Spheres were counted after 7 days by using a Zeiss Primovert microscope with ×4 objective. Ten wells per condition were quantified and normalized sphere counts were quantified from at least three independent experiments, depicted as dots in graphs. Statistical significance was calculated on replicates from independent experiments.

### Stimulation of fibroblasts

Recombinant IL-1α/β or cancer cell CM were used to stimulate MRC-5 cells or primary mouse lung fibroblasts from mice (NSG, *Il1r1*^tm1Roml^ C57BL/6 or WT C57BL/6). Primary lung fibroblasts were cultured in fibroblast-specific MEMα medium. When treatments included IL-1R blockade or NF-κB inhibition, MRC-5 were seeded in the presence of 20 µg/ml anti-IL-1R1 antibody or Normal Goat IgG control (R&D), or 5 µM JSH-23 (Sigma-Aldrich) or 0.1% DMSO as a vehicle. The next day, medium was aspirated, fibroblasts were washed with 1 ml PBS per well, and 1 ml CM or serum-free MEMα were added. Overall, 1 ng/ml human recombinant IL-1α/β (Peprotech) or 0.1% BSA in PBS as carrier control as well as 20 µg/ml anti-IL-1R1/IgG control or 5 µM JSH-23/ 0.1% DMSO vehicle were added to the respective wells. After 48 h incubation at 37 °C, fibroblasts were washed with PBS and lysed in RLT buffer (Qiagen) for RNA extraction.

### Overexpression of *CXCL9/10*

Human *CXCL9* and *CXCL10* cDNA sequences flanked by XhoI and BamHI restriction sites were acquired as GeneArt™ Strings™ DNA Fragments (Invitrogen). *CXCL9* or *CXCL10* DNA strings were subcloned into the pLVX-Puro lentiviral expression vector (Clontech) via XhoI and BamHI sites. For combined overexpression of *CXCL9* and *CXCL10*, a pLVX-Hygro lentiviral vector expressing *CXCL10* was additionally generated. For this purpose, the pLVX-FADD-DD plasmid was obtained from Addgene, which was a gift from Joan Massagué (Addgene plasmid # 58263)^15^ and contains the pLVX-IRES-Hygro backbone (Clontech). The FADD-DD insert was replaced by subcloning the multiple cloning site of the pLVX-Puro backbone plasmid via the SnaBI and BamHI restriction sites. *CXCL10* cDNA sequence was inserted into pLVX-Hygro via XhoI and BamHI sites.

### *IL1A/B* knockdown

*IL1A/B* double knockdown was generated in MDA-LM2 cells with miR-E lentiviral vectors^50^ expressing shRNA against *IL1A/B* gene products. miR-E *IL1A* hairpins were produced from the StagBFPEP lentiviral vector, a modified version of the original SGEP vector kindly provided by Johannes Zuber (IMP-Research Institute of Molecular Pathology GmbH, Vienna), in which the constitutively expressed green fluorescent protein (GFP) was replaced by the tagBFP protein. miR-E *IL1B* hairpins were produced from the StdTomatoEZ lentiviral vector, a modified version of the original SGEP vector, in which the constitutively expressed GFP was replaced by the tdTomato protein and the puromycin resistance cassette was replaced by the zeocin resistance cassette. miR-E shIL1A/B oligonucleotides were designed using the shERWOOD algorithm^51^. The following hairpins were used: shIL1A: 5′-TGCTGTTGACAGTGAGCGCCCTGAGCAATGTGAAATACAATAGTGAAGCCACAGATGTATTGTATTTCACATTGCTCAGGATGCCTACTGCCTCGGA-3′, shIL1B: 5′-TGCTGTTGACAGTGAGCGCCAATAACAAGCTGGAATTTGATAGTGAAGCCACAGATGTATCAAATTCCAGCTTGTTATTGATGCCTACTGCCTCGGA-3′. Oligonucleotides were amplified by PCR using the Q5 High-Fidelity DNA Polymerase (New England Biolabs) and miRE‐Xho‐fw and miRE‐EcoOligo‐rev primers (sequences provided in Supplementary Table 5). PCR products containing shIL1A, shIL1B, and non-silencing miR-Es (controls) were subcloned into the StagBFPEP and StdTomatoEZ recipient vectors via EcoRI^−^HF and XhoI restriction sites.

### Expression of JNK fusion proteins

To express constitutively active or inactive JNK in MDA breast cancer cells, MKK7B2Jnk1a1 and MKK7B2Jnk1a1(APF) cDNAs were obtained from the expression vectors pCDNA3 Flag MKK7B2Jnk1a1 (Addgene plasmid #19726), and pCDNA3 Flag MKK7B2Jnk1a1(APF) (Addgene plasmid #19730), both kindly provided by Roger Davis, and subcloned into the pLVX-Puro lentiviral vector via BstBI and XmaI restriction sites. Restriction sites in cDNA were introduced by PCR using BstBI-MKK7Jnk1a1-Fw and XmaI-MKK7Jnk1a1-Rv primers (sequences in Supplementary Table 5). PCR products were subcloned into lentiviral pLVX-Puro vector via BstBI and XmaI restriction sites.

### Lentiviral infection

Lentiviral particles were produced by transfecting HEK293T cells with overexpression- or knockdown vectors, described above, together with pMD2G and psPAX2 packaging plasmids using Lipofectamine 2000 (Invitrogen). Supernatants containing lentiviral particles were used to infect cancer cells overnight in the presence of 8 μg/ml polybrene (Sigma-Aldrich). Infected cells were selected with 2 μg/ml puromycin (Invitrogen), 700 μg/ml hygromycin B (Life Technologies) or 0.4 mg/ml zeocin (ThermoFisher Scientific) in D10F medium for 3–7 days until uninfected control cells were dead.

### JNK inhibition

MDA-LM2 cells were seeded at a density of 250,000 cells/6-well or 1 × 10^6^ cells/10 cm dish in D10F medium containing 5 µM CC-401 (Santa Cruz Biotechnology) or 0.1% DMSO as a vehicle. The following day, medium was exchanged and cells were stimulated with fresh D10F containing 5 µM CC-401 or 0.1% DMSO as a vehicle. After 48 h, medium was removed, cells were washed in PBS, and lysed in RLT buffer (Qiagen) for RNA extraction or counted for injection.

### RNA extraction

RNA was extracted from cultured cells with the QIAGEN RNeasy Mini Kit according to the manufacturer’s protocol. RNA from FACS-isolated cells was purified using the Arcturus PicoPure Extraction Kit (ThermoFisher Scientific) according to the manufacturer’s protocol. RNA concentration and purity were measured on a Nanodrop1000 spectrophotometer (Peqlab) or Bioanalyzer 2100 (Agilent).

### RT-qPCR

cDNA was generated from total RNA using the High-Capacity cDNA Reverse Transcription Kit (Applied Biosystems) according to the manufacturer’s protocol. cDNAs corresponding to 20–40 ng RNA were used as input for qPCR. Gene expression was analyzed using SYBR Green gene expression assay (Applied Biosystems) on the ViiA 7 Real-Time PCR System (Applied Biosystems). For qPCR analysis of *Cxcl9/CXCL9* and *Cxcl10/CXCL10* mRNA levels in fibroblasts or breast cancer cells from lungs with metastases, standard amplification curves for mouse *Cxcl9*, mouse *Cxcl10*, human *CXCL9*, human *CXCL10* were first generated by running qPCR on full length cDNAs for each gene at different concentration (0.001–100 pg/ml). Generated standard curves were utilized to calculate the mRNA expression levels of mouse fibroblasts and human cancer cells, that were isolated from mouse lung with MDA-LM2 macrometastasis. A list of primers is provided in Supplementary Table 5.

### Chromatin immunoprecipitation (ChIP)

We performed ChIP on 4–5 × 10^6^ MDA-LM2 or SUM-LM1 cells using the PierceTM Magnetic ChIP Kit (ThermoFisher Scientific) according to manufacturer’s instructions with 10 µg rabbit IgG isotype control or c-Jun antibody (Cell Signaling). Primers for ChIP-qPCR were designed flanking known consensus and tracked AP-1/c-Jun binding sites in proximity to *IL1A* and *IL1B* promoter regions, as recognized by the USCS Genome Browser^52^. Primer sequences used for SYBR green (Applied Biosystems) qPCR are provided in Supplementary Table 5. qPCR was analyzed with the Viia 7 Real-Time PCR System (Applied Biosystems).

### Flow cytometry

To profile fibroblasts at different metastatic stages, mice with similar bioluminescence signals from MDA and MDA-LM2 metastasis groups were selected at weeks 1 and 3 post cancer cell injection. Lungs were digested in 0.5% (w/v) collagenase type III (Pan Biotech), 1% (w/v) Dispase II (Gibco) and 30 µg/ml DNase I in PBS for 30–45 min at 37 °C. Lungs from age-matched healthy mice were used as control group. Single cell suspensions were obtained by pipetting and filtering through 70 µm nylon filters with FACS buffer (2% FCS in PBS). Cells were pelleted and red blood cells were lysed with ACK buffer (Lonza). Lysis was stopped by FACS buffer and cell pellets were resuspended in PBS and counted using a ViCell Automated Cell Counter. Per 1 × 10^6^ cells, 100 µl FcR blocking reagent (Miltenyi BioTec, diluted to 1x in FACS buffer) were added and cells were incubated for 10 min on ice. Respective antibody cocktails were prepared in FACS buffer and added 1:1 to cells in FcR blocking reagent. The following antibodies were used at the indicated staining dilutions: CD45-PE (1:3,000, eBioscience, Cat. No. 12-0451-82), CD11b-PE (1:3,000, BD Biosciences, Cat. No. 553311), CD31-PE (1:1,000, eBioscience, Cat. No. 12-0311-83), CD326(EPCAM)-PE (1:250, eBioscience, Cat. No. 12-5791-83); CD140a-APC (1:50, eBioscience, Cat. No. 17-1401-81), CD140b-APC (1:50, eBioscience, Cat. No. 17-1402-82). Cells were stained for 30 min on ice in the dark. After staining, cells were washed three times in FACS buffer, filtered and resuspended in MEMα medium or FACS buffer containing 3 µg/ml DAPI (BioLegend). Cells were sorted on BD FACSAria1 or FACSAria2 machines and collected in 150 μl Arcturus PicoPure Extraction Buffer. Numbers of fibroblasts per lung were determined by multiplying the total cell count per lung with the percentage of PE-APC + fibroblasts as analyzed using FlowJo^TM^ V10.

Flow cytometry was used to analyze CXCR3 on cell surface and sort. Human breast cancer cells were detached using trypsin (when grown as monolayer, Sigma-Aldrich) or pelleted and dissociated using StemPro Accutase (when grown as spheres, Life Technologies), counted and stained with 0.5 µg PE-conjugated anti-human CD183 (CXCR3) antibody (1:20, BioLegend, Cat. No. 353706) or PE-conjugated mouse IgG1, κ isotype control (1:40, BioLegend, Cat. No. 400114) per 1 million cells in 100 µl FACS buffer for 30 min on ice in the dark. For staining of 4T1 mouse mammary tumor cells, 2.5 µg PE-conjugated anti-mouse CD183 (CXCR3) antibody (1:80, BioLeged, Cat. No 126506) or Armenian Hamster IgG isotype control (1:80, BioLegend, Cat. No. 400908) per 1 million cells in 100 µl FACS buffer were used. After staining, cells were washed three times in FACS buffer and resuspended in FACS buffer containing 3 µg/ml DAPI (BioLegend). Cell sorting was performed on a BD FACSAria1 machine.

For flow cytometry analysis of cytokeratin in primary cancer cells from pleural effusions and ascites of metastatic breast cancer patients, cells were trypsinized, counted, and fixed and permeabilized in 100 μl BD Cytofix/Cytoperm™ for up to 1 × 10^6^ cells for 20 min on ice, washed in 1x BD Perm/Wash Buffer, and stained with anti-cytokeratin (CK3^−^6H5)-FITC (1:10, Miltenyi, Cat. No. 130-080-101) or mouse IgG1-FITC isotype (clone IS5-21F5, 1:10, Miltenyi, Cat. No. 130-113-199) in Perm/Wash Buffer for 45 min on ice in the dark. MDA cancer cells were also stained as positive control and MRC-5 cells were stained as a negative control. Cells were finally washed three times in Perm/Wash Buffer and analyzed.

For immunophenotyping, lungs were mechanically disrupted and digested in a 1 ml mixture of 1 mg/ml collagenase A and D (Roche) and 0.4 mg/ml DNase I (Roche) in PBS at 37 °C for 120 min with 1000 rpm rotation. Cell suspension was passed through a 70 µm mesh and immunostaining was performed. Cells were first stained using the LIVE/DEAD™ Fixable Yellow Dead Cell Stain Kit (ThermoFisher Scientific, Cat. No. L34959) according to manufacturer’s instructions and combinations of the following fluorescently conjugated antibodies in PBS with 0.5% BSA were used: APC anti-mouse CD4 Antibody (1:80, clone GK1.5; BioLegend, Cat. No. 100411), PE/Cy5 anti-mouse CD8a Monoclonal Antibody (1:500, clone 53–6.7; BioLegend, Cat. No. 100709), PE/Cy7 anti-mouse CD223 (LAG-3) Antibody (1:40, clone C9B7W; BioLegend, Cat. No. 125226), Brilliant Violet 421™ anti-mouse CD279 (PD-1) Antibody (1:160, clone 29 F.1A12; BioLegend, Cat. No. 135217), APC anti-mouse CD274 (B7-H1, PD-L1) Antibody (1:80, clone 10 F.9G2; BioLegend, Cat. No. 124311); PE anti-mouse CD183 (CXCR3) Antibody (1:80, clone CXCR3–173; BioLegend, Cat. No. 126506). GFP expression was used to gate on 4T1 cancer cells. All flow cytometry that did not require cell sorting was performed on LSR Fortessa analyzers (BD Biosciences) and analysis was done using FlowJo software (Treestar).

### Enzyme-linked immunosorbent assay (ELISA)

To analyze protein levels of human IL-1α and IL-1β and mouse CXCL9 and CXCL10 in metastatic lungs, whole lungs from mice bearing MDA-LM2 macrometastases (3 weeks post tail vein injection) were harvested and lysed in 1 ml Cell Lysis Buffer 2 (R&D) using the gentleMACS Dissociator (Miltenyi Biotec). As a control, lungs from healthy age-matched mice were lysed. To detect human IL-1α and IL-1β, lung homogenates were diluted 1:1 in Calibrator Diluent RD6C, and CM from cancer cells was used without further dilution. For detection of mouse CXCL9 and CXCL10, lung homogenates were diluted 1:10 in Cell Lysis Buffer 2 (R&D). Quantikine Human IL-1α ELISA Kit (R&D), Human IL-1β/IL-1F2 Quantikine ELISA Kit (R&D), Mouse Cxcl10 DuoSet ELISA (R&D), and Mouse CXCL9 Quantikine ELISA (R&D) were used according to the manufacturer’s protocol. Standard curves were calculated in GraphPad Prism version 7.02 by linear regression. To account for initial dilution of lung homogenates, interpolated IL-1α/β concentrations need to be multiplied by 2 and interpolated CXCL9/10 concentrations need to be multiplied by 10 to determine final protein concentrations per lung. Secreted CXCL10 levels in supernatants from MRC-5 lung fibroblasts were quantified using the Human CXCL10/IP-10 Quantikine ELISA Kit (R&D Systems) according to manufacturer’s instructions. For quantification of CXCL10 levels in supernatants from MRC-5 cells pretreated with MDA-LM2 CM, MRC-5 cells were treated for 48 h with serum-free MEMα medium after being washed twice with PBS. This medium was then used for CXCL10 ELISA.

### Immunohistochemistry

Mouse lungs were fixed in formalin for 6–8 h at 4 °C, washed and incubated at 4 °C overnight in 30% Sucrose/PBS. The next day, lungs were washed, embedded in OCT (Sakura) and frozen at −80 °C. Overall, 8 µm sections were cut using a Microm HM 525 cryotome (ThermoFisher Scientific).

To analyze vimentin expression in mouse lungs or αSMA expression in human lung metastases (formalin-fixed-paraffin-embedded), sections were rehydrated by decreasing concentrations of ethanol, quenched with 3% hydrogen peroxide and antigen retrieval was carried out at 100 °C for 20 min with citrate buffer (pH 6.0, Vector Laboratories) for vimentin staining and pH 9.0 buffer (Vector Laboratories) for αSMA staining. Sections were blocked with 0.1% BSA containing 0.1% Triton-X for 2 h at room temperature, followed by incubation with respective primary antibodies (vimentin: Leica Biosystems, Cat. No. NCL-L-VIM-572, clone SRL33, 1:400; αSMA, abcam, Cat. No. ab7817, clone 1A4, 1:100,). Corresponding anti-mouse or anti-rabbit IgG biotinylated secondary antibodies and ABC avidin-biotin-DAB detection kit (Vector laboratories) were used for signal detection according to manufacturer’s instructions. Sections were counterstained with Mayer’s hematoxylin solution (Sigma-Aldrich) for 1 min, dehydrated using increasing concentrations of ethanol and mounted with Cytoseal XYL (ThermoFisher Scientific).

To determine sizes of metastatic nodules at 1 week and 3 weeks post MDA or MDA-LM2 intravenous injection, vimentin immunostaining of metastatic lungs (six mice per group) was analyzed. 7–10 pictures per lung were obtained using ×10 objective of an AxioPlan microscope (Carl Zeiss) and longest dimensions of metastatic foci were measured using the Fiji distribution of the ImageJ software^53^. For hematoxylin and eosin (HE) staining, sections were rehydrated with decreasing ethanol concentrations and stained for 6 min with Mayer’s hematoxylin solution. After washing and short incubation in ethanol supplemented with 0.3% hydrogen chloride, sections were washed in tap water and counterstained with eosin Y alcoholic solution (Sigma-Aldrich), dehydrated and cleared in xylenes (Sigma-Aldrich) before mounting in Cytoseal XYL (ThermoFisher Scientific). Stainings were analyzed on a Cell Observer motorized widefield microscope (Zeiss).

### Immunofluorescence

Mouse lungs were fixed and frozen in OCT as described above. Overall, 8 µm sections were air-dried for 2 h at room temperature and directly stained. For immunofluorescent staining of Ki-67, cytokeratin 8, CXCR3, and CD45, sections were washed three times in PBS and blocked in TNB Buffer (0.1 M Tris-HCL, pH 7.5 and 0.15 M NaCl with 0.5% w/v blocking reagent (Perkin Elmer)) for 1 h at room temperature, or blocked for 1 h in 5% chicken serum, 2% BSA, 0.1% Tween20 in PBS at RT. Afterwards, sections were washed three times in PBS and incubated with primary antibodies diluted in blocking buffer at 4 °C overnight. These antibodies were used: Recombinant anti-cytokeratin 8 antibody (EP1628Y) (abcam, Cat. No. ab53280, 1:30-1:75), Ki-67 Monoclonal Antibody (SolA15) (eBioscience, Cat. No. 14-5698-82, 1:100), CXCR3 (BioLegend, Cat. No. 126502, 1:80), p-c-JUN (Cell Signaling, Cat. No. 3270 S, 1:800). The following day, sections were washed three times in PBST (0.05% Tween20 in PBS) and diluted secondary antibodies in blocking buffer containing DAPI (1:1,000) were added per section and incubated for 1 h in the dark. For all stainings, Alexa Fluor secondary antibodies (1:500, Thermo Scientific) were used and slides were mounted in SlowFade Gold Antifade Mountant (Thermo Scientific). All images were taken using a Zeiss Cell Observer microscope and processed with FIJI (ImageJ) or ZEN Software.

To quantify Ki-67-positive cancer cells in lung metastases, 5–10 pictures of metastatic nodules were taken per sections using a ×20 objective. Numbers of Ki-67-positive cells were counted per metastatic area (calculated in ZEN image software). Ki-67/Unit area ratios were further normalized to the average in the respective control group. For quantification of lung nodule sizes, at least ten images of DAPI-stained lung sections were acquired on a Cell Observer (Zeiss) using a ×5 objective. Sizes of individual lung metastases were measured in ZEN image software as area and normalized to the average nodule size in the vehicle group.

### Mouse studies

Animal care and procedures were approved by the governmental review board of the state of Baden-Wuerttemberg, Regierungspraesidium Karlsruhe, under the authorization numbers G-51/13, G-81/16, G-289/16, G-218/16, DKFZ-299 and DKFZ-356 and followed the German legal regulations. Mouse strains used in the study: NOD.Cg-Prkdc^scid^ Il2rgtm1^Wjl^/SzJ (NSG, JAX stock #005557), BALB/c (Janvier Labs or Envigo), C57BL/6 and Il1r1^tm1Roml^ (IL-1RI^−^, JAX stock #003018, ref. ^54^). *Il1r1*^tm1Roml^ mice were backcrossed onto C57BL/6. Female mice, 6–8 weeks of age, were used for experiments. Mice were housed in individually ventilated cages under temperature and humidity control. Cages contained an enriched environment with bedding material.

For lung colonization assays, 10,000–500,000 cancer cells were injected in 100 µl PBS via the tail vein (t.v.). Human breast cancer cells and 4T1 or E0771 mouse mammary tumor cells were previously transduced with a triple reporter expressing the genes herpes simplex virus thymidine kinase 1, GFP, and firefly luciferase (Fluc)^55^, enabling bioluminescent imaging (BLI) of lung metastatic progression. For BLI, mice were injected intraperitoneally with 150 mg/kg d-luciferin (Biosynth), anesthetized using isoflurane (Orion) and imaged with IVIS Spectrum Xenogen machine (Caliper Life Sciences). Bioluminescent analysis was performed using Living Image software, version 4.4 (Caliper Life Sciences).

For in vivo CXCR3 inhibition, AMG-487 (Tocris) was reconstituted in DMSO to 8 µg/µl as stock solution and aliquots frozen at −20 °C. Prior to injection, aliquots were further diluted in 20% (2-hydroxypropyl)-β-cyclodextrin solution, resulting in a final concentration of 2.5% DMSO. DMSO/Cyclodextrin solution was used as vehicle. Mice received subcutaneous injections with 8 mg/kg AMG-487 every 12 h for the duration of the experiment, starting 12 h before t.v. injection of cancer cells.

To study tumor initiation capacities of CXCR3^+^ 4T1 mouse mammary tumor cells compared with CXCR3^−^, sorted cells were seeded in 10 cm dishes in 10 ml D10F and incubated at 37 °C overnight. The following day, 10,000, 5000 or 500 CXCR3^+^ or CXCR3^−^ 4T1 cells were prepared in PBS either alone or mixed with 100,000 BALB/c mouse lung fibroblasts. Cell suspensions of CXCR3^+^ or CXCR3^−^ 4T1 cells alone or with fibroblasts were implanted subcutaneously (s.c.) into either flanks of BALB/c mice in a 1:1 mix of PBS and matrigel. To study tumor initiation capacities of bulk 4T1 mouse mammary tumor cells with and without lung fibroblasts, 10,000, 5000, or 500 4T1 cells were prepared in PBS alone or mixed with 100,000 BALB/c mouse lung fibroblasts and implanted s.c. into either flanks of BALB/c mice in a 1:1 mix of PBS and matrigel. Mice were sacrificed after 20 days and tumor formation was recorded. To study tumor initiation capacities of MDA-LM2 cells with and without WT and IL-1R type I-deficient lung fibroblasts, 50 MDA-LM2 cells were injected s.c. alone or together with 100,000 lung fibroblasts obtained from female WT or *Il1r1*^−/−^ C57BL/6 mice. Tumor sizes were measured with a digital caliper and tumor volume was calculated as (width × length × height)/2. Tumor sizes were normalized to average tumor sizes of the group constituting the second bar shown for each experiment.

For mammary tumor growth and spontaneous lung metastasis assays in vivo, 5 × 10^5^ GFP- and luciferase-expressing MDA cancer cells were injected into the fourth mammary fat pads of NSG mice. Cells were trypsinized and suspended in a 1:1 (vol/vol) mix of growth factor-reduced matrigel (Corning) and PBS. Overall, 50 µl of this cell suspension was injected into the mammary fat pads of anaesthetized mice. Primary tumor growth was measured using a digital caliper at the indicated time points. Mice were sacrificed and lung metastatic burden was quantified by measuring ex vivo lung bioluminescent signal using an IVIS Spectrum Xenogen device (Caliper Life Sciences). Analysis and quantification of bioluminescent signal in the lungs was performed using Living Image software, version 4.4 (Caliper Life Sciences).

### Transcriptomic analysis

RNA from isolated fibroblasts was analyzed using Affymetrix GeneChip® Mouse Genome 430 2.0 Arrays after cDNA preamplification using the Ovation Pico WTA System V2 Kit (NuGEN) according to the manufacturer’s protocol. RNA from sorted MDA and MDA-LM2 cancer cells from micrometastatic lungs (1 week post injection) was analyzed using Affymetrix Human Transcriptome Array 2.0 (HTA 2.0) microarrays after cDNA amplification by applying GeneChip WT Pico Reagent Kit (Affymetrix) according to the manufacturer’s protocol. Raw CEL-files were RMA-normalized, and two-group comparisons were performed using Chipster (version 3.8.0) with empirical Bayes test and Benjamini–Hochberg correction of *P* values. RNA from sorted CXCR3^+^ or CXCR3^−^ SUM-LM1 cells was analyzed using Affymetrix Human U133 Plus 2.0 microarrays after cDNA preamplification by the 3′IVT Pico Reagent Kit (Affymetrix). To generate the CXCR3 signature, PCA clustering was performed on RMA-normalized data and the top 300 genes driving PC1 were retrieved using R (RStudio 1.0.143). The top 65 genes enriched in CXCR3^+^ SUM-LM1 cells from PCA clustering establish the CXCR3 signature. RNA from MDA cancer cells with combined overexpression of *CXCL9/10* cultured as spheres (0.5 × 10^6^ cells in 20 ml Onco2) and treated with 1 μM AMG-487 or 0.01% DMSO vehicle daily for 7 days was analyzed on Affymetrix Gene Chip human U133 Plus 2.0 Arrays.

GSEA was performed as previously described^56,57^ and nominal *P* values were calculated based on random gene set permutations with Benjamini–Hochberg correction. FDR < 0.1 was regarded as statistically significant. We compiled the GSEA signatures used in Fig. 1h and listed in Supplementary Table 1 from the Molecular Signatures Database (MSigDB) by the Broad Institute. The gene list for each signature is publicly available at: http://software.broadinstitute.org/gsea/msigdb/search.jsp. GO analysis was carried out using Database for Annotation, Visualization, and Integrated Discovery (DAVID)^58,59^.

Kaplan–Meier survival analysis was performed using the TOP trial gene expression data set from breast cancer patient samples (GSE16446) or compiled breast cancer data sets by Kaplan–Meier plotter (KM plotter)^60^. Samples were divided into CXCR3S high and low based on median cutoff, using mean expression of the 65 CXCR3S genes.

### Statistical analysis

All statistical tests used for experiments are noted in figure legends. *P* values ≤ 0.05 were considered statistically significant and statistical tests were two-tailed unless otherwise indicated. All functional in vivo experiments were based on extensive in vitro results that suggested one-directional effects, and thus one-tailed *t*-tests were used when analyzing tumor burden and metastasis in mice. Ratio-paired one-tailed *t*-tests were used to analyze changes in *CXCL9/10* expression in cultured fibroblasts upon combination of a stimulus (CM or recombinant cytokines) with a blocking treatment (NF-κBi, IL-1Rab, *IL1A/B* knockdown) to account for differences in baseline fold change compared with the control group. For Kaplan–Meier analyses of breast cancer patients (GSE16446), statistical differences in survival curves were calculated by log-rank (Mantel–Cox) test. Patients were classified as CXCR3S high or low based on mean normalized expression of the 65 genes comprising the CXCR3 signature, with median cutoff. The GSE14020 gene expression data set was used to study the correlation between *CXCL9* and *CXCL10* expression as well as mean expression of *CXCL9/10* and *IL1A/B*. Maximum probe sets were analyzed: *CXCL9*: 203915_at, CXCL10: 204533_at, *IL1A*: 208200_at, *IL1B*: 205067_at. Gene expression values for each gene or gene pair within each sample were associated by linear regression using Pearson correlation coefficient (*r*). Statistical analyses of microarray data generated in this study were calculated with Chipster. Statistical analyses of GO was calculated DAVID^58,59^, and statistical analysis of enrichment of gene sets was conducted using GSEA^56,57^. For GSEA, an FDR < 0.1 was considered statistically significant. All other statistical analyses were performed using GraphPad Prism version 7.02 for Windows.

### Reporting summary

Further information on research design is available in the Nature Research Reporting Summary linked to this article.

## Supplementary information

Supplementary Information

Reporting Summary

## Data Availability

All transcriptomic data sets generated for this study have been deposited to the NCBI Gene Expression Omnibus (GEO) with the accession code GSE121947. For survival analysis of breast cancer patients, the TOP trial data set was used (GSE16446) or compiled data sets by Kaplan–Meier plotter (KM plotter). The following data sets were used in KM plotter: Overall survival analysis — GSE1456, GSE16446, GSE16716, GSE20685, GSE20711, GSE3494, GSE37946, GSE42568, GSE45255, and GSE7390; distant metastasis-free survival analysis — GSE11121, GSE16446, GSE19615, GSE20685, GSE26971, GSE2990, GSE3494, GSE45255, GSE7390, and GSE9195; relapse-free survival analysis — E-MTAB-365, GSE11121, GSE12276, GSE1456, GSE16391, GSE16446, GSE16716, GSE17705, GSE19615, GSE2034, GSE20685, GSE20711, GSE21653, GSE2603, GSE2990, GSE31519, GSE3494, GSE37946, GSE42568, GSE45255, GSE4611, GSE5327, GSE7390, and GSE9195. GSE14020 was used for correlation analysis in breast cancer metastases samples. ECM gene collections were obtained from the matrisomeproject.edu^61^. All other data supporting the findings of this study are available within the article and its Supplementary information files and on reasonable request from the corresponding author (T.O.).

## References

[1] Lambert AW, Pattabiraman DR, Weinberg RA (2017). Emerging biological principles of metastasis. Cell.

[2] Joyce JA, Pollard JW (2009). Microenvironmental regulation of metastasis. Nat. Rev. Cancer.

[3] Vanharanta S, Massague J (2013). Origins of metastatic traits. Cancer Cell.

[4] Oskarsson T, Batlle E, Massague J (2014). Metastatic stem cells: sources, niches, and vital pathways. Cell Stem Cell.

[5] Plaks V, Kong NW, Werb Z (2015). The cancer stem cell niche: how essential is the niche in regulating stemness of tumor cells?. Cell Stem Cell.

[6] Kalluri R (2016). The biology and function of fibroblasts in cancer. Nat. Rev. Cancer.

[7] Erez N, Truitt M, Olson P, Arron ST, Hanahan D (2010). Cancer-associated fibroblasts are activated in incipient neoplasia to orchestrate tumor-promoting inflammation in an NF-kappaB-dependent manner. Cancer Cell.

[8] Orimo A (2005). Stromal fibroblasts present in invasive human breast carcinomas promote tumor growth and angiogenesis through elevated SDF-1/CXCL12 secretion. Cell.

[9] Scherz-Shouval R (2014). The reprogramming of tumor stroma by HSF1 is a potent enabler of malignancy. Cell.

[10] Avgustinova A (2016). Tumour cell-derived Wnt7a recruits and activates fibroblasts to promote tumour aggressiveness. Nat. Commun..

[11] Minn AJ (2005). Genes that mediate breast cancer metastasis to lung. Nature.

[12] Oskarsson T (2011). Breast cancer cells produce tenascin C as a metastatic niche component to colonize the lungs. Nat. Med..

[13] Kaplan RN (2005). VEGFR1-positive haematopoietic bone marrow progenitors initiate the pre-metastatic niche. Nature.

[14] Erler JT (2009). Hypoxia-induced lysyl oxidase is a critical mediator of bone marrow cell recruitment to form the premetastatic niche. Cancer Cell.

[15] Valiente M (2014). Serpins promote cancer cell survival and vascular co-option in brain metastasis. Cell.

[16] Del Pozo Martin Y (2015). Mesenchymal cancer cell-stroma crosstalk promotes niche activation, epithelial reversion, and metastatic colonization. Cell Rep..

[17] Dontu G (2003). In vitro propagation and transcriptional profiling of human mammary stem/progenitor cells. Genes Dev..

[18] Insua-Rodriguez J (2018). Stress signaling in breast cancer cells induces matrix components that promote chemoresistant metastasis. EMBO Mol. Med..

[19] Lei K (2002). The Bax subfamily of Bcl2-related proteins is essential for apoptotic signal transduction by c-Jun NH(2)-terminal kinase. Mol. Cell Biol..

[20] Derijard B (1994). JNK1: a protein kinase stimulated by UV light and Ha-Ras that binds and phosphorylates the c-Jun activation domain. Cell.

[21] Van Raemdonck K, Van den Steen PE, Liekens S, Van Damme J, Struyf S (2015). CXCR3 ligands in disease and therapy. Cytokine Growth Factor Rev..

[22] Whiting D (2004). Chemokine monokine induced by IFN-gamma/CXC chemokine ligand 9 stimulates T lymphocyte proliferation and effector cytokine production. J. Immunol..

[23] Barreira da Silva R (2015). Dipeptidylpeptidase 4 inhibition enhances lymphocyte trafficking, improving both naturally occurring tumor immunity and immunotherapy. Nat. Immunol..

[24] Bianchini G, Balko JM, Mayer IA, Sanders ME, Gianni L (2016). Triple-negative breast cancer: challenges and opportunities of a heterogeneous disease. Nat. Rev. Clin. Oncol..

[25] Allan AL, Vantyghem SA, Tuck AB, Chambers AF (2006). Tumor dormancy and cancer stem cells: implications for the biology and treatment of breast cancer metastasis. Breast Dis..

[26] Luzzi KJ (1998). Multistep nature of metastatic inefficiency: dormancy of solitary cells after successful extravasation and limited survival of early micrometastases. Am. J. Pathol..

[27] Bissell MJ, Hines WC (2011). Why don’t we get more cancer? A proposed role of the microenvironment in restraining cancer progression. Nat. Med..

[28] Pein M, Oskarsson T (2015). Microenvironment in metastasis: roadblocks and supportive niches. Am. J. Physiol. Cell Physiol..

[29] Biffi G (2019). IL-1-induced JAK/STAT signaling is antagonized by TGF-beta to shape CAF heterogeneity in pancreatic ductal adenocarcinoma. Cancer Discov.

[30] Castano Z (2018). IL-1beta inflammatory response driven by primary breast cancer prevents metastasis-initiating cell colonization. Nat. Cell Biol..

[31] Coffelt SB (2015). IL-17-producing gammadelta T cells and neutrophils conspire to promote breast cancer metastasis. Nature.

[32] Yamaguchi N (2009). Constitutive activation of nuclear factor-kappaB is preferentially involved in the proliferation of basal-like subtype breast cancer cell lines. Cancer Sci..

[33] Wang J (2012). FOXC1 regulates the functions of human basal-like breast cancer cells by activating NF-kappaB signaling. Oncogene.

[34] Vilsmaier T (2016). Influence of circulating tumour cells on production of IL-1alpha, IL-1beta and IL-12 in sera of patients with primary diagnosis of breast cancer before treatment. Anticancer Res..

[35] Wu TC (2018). IL1 receptor antagonist controls transcriptional signature of inflammation in patients with metastatic breast cancer. Cancer Res..

[36] Schafer M, Werner S (2008). Cancer as an overhealing wound: an old hypothesis revisited. Nat. Rev. Mol. Cell Biol..

[37] LeBleu V. S., Kalluri R (2018). A peek into cancer-associated fibroblasts: origins, functions and translational impact. Dis. Models Mech..

[38] Costa A (2018). Fibroblast heterogeneity and immunosuppressive environment in human breast cancer. Cancer Cell.

[39] Ohlund D (2017). Distinct populations of inflammatory fibroblasts and myofibroblasts in pancreatic cancer. J. Exp. Med..

[40] Raz Y (2018). Bone marrow-derived fibroblasts are a functionally distinct stromal cell population in breast cancer. J. Exp. Med..

[41] Walser TC (2006). Antagonism of CXCR3 inhibits lung metastasis in a murine model of metastatic breast cancer. Cancer Res..

[42] Ma X (2009). CXCR3 expression is associated with poor survival in breast cancer and promotes metastasis in a murine model. Mol. Cancer Ther..

[43] Chaturvedi P (2013). Hypoxia-inducible factor-dependent breast cancer-mesenchymal stem cell bidirectional signaling promotes metastasis. J. Clin. Invest..

[44] Malanchi I (2011). Interactions between cancer stem cells and their niche govern metastatic colonization. Nature.

[45] Lenos KJ (2018). Stem cell functionality is microenvironmentally defined during tumour expansion and therapy response in colon cancer. Nat. Cell Biol..

[46] Vermeulen L (2010). Wnt activity defines colon cancer stem cells and is regulated by the microenvironment. Nat. Cell Biol..

[47] Kaplanov I (2019). Blocking IL-1beta reverses the immunosuppression in mouse breast cancer and synergizes with anti-PD-1 for tumor abrogation. Proc. Natl Acad. Sci. USA.

[48] Gomis RR, Alarcon C, Nadal C, Van Poznak C, Massague J (2006). C/EBPbeta at the core of the TGFbeta cytostatic response and its evasion in metastatic breast cancer cells. Cancer Cell.

[49] DeRose Y. S. (2013). Patient-derived models of human breast cancer: protocols for in vitro and in vivo applications in tumor biology and translational medicine. Curr. Protoc. Pharmacol..

[50] Fellmann C (2013). An optimized microRNA backbone for effective single-copy RNAi. Cell Rep..

[51] Knott SRV (2014). A computational algorithm to predict shRNA potency. Mol. Cell.

[52] Kent WJ (2002). The human genome browser at UCSC. Genome Res.

[53] Schindelin J (2012). Fiji: an open-source platform for biological-image analysis. Nat. Methods.

[54] Labow M (1997). Absence of IL-1 signaling and reduced inflammatory response in IL-1 type I receptor-deficient mice. J. Immunol..

[55] Ponomarev V (2004). A novel triple-modality reporter gene for whole-body fluorescent, bioluminescent, and nuclear noninvasive imaging. Eur. J. Nucl. Med Mol. Imaging.

[56] Subramanian A (2005). Gene set enrichment analysis: a knowledge-based approach for interpreting genome-wide expression profiles. Proc. Natl Acad. Sci. USA.

[57] Mootha VK (2003). PGC-1alpha-responsive genes involved in oxidative phosphorylation are coordinately downregulated in human diabetes. Nat. Genet..

[58] Huang da W, Sherman BT, Lempicki RA (2009). Bioinformatics enrichment tools: paths toward the comprehensive functional analysis of large gene lists. Nucleic Acids Res..

[59] Huang da W, Sherman BT, Lempicki RA (2009). Systematic and integrative analysis of large gene lists using DAVID bioinformatics resources. Nat. Protoc..

[60] Gyorffy B (2010). An online survival analysis tool to rapidly assess the effect of 22,277 genes on breast cancer prognosis using microarray data of 1,809 patients. Breast Cancer Res. Treat..

[61] Naba A (2012). The matrisome: in silico definition and in vivo characterization by proteomics of normal and tumor extracellular matrices. Mol. Cell. Proteom..

[62] Wunder C (2006). Cholesterol glucosylation promotes immune evasion by Helicobacter pylori. Nat. Med..

[63] Finak G (2008). Stromal gene expression predicts clinical outcome in breast cancer. Nat. Med..

[64] Mahajan VB, Wei C, McDonnell PJ (2002). Microarray analysis of corneal fibroblast gene expression after interleukin-1 treatment. Investig. Ophthalmol. Vis. Sci..

