# Supplementary material for: Metastasis-initiating cells induce and exploit a fibroblast niche to fuel malignant colonization of the lungs
Source: Nat Commun. 2020 Mar 20;11:1494. doi: 10.1038/s41467-020-15188-x (PMC7083860; doi:10.1038/s41467-020-15188-x)
Supplement: Supplementary file 1 — Supplementary Information [file 41467_2020_15188_MOESM1_ESM.pdf]

Supplementary information

Metastasis-initiating cells induce and exploit a fibroblast niche to fuel malignant colonization of the lungs

Pein *et al*

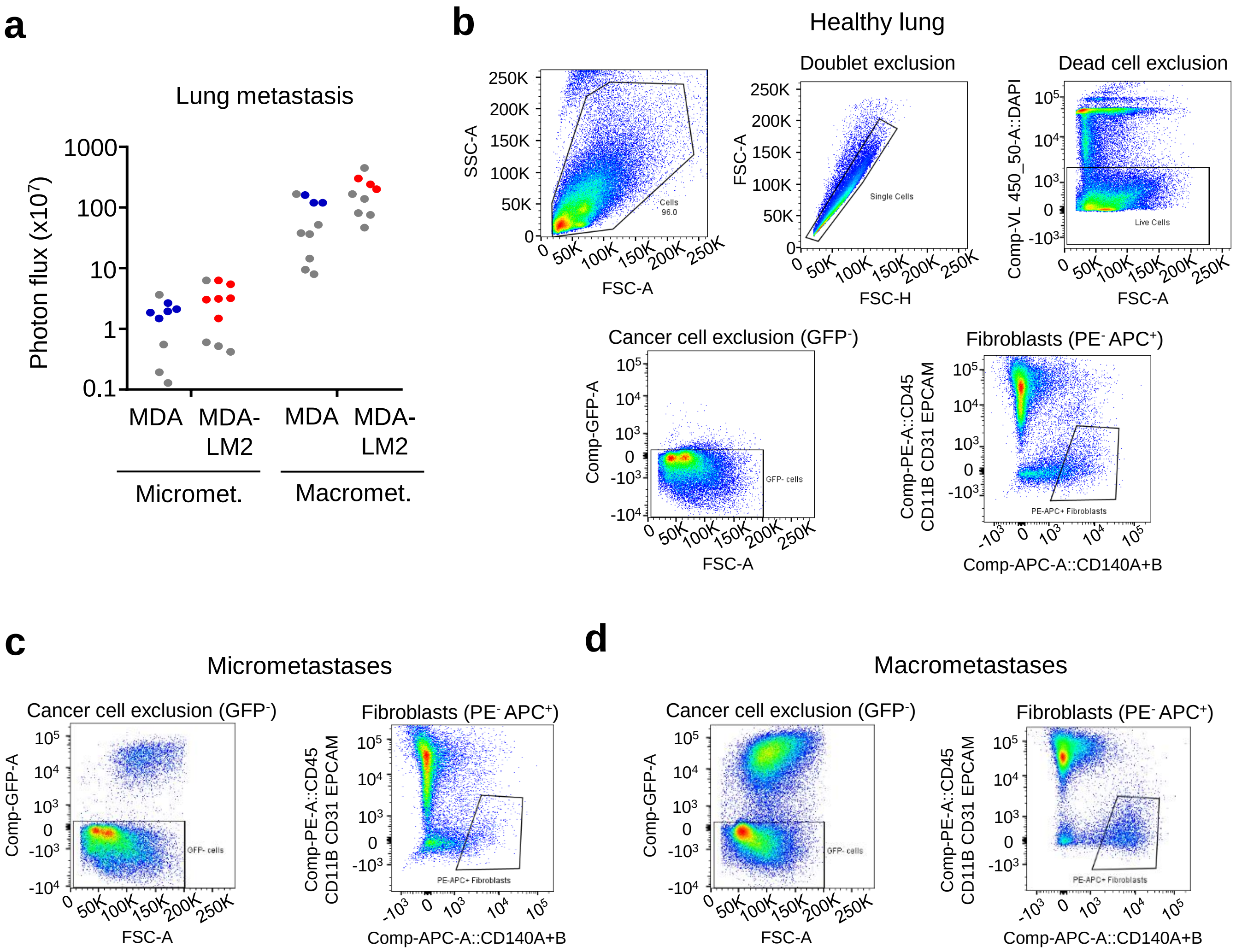

Supplementary Figure 1. Isolation of fibroblasts from healthy mouse lungs and lungs with growing metastases.

a, Lung bioluminescence in mice harboring MDA/MDA-LM2 micro- or macrometastases. Mice selected for fibroblast isolation and transcriptomic screen are labelled in blue (MDA) or red (MDA-LM2). Each point represents an individual mouse. **b-d**, FACS gating strategy used to isolate fibroblasts from healthy lungs (b) and lungs with metastases (c,d). Dead cells were excluded by DAPI staining, cancer cells were excluded by green fluorescent protein (GFP) expression, fibroblasts were further enriched by excluding CD45-, CD11b-, EPCAM- and CD31-expressing cells (PE staining) and by gating on CD140a- or CD140b-expressing cells (APC staining). PE - phycoerythrin, APC - allophycocyanin.

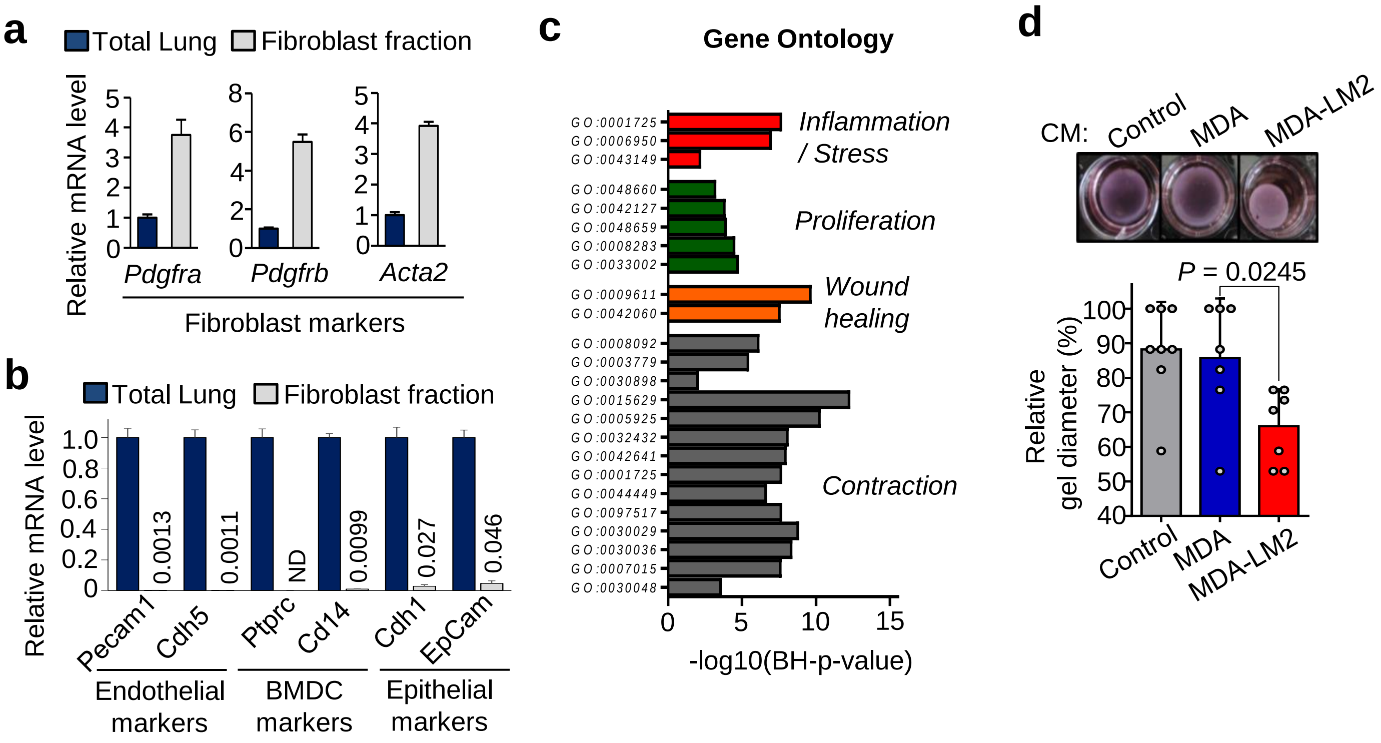

Supplementary Figure 2. Purity- and functional analyses of metastasis-associated fibroblasts.

a,**b**, Expression of indicated marker genes of fibroblasts (a), endothelial cells, bone-marrow-derived cells (BMDCs), and epithelial cells (b) determined by RT-qPCR in RNA from three lungs harboring MDA-LM2 macrometastases (Total Lung) as compared to the sorted fibroblast fraction. Expression of each target is normalized to expression in total lung. Bars show mean with SEM. Indicated numbers in fibroblast fraction in (b) show average relative expression level of each gene. ND, not detected. **c**, Gene ontology analysis of top upregulated genes accounting for shift in PCA in fibroblasts from MDA- versus MDA-LM2-micrometastases as shown in Figure 1g. **d**, Contraction of collagen gels by mouse lung fibroblasts stimulated with conditioned medium (CM) from MDA, MDA-LM2 and control medium. Control, $n = 8$, MDA or MDA-LM2, $n = 7$ for each group. Data points show biological replicates and bars depict mean with SD. P value was calculated by unpaired two-tailed t-test.

a

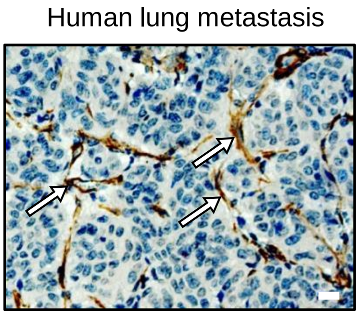

b

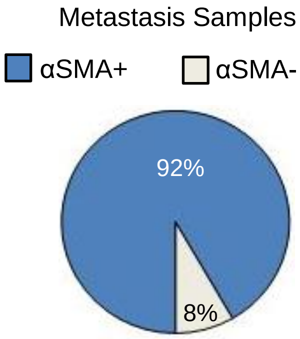

c

Patient	αSMA^{+} fibroblasts in lung metastasis
**1**	2
**2**	0-1
**3**	2
**4**	2
**5**	2
**6**	2
**7**	2
**8**	2
**9**	2
**10**	2
**11**	2
**12**	2

0, no staining; 1, moderate staining; 2, strong staining

Supplementary Figure 3. Reactive fibroblasts in lung metastases of human breast cancer.
a-**c**, Immunohistochemical analysis of alpha smooth muscle actin (αSMA) in human breast cancer metastases in lungs. Representative example of αSMA-expressing fibroblasts (a, white arrows). Scale bar, 20 µm. αSMA expression in lung metastases was analyzed from 12 breast cancer patients.

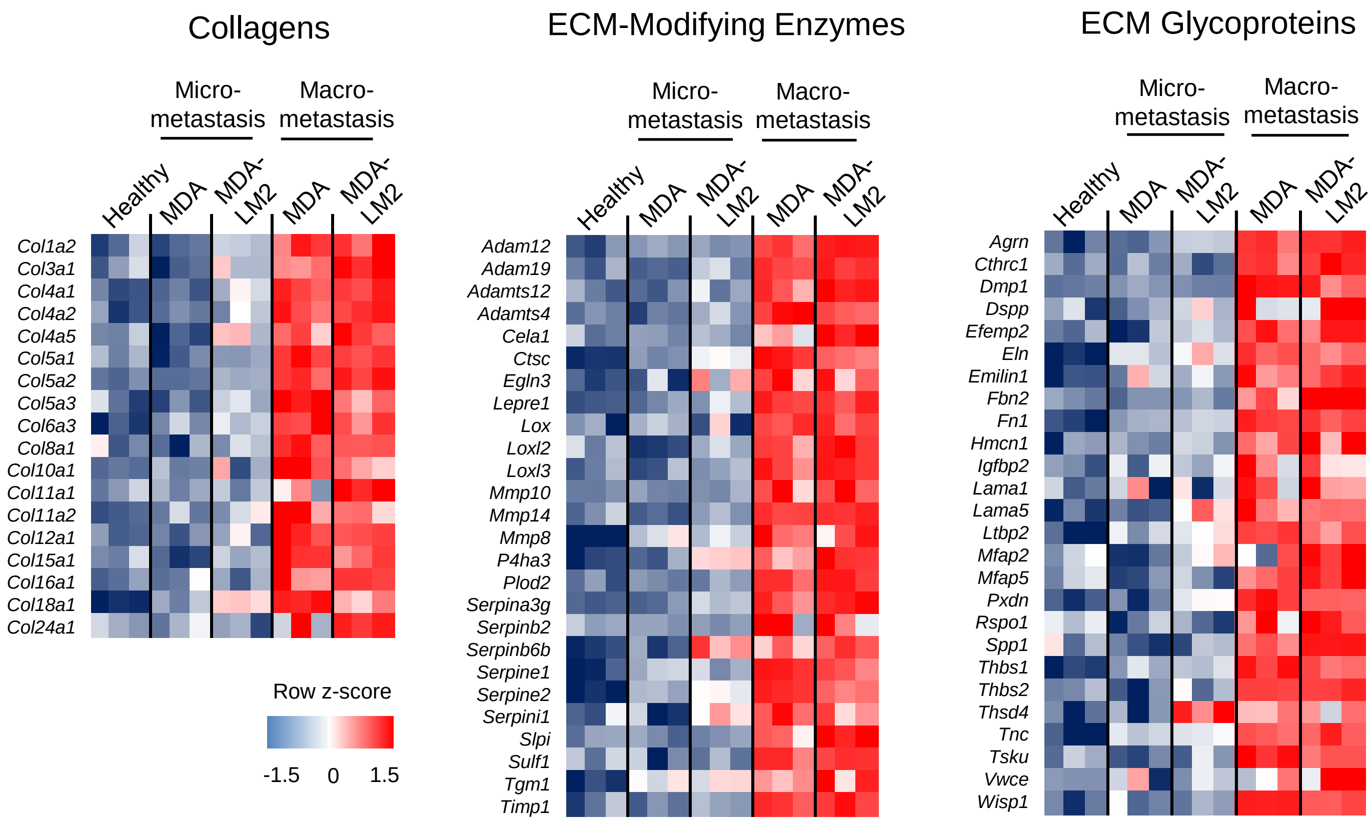

Supplementary Figure 4. Expression of extracellular matrix (ECM) and associated proteins in MAFs.
Heatmaps of normalized expression of genes encoding collagens, ECM-modifying enzymes, and ECM glycoproteins in isolated fibroblasts of healthy lungs and lungs harboring micro- or macrometastases established by MDA or MDA-LM2 breast cancer cells. Gene collections were obtained from the matrisomeproject.mit.edu[1].

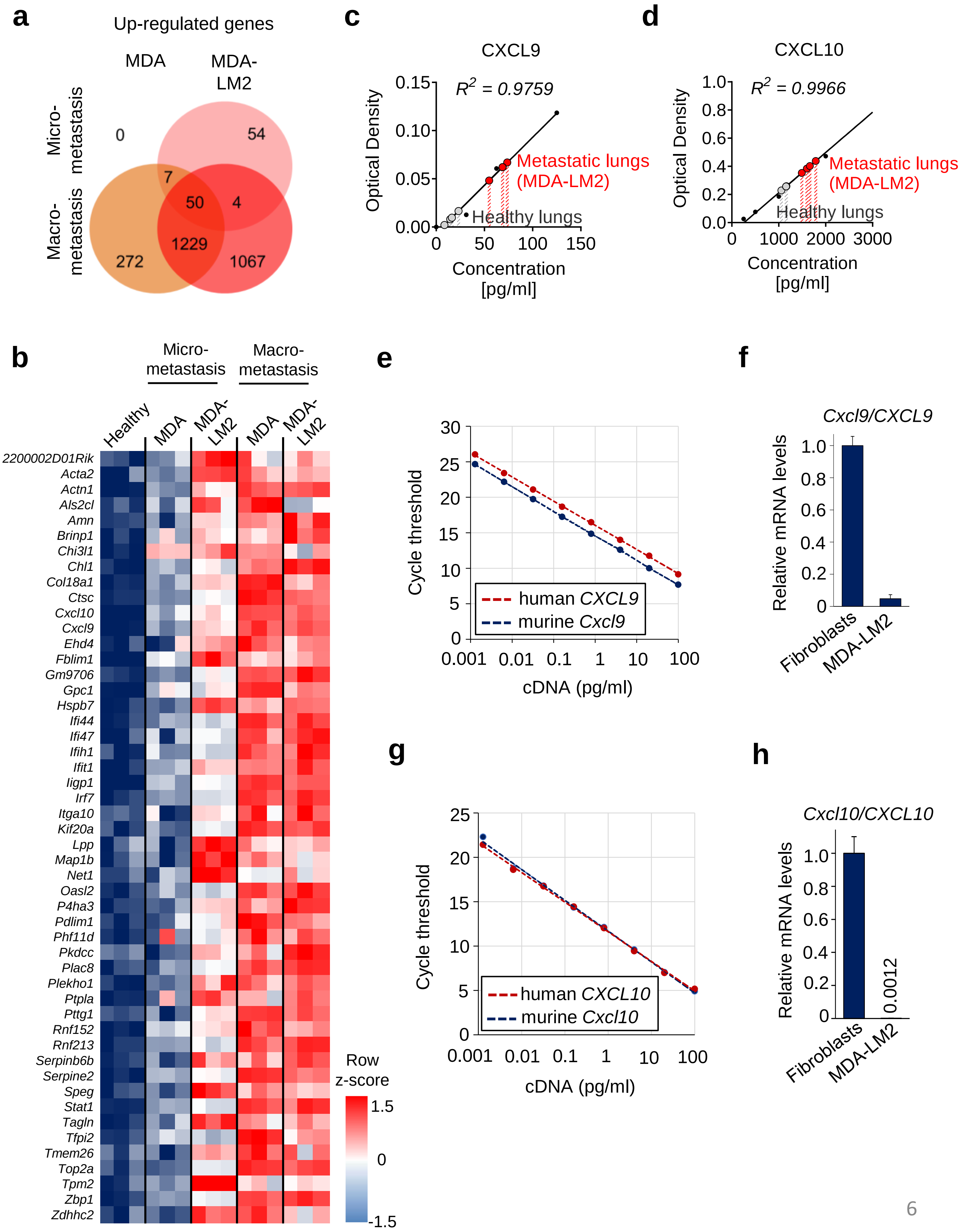
a
Up-regulated genes
MDA
MDA-LM2
Micro-metastasis
Macro-metastasis
0
54
7
50
4
1229
272
1067
b
Micro-metastasis
Macro-metastasis
Healthy
MDA
MDA-LM2
MDA
MDA-LM2
2200002D01Rik
Acta2
Actn1
Als2cl
Amn
Brinp1
Chi3l1
Chl1
Col18a1
Ctsc
Cxcl10
Cxcl9
Ehd4
Fblim1
Gm9706
Gpc1
Hspb7
Ifi44
Ifi47
Ifih1
Ifit1
Iigp1
Irf7
Itga10
Kif20a
Lpp
Map1b
Net1
Oasl2
P4ha3
Pdlim1
Phf11d
Pkdcc
Plac8
Plekho1
Ptpla
Pttg1
Rnf152
Rnf213
Serpinb6b
Serpine2
Speg
Stat1
Tagln
Tfpi2
Tmem26
Top2a
Tpm2
Zbp1
Zdhhc2
Row z-score
1.5
0
-1.5
c
CXCL9
R^2 = 0.9759
Optical Density
0.15
0.10
0.05
0.00
0
50
100
150
Metastatic lungs (MDA-LM2)
Healthy lungs
Concentration [pg/ml]
d
CXCL10
R^2 = 0.9966
Optical Density
1.0
0.8
0.6
0.4
0.2
0.0
0
1000
2000
3000
Metastatic lungs (MDA-LM2)
Healthy lungs
Concentration [pg/ml]
e
Cycle threshold
30
25
20
15
10
5
0
0.001
0.01
0.1
1
10
100
cDNA (pg/ml)
human CXCL9
murine Cxcl9
f
Cxcl9/CXCL9
Relative mRNA levels
1.0
0.8
0.6
0.4
0.2
0
Fibroblasts
MDA-LM2
g
Cycle threshold
25
20
15
10
5
0
0.001
0.01
0.1
1
10
100
cDNA (pg/ml)
human CXCL10
murine Cxcl10
h
Cxcl10/CXCL10
Relative mRNA levels
1.0
0.8
0.6
0.4
0.2
0
0.0012
Fibroblasts
MDA-LM2

Supplementary Figure 5. Gene signature upregulated in MAFs coding membrane-bound surface proteins or secreted proteins.

a, Venn diagram indicating the number of genes significantly induced (BH-*P* value < 0.05) in fibroblasts isolated from lungs harboring MDA/MDA-LM2 micro- or macrometastasis. **b**, Heatmap of normalized gene expression in fibroblasts from healthy lungs and lungs harboring MDA/MDA-LM2 micro- or macrometastases. Shown are genes significantly induced (BH-*P* value < 0.05) in fibroblasts from lungs with MDA-LM2 micrometastasis that overlap with general induction at macrometastatic stage. **c**,**d**, CXCL9 and CXCL10 protein levels in lung homogenates from n = 4 healthy mice and n = 4 mice harboring MDA-LM2 macrometastases. Cytokine levels were determined by ELISA on samples diluted 1:10. **e**, Standard expression curves of murine *Cxcl9* and human *CXCL9*. **f**, Quantification of relative *CXCL9* mRNA levels in MDA-LM2 cancer cells compared to expression of *Cxcl9* levels in isolated fibroblasts from lungs harboring MDA-LM2 macrometastases. **g**, Standard curves of murine *Cxcl10* and human *CXCL10.* **h**, Quantification of relative *CXCL10* mRNA levels in MDA-LM2 cancer cells compared to expression of *Cxcl10* levels in sorted fibroblasts from lungs harboring MDA-LM2 macrometastases; 0.0012 is the average relative expression level in MDA-LM2 cancer cells. For panels (f,h), bars depict mean with SEM.

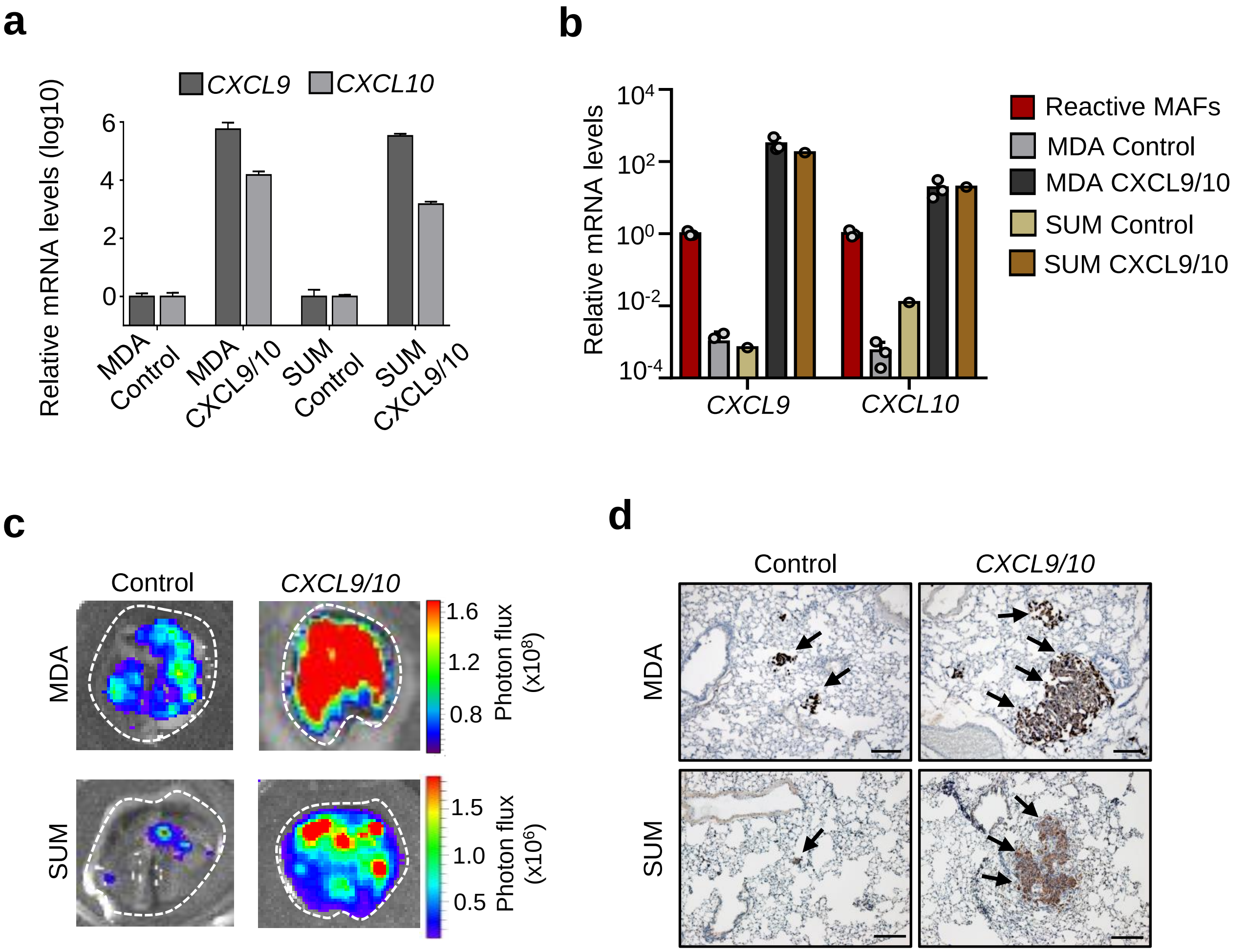

Supplementary Figure 6. Ectopic *CXCL9/10* expression in breast cancer cells and examples of resulting lung metastases in mice.

a, *CXCL9 and CXCL10* mRNA levels in MDA or SUM breast cancer cells upon combined overexpression of *CXCL9/10*. Expression levels were determined by RT-qPCR. Bars show mean with upper and lower limits. **b**, Relative expression levels of *CXCL9* and *CXCL10* in MDA or SUM breast cancer cells upon overexpression of *CXCL9/10* in comparison to expression levels of *Cxcl9/10* in fibroblasts isolated from lungs harboring MDA-LM2 metastases (Reactive MAFs). Standard curves were used to compare efficiency of murine and human *Cxcl9/10* primers. The following formula were used for calculation of expression levels using qPCR Ct values: human *CXCL9:* y = 63529e-0.673x, murine *Cxcl9*: y = 16015e-0.665x, human *CXCL10*: y = 2939.3e-0.688x, murine *Cxcl10*: y = 2247.1e-0.661x. Expression was analyzed by RT-qPCR. Bars depict mean of three biological replicates with SD. **c**,**d**, Examples of bioluminescence (c) or histology (d) from lungs of mice injected intravenously with MDA or SUM breast cancer cells overexpressing *CXCL9/10* or a control vector as shown in Fig. 3d,e. Immunohistochemistry was used to visualize human vimentin expression as a marker of cancer cells. Arrows indicate metastatic foci. Scale bars, 200 µm.

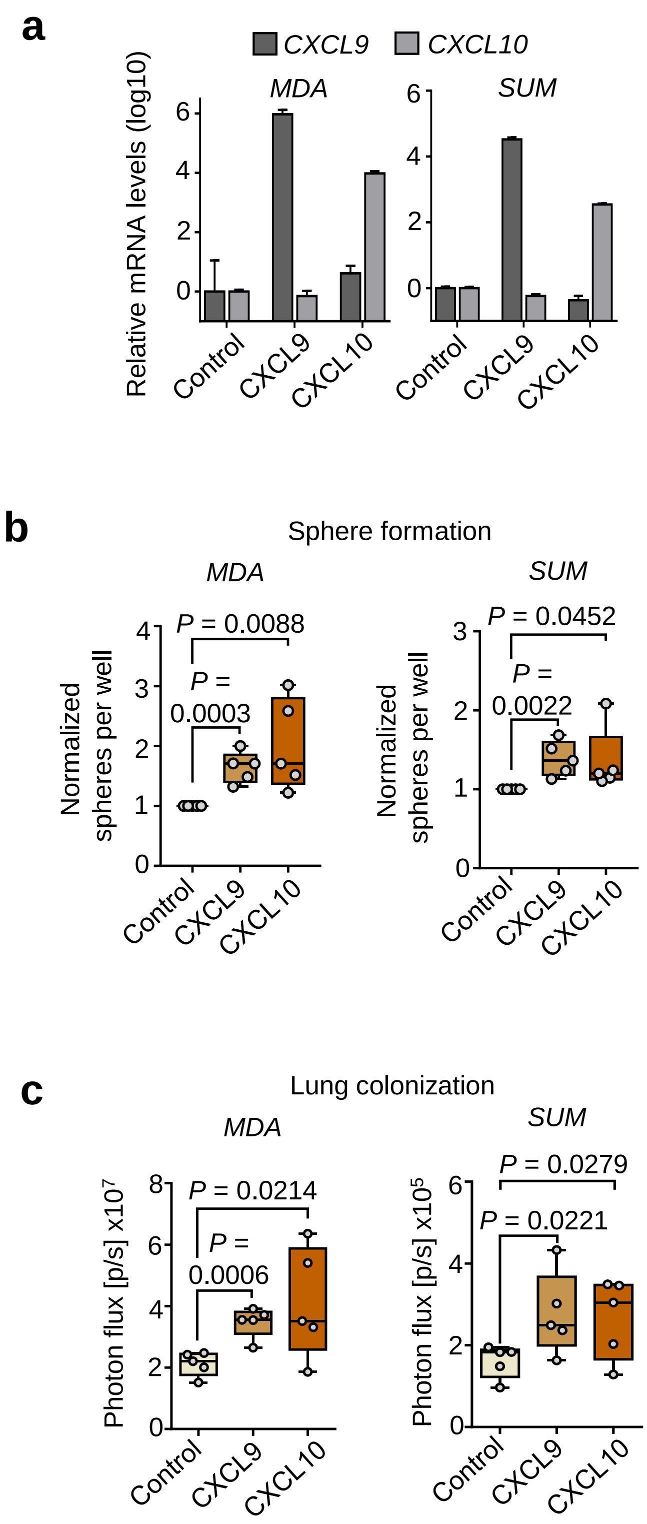
a
CXCL9
CXCL10
MDA
SUM
Relative mRNA levels (log10)
Control
CXCL9
CXCL10
b
Sphere formation
MDA
SUM
Normalized spheres per well
P = 0.0088
P = 0.0003
P = 0.0452
P = 0.0022
c
Lung colonization
MDA
SUM
Photon flux [p/s] x10^7
Photon flux [p/s] x10^5
P = 0.0214
P = 0.0006
P = 0.0279
P = 0.0221

Supplementary Figure 7. *CXCL9* and *CXCL10* individually expressed in breast cancer cells promote oncosphere formation and lung colonization.

a, *CXCL9* and *CXCL10* expression in MDA and SUM breast cancer cells transduced with cDNA of either gene. mRNA levels were analyzed by RT-qPCR. Bars show mean with upper and lower limits. **b**, Formation of oncospheres by MDA or SUM breast cancer cells overexpressing *CXCL9* or *CXCL10* versus a vector control. Sphere numbers per well were normalized to the average number in the control group. Data represent n = 5 independent experiments with quantification of 10 and 12 wells per condition, respectively. Statistics were calculated on biological replicates. **c,** Lung colonization by MDA or SUM breast cancer cells upon ectopic expression of *CXCL9* or *CXCL10* or a vector control, measured using bioluminescence. For panels (b,c), boxes depict median with upper and lower quartiles. Data points show values of biological replicates, whiskers represent minimum and maximum values. *P* values were determined by unpaired one-tailed t-tests.

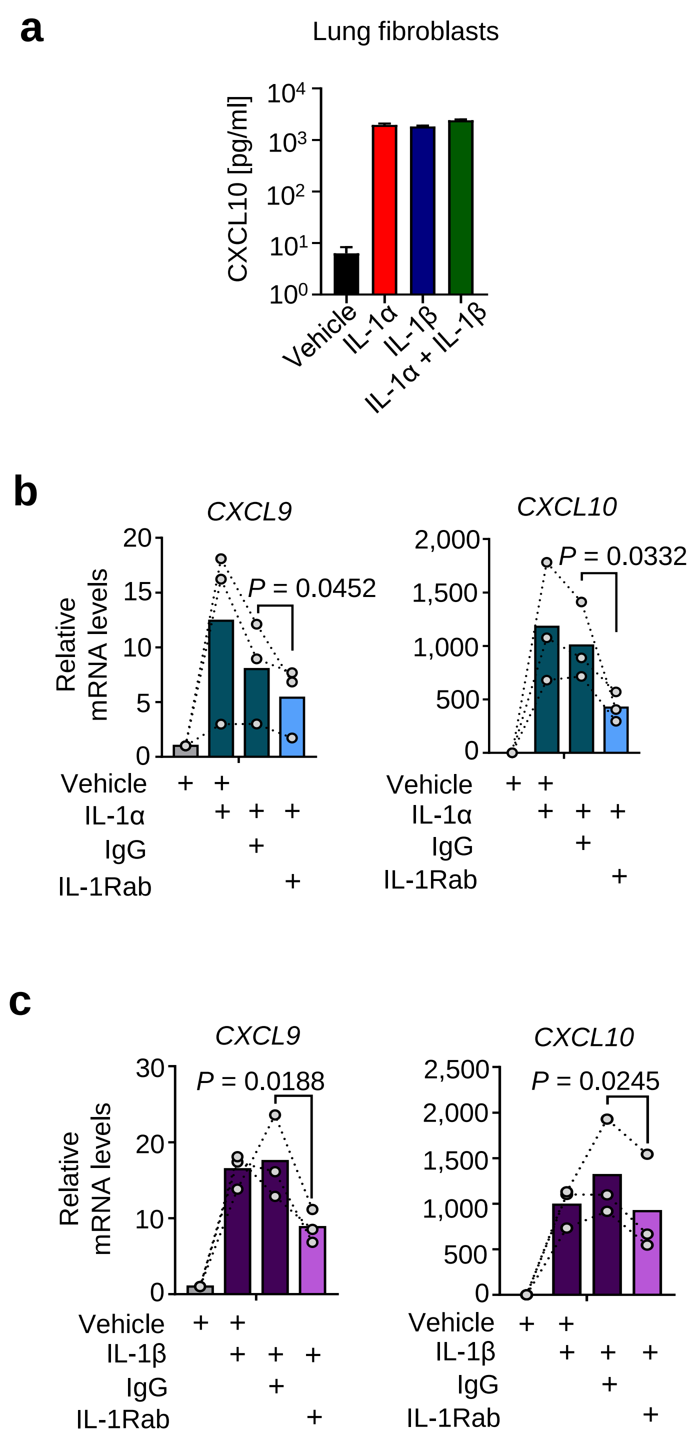
a
Lung fibroblasts
CXCL10 [pg/ml]
10^4
10^3
10^2
10^1
10^0
Vehicle
IL-1α
IL-1β
IL-1α + IL-1β
b
CXCL9
CXCL10
Relative mRNA levels
P = 0.0452
P = 0.0332
Vehicle
IL-1α
IgG
IL-1Rab
c
CXCL9
CXCL10
Relative mRNA levels
P = 0.0188
P = 0.0245
Vehicle
IL-1β
IgG
IL-1Rab

Supplementary Figure 8. CXCL9/10 are induced in lung fibroblasts via IL-1R signaling. **a**, CXCL10 protein levels measured by ELISA in conditioned medium from MRC-5 fibroblasts after stimulation with vehicle control, recombinant human IL-1α (1 ng/ml), IL-1β (1 ng/ml), or IL-1α + IL-1β (1 ng/ml each) for 48 h. Bars depict mean of technical replicates with SD. **b**,**c**, *CXCL9/10* expression in MRC-5 fibroblasts treated with 1 ng/mL recombinant IL-1α (b) or IL-1β (c) in combination with 20 μg/ml IL-1R neutralizing antibody (IL-1Rab) or IgG isotype control (IgG) for 48 h. Linked sets of data points depict relative expression in independent experiments and bars show mean. Expression was analyzed by RT-qPCR; n = 3 biological replicates. *P* values were calculated by ratio-paired one-tailed t-tests

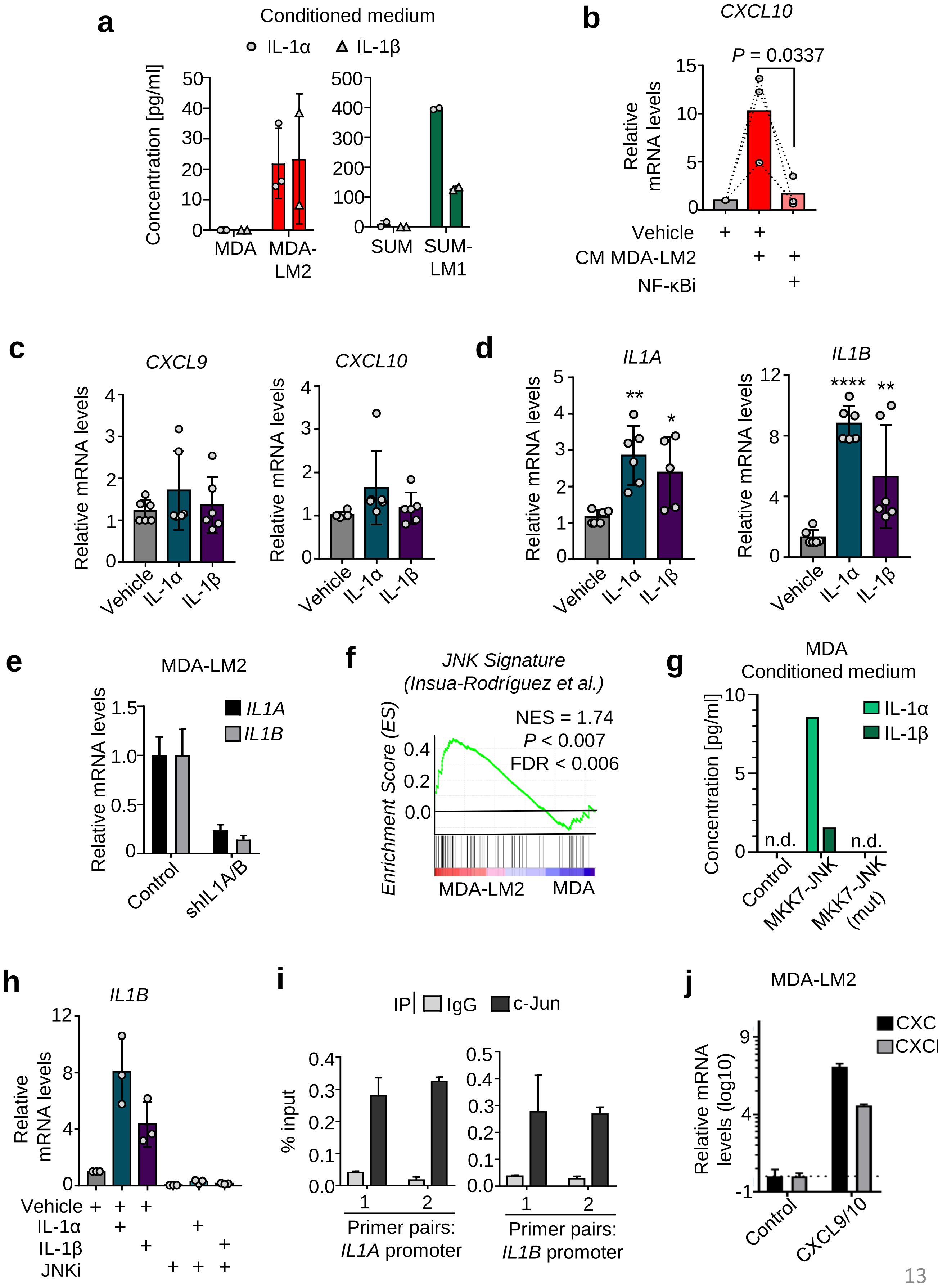
a
Conditioned medium
IL-1α
IL-1β
Concentration [pg/ml]
MDA
MDA-LM2
SUM
SUM-LM1
b
CXCL10
P = 0.0337
Relative mRNA levels
Vehicle
CM MDA-LM2
NF-κBi
c
CXCL9
CXCL10
Relative mRNA levels
Vehicle
IL-1α
IL-1β
d
IL1A
IL1B
e
MDA-LM2
IL1A
IL1B
Control
shIL1A/B
f
JNK Signature
(Insua-Rodríguez et al.)
NES = 1.74
P < 0.007
FDR < 0.006
Enrichment Score (ES)
MDA-LM2
MDA
g
MDA
Conditioned medium
n.d.
Control
MKK7-JNK
MKK7-JNK (mut)
h
IL1B
JNKi
i
IP
IgG
c-Jun
% input
Primer pairs:
IL1A promoter
IL1B promoter
j
CXCL9
CXCL10
Relative mRNA levels (log10)
CXCL9/10

Supplementary Figure 9. IL-1 is induced by JNK signaling in MDA-LM2 and SUM-LM1 breast cancer cells.

a, Protein levels of active human IL-1α and IL-1β as measured in conditioned medium from MDA, MDA-LM2, SUM, and SUM-LM1 cancer cells. Protein levels were measured by ELISA. **b**, *CXCL10* expression in fibroblasts treated with conditioned media (CM) from MDA-LM2 breast cancer cells alone or in combination with 5 μM NF-κB inhibitor (NF-κBi). Linked sets of values show expression in biological replicates. Bars depict mean. *P* value was determined by ratio-paired one-tailed t-test; n = 3 independent experiments. **c**,**d**, Expression levels of *CXCL9* and *CXCL10* (c) or *IL-1A* and *IL-1B* (d) in MDA-LM2 cancer cells after 48 h stimulation with 1 ng/ml recombinant human IL-1α or IL-1β. For panels (a,c and d), data points show values of biological replicates and bars depict mean with SD. **e**, *IL1A* and *IL1B* expression in control and *IL1A/B* double knockdown (shIL1A/B) MDA-LM2 cancer cells. Bars show mean with upper and lower limits. **f**, Enrichment of a JNK response signature[2] in cultured MDA-LM2 versus MDA parental breast cancer cells[3]. NES, normalized enrichment score. *P* value was determined by random permutation test. **g**, Protein levels of active human IL-1α and IL-1β measured in conditioned medium from MDA cancer cells upon ectopic expression of MKK7-JNK, MKK7-JNK(mut), or an empty vector control by ELISA. **h**, Expression levels of *IL1B* in MDA-LM2 cancer cells after 48 h stimulation with 1 ng/ml recombinant human IL-1α or IL-1β combined with vehicle or 5 μM CC-401 (JNKi). Data points represent values of biological replicates. **i**, ChIP-qPCR analysis of c-Jun binding to *IL1A* and *IL1B* promoter chromatin in SUM-LM1 cells. Bars in panels (h,i) depict mean with SD. **j**, Expression of *CXCL9/10* in MDA-LM2 breast cancer cells transduced with *CXCL9*, *CXCL10*, or vector control. Values are mean with upper and lower limits. Gene expression in panels (b-e, h and j) was analyzed by RT-qPCR.

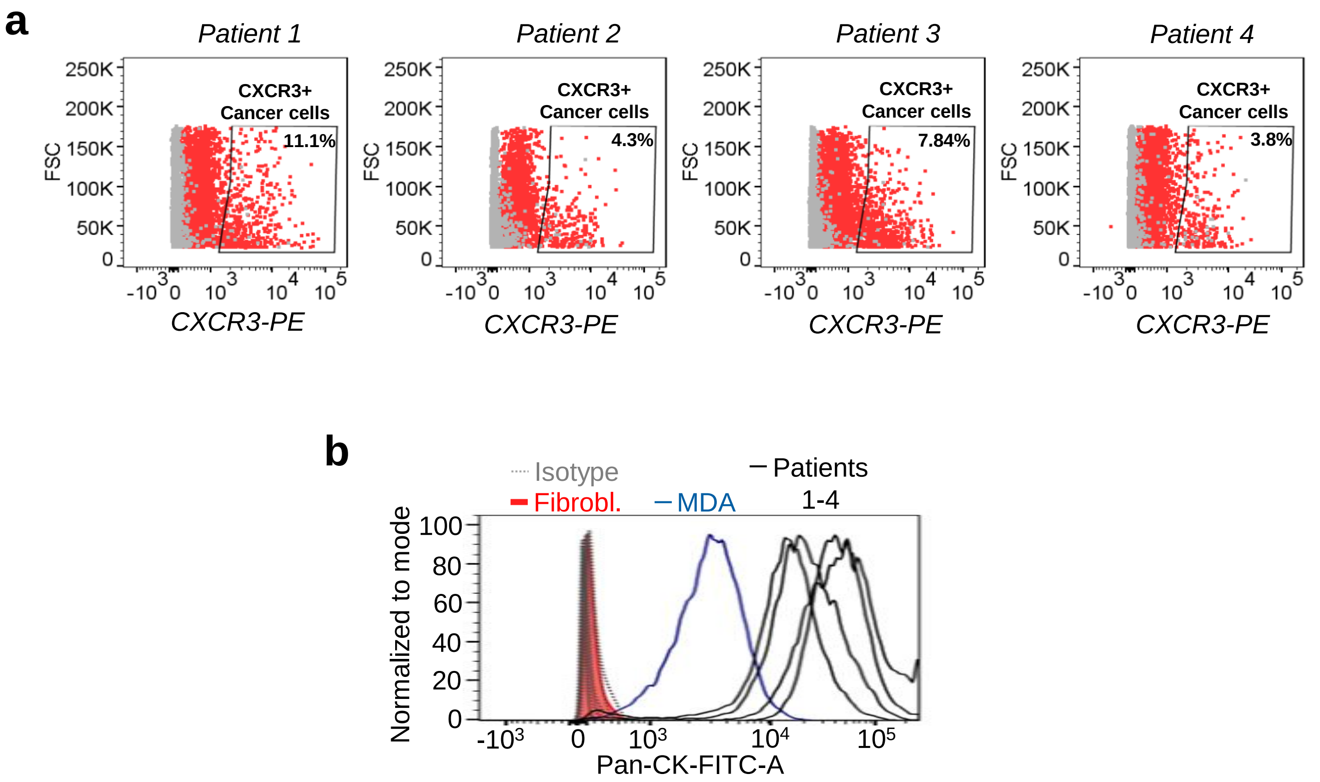

Supplementary Figure 10. CXCR3^{+} breast cancer cells in primary effusion samples from patients with metastasis.

a, CXCR3-positive (CXCR3^{+}) subpopulations in human metastatic cells isolated from pleural effusions (nr. 1 and 4) or ascites (nr. 2 and 3) of breast cancer patients as determined by flow cytometry. CXCR3-PE stained samples in red, isotype control samples in grey. Percentages of CXCR3^{+} cells are shown. **b**, Pan-cytokeratin staining of primary cancer cells derived from pleural effusions or ascites from metastatic breast cancer patients (Patients 1-4; black lines). Isotype controls are indicated in grey (dotted lines). MDA cancer cells were used as positive control (blue line), and MRC-5 fibroblasts were used as negative control (red filled line).

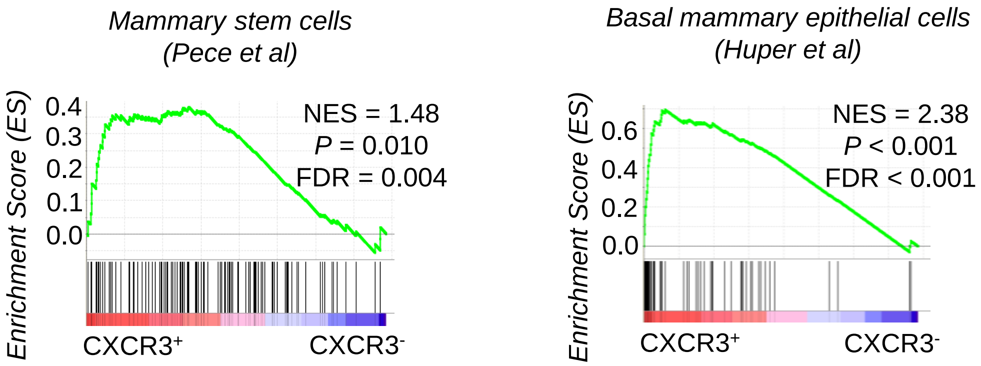

Supplementary Figure 11. Genes expressed in CXCR3^{+} breast cancer cells are associated with stem cell properties.

Enrichment of indicated gene sets[4, 5] in CXCR3^{+} compared to CXCR3^{-} SUM-LM1 breast cancer cells. NES, normalized enrichment score. FDR, false discovery rate. *P* values were determined by random permutation tests.

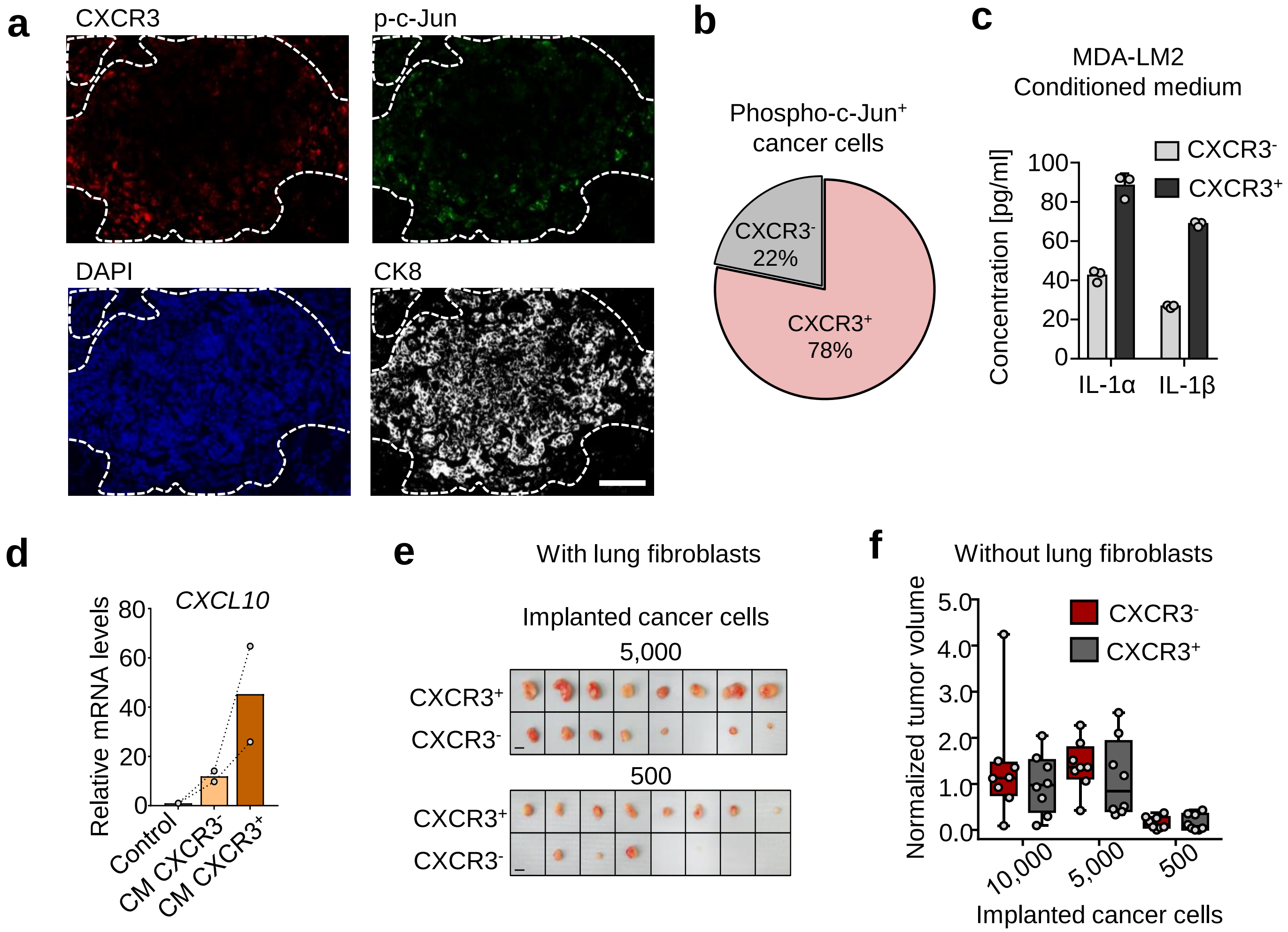

Supplementary Figure 12. CXCR3⁺ breast cancer cells are enriched with activated c-Jun and promote tumor initiation when co-transplanted with fibroblasts.
a, Single channel immunofluorescence analyses of CXCR3, p-c-Jun, DAPI, and cytokeratin 8 (CK8) as shown in Fig. 8e. Dashed lines indicate margin of metastatic nodule as determined by CK8 expression. Scale bar, 100 µm. **b**, Pie chart depicting percentages of p-c-Jun-positive cancer cells as in Fig. 8e that express CXCR3 or not. **c**, Protein levels of secreted human IL-1α and IL-1β measured by ELISA in conditioned medium from isolated $CXCR3^+$ or $CXCR3^-$ MDA-LM2 cancer cells. Data points show values of technical replicates. Bars depict mean with SD. **d**, *CXCL10* expression in MRC-5 human lung fibroblasts treated with control medium or conditioned medium (CM) from $CXCR3^+$ or $CXCR3^-$ 4T1 mammary tumor cells for 48 h. Two independent experiments are shown, bars depict mean from triplicates. **e**, Tumors resected 3 weeks after subcutaneous injection of $CXCR3^+$ or $CXCR3^-$ 4T1 mammary cancer cells and lung fibroblasts in limiting dilutions into either flank of BALB/c mice as quantified in Fig. 8j. Scale bar, 5 mm. **f**, Quantification of tumor sizes 3 weeks after subcutaneous injection of $CXCR3^+$ or $CXCR3^-$ 4T1 cancer cells (without lung fibroblasts) in limiting dilution into either flank of BALB/c mice; *n* = 8 mice per group from two independent experiments. Tumor sizes were normalized to average tumor size established by injection of 10,000 $CXCR3^-$ 4T1 cells. Data points are values of biological replicates. Boxes show median with upper and lower quartiles and whiskers depict maximum and minimum.

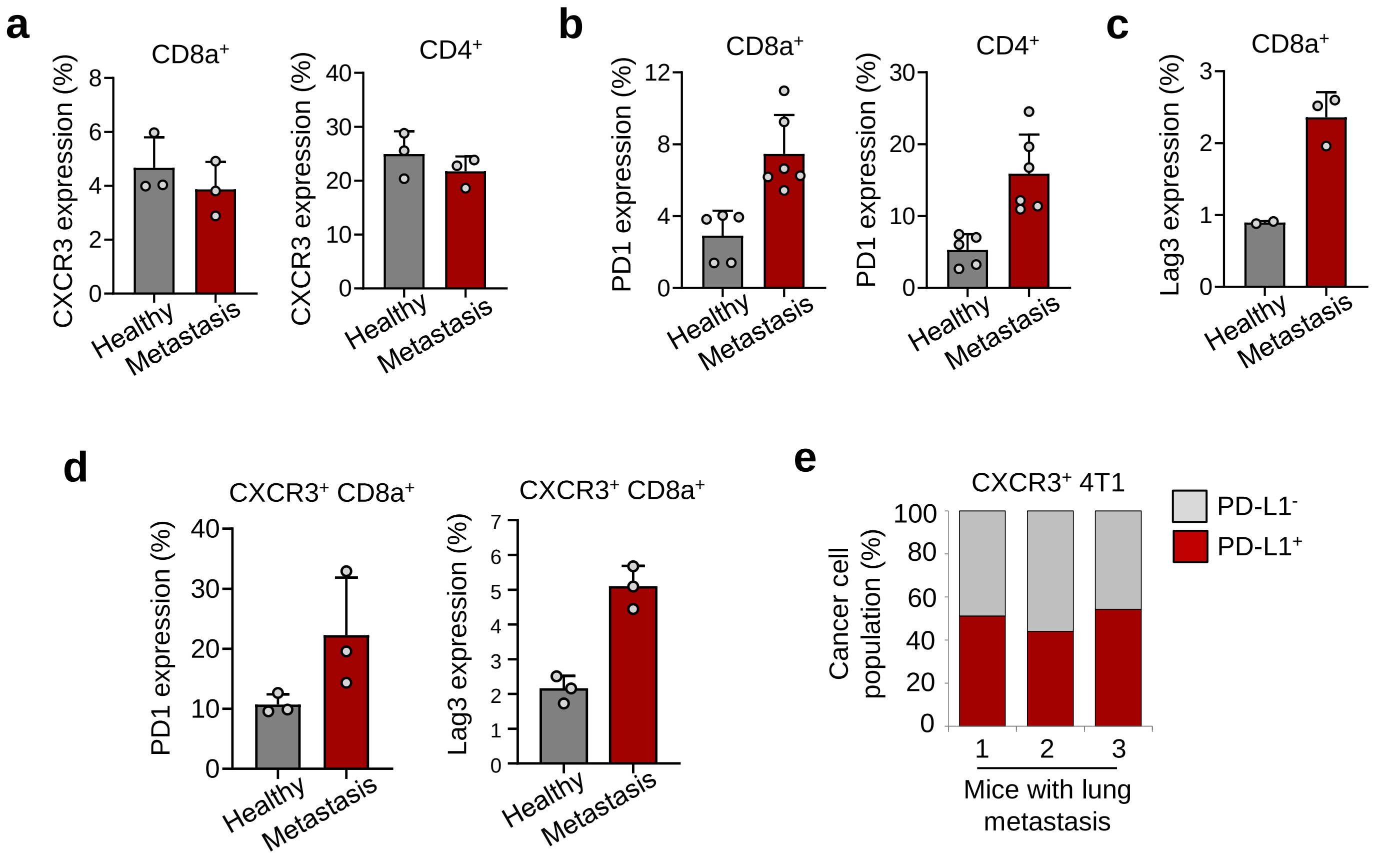

Supplementary Figure 13. Analysis of CXCR3 and checkpoint regulators in T cells from lung metastases in mice.
a-d, Expression of indicated markers measured by flow cytometry in cell populations from healthy lungs or lungs harboring metastases, 2 weeks after intravenous injection of 4T1 cancer cells into BALB/c mice. Data points represent values of biological replicates and bars show mean with SD. **a**, CXCR3-expressing populations within $CD8a^+$ or $CD4^+$ lymphocytes **b**, Expression of immune checkpoint molecule and exhaustion marker PD1 in $CD8a^+$ or $CD4^+$ lymphocytes. **c**, Expression of checkpoint protein and exhaustion marker Lag3 in $CD8a^+$ lymphocytes. **d**, PD1 and Lag3 expression in $CXCR3^+$ $CD8a^+$ lymphocytes. **e**, Expression of PD-L1 in 4T1 cancer cells populations isolated from lungs with growing metastases. Shown are three biological replicates.

a

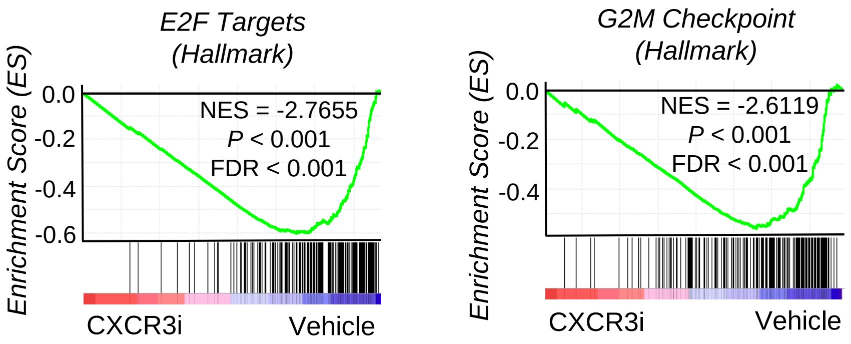

c

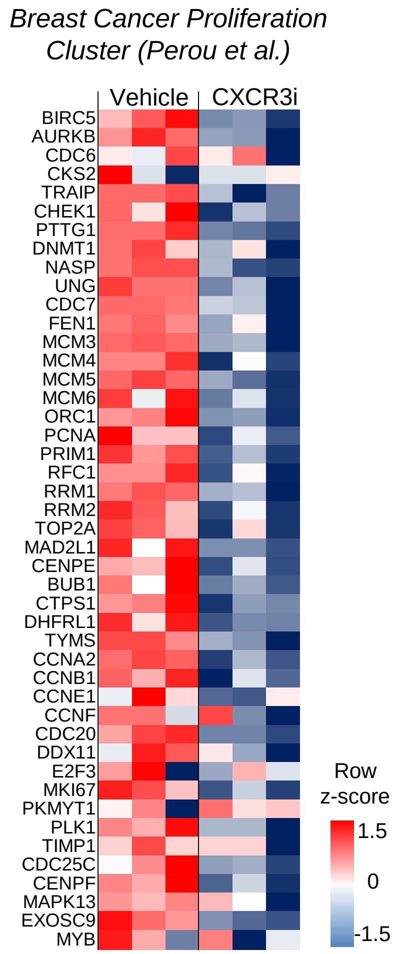

b

GO Term	Fold Enrichment	BH-*P*-value	FDR
Cell cycle process	2.44	0.0001	0.0001
Cell cycle	2.20	0.0002	0.0003
Mitotic cell cycle process	2.73	0.0005	0.0007
Mitotic cell cycle	2.59	0.0007	0.0016
Organelle fission	2.96	0.0027	0.0073
Mitotic nuclear division	3.42	0.0038	0.0121
Nuclear division	2.91	0.0070	0.0264

Supplementary Figure 14. Proliferation gene signatures are repressed in cancer cells after treatment with CXCR3i.

a, Enrichment of indicated gene sets of the MSigDB Hallmark gene set collection in vehicle-treated MDA cancer cells overexpressing *CXCL9* and *CXCL10* compared to treatment with CXCR3i. NES, normalized enrichment score, FDR, false discovery rate. *P* values were determined by random permutation tests. **b**, Gene ontology analysis of top 300 downregulated genes in *CXCL9/10* expressing MDA cancer cells treated with CXCR3i as compared to vehicle control. BH - Benjamini Hochberg, FDR, False Discovery Rate. **c**, Heatmap depicting normalized expression of genes belonging to a Breast Cancer Proliferation Cluster[6] in MDA cancer cells expressing *CXCL9/10* and treated with CXCR3i.

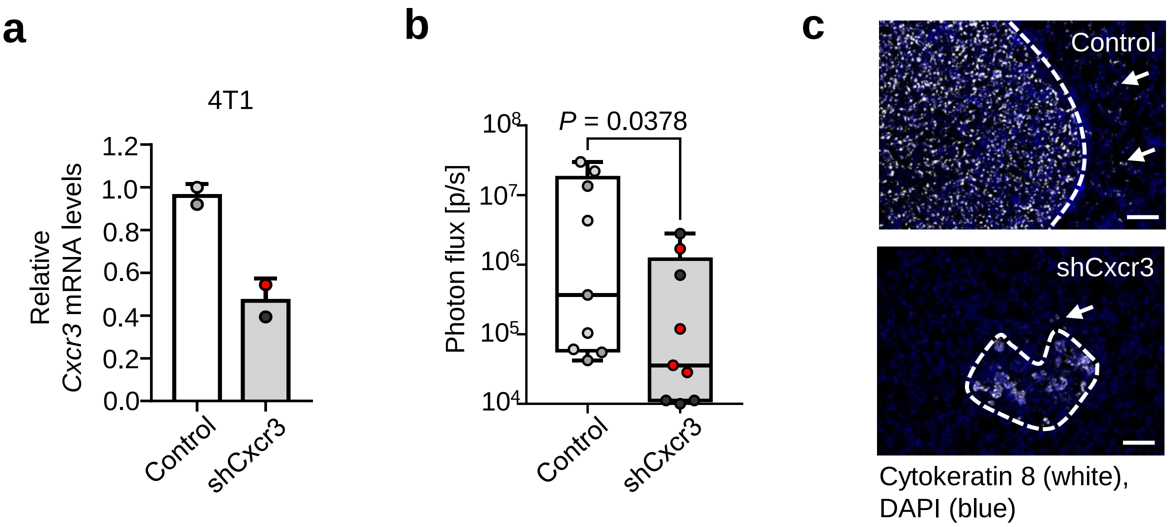

Supplementary Figure 15. Metastatic colonization by CXCR3 knockdown mammary cancer cells.
a, Relative expression of *Cxcr3* in 4T1 mouse mammary tumor cells transduced with shRNAs against *Cxcr3* or a non-targeting shRNAs as control. Two non-targeting shRNAs and two hairpins targeting *Cxcr3* were used. Bars depict mean with SD. **b**, *In vivo* lung bioluminescence in BALB/c mice injected intravenously with shCxcr3 or control 4T1 cancer cells 24 days post injection; n = 9 mice per group. Boxes show median with upper and lower quartiles and whiskers show minimum and maximum values. P value was determined by unpaired one-tailed t-test. For panels (a,b), data points represent values of biological replicates and different hairpins are indicated by different point colors. **c**, Representative immunofluorescence analysis of cytokeratin 8 expression in cancer cells (white) to visualize metastatic nodules in lung sections from (b). Dashed line indicates margin of metastatic foci and arrows point to single cancer cells. DAPI was used for nuclear staining. Scale bar, 100 µm.

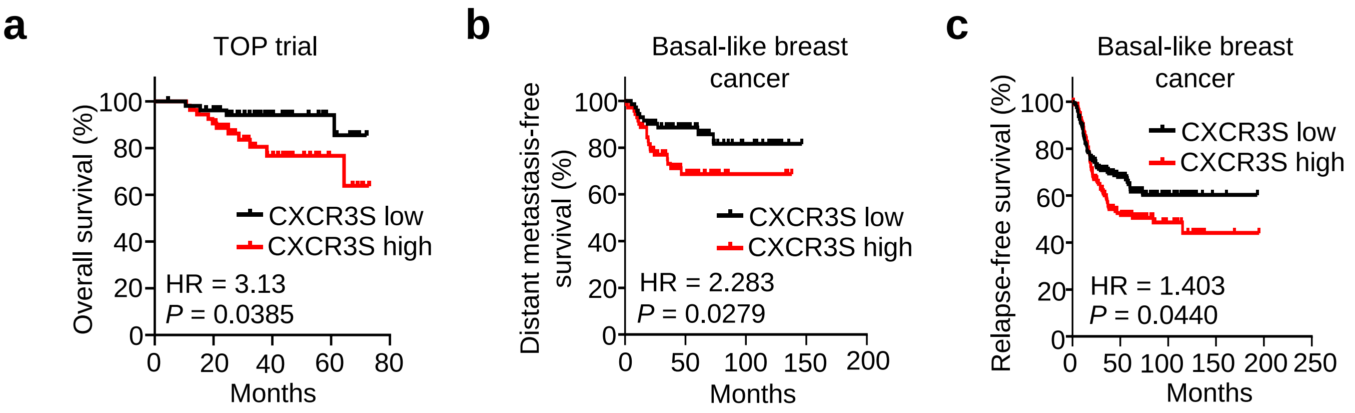

Supplementary Figure 16. CXCR3S expression associates with poor outcome in breast cancer patients.

a-c, Kaplan-Meier analyses of breast cancer patients, associating $CXCR3^+$ cell signature (CXCR3S, mean expression of 65 genes) with overall survival (**a**, TOP trial data set, n = 107 patients), distant metastasis-free survival (**b**, compiled data set from basal-like breast cancer, KM plotter, n = 145) or relapse-free survival (**c**, compiled data set from basal-like breast cancer, KM plotter, n = 360). Median cutoff was used to group patients into CXCR3S low and high. HR, hazard ratio. *P* values were determined by log-rank tests.

Supplementary Table 1. Gene sets used in heatmap for Fig. 1h.

GSEA on gene expression profiles of fibroblasts isolated from lungs of mice with growing MDA231 (MDA) or MDA231-LM2 (MDA-LM2) metastases. Shown are normalized enrichment scores (NES) of fibroblasts isolated at 1 week or 3 weeks post injection. False discovery rate (FDR) < 0.1.

Cellular Responses	NES Micrometastasis		NES Macrometastasis	
	MDA	MDA-LM2	MDA	MDA-LM2
BENPORATH_CYCLING_GENES			2.369	2.252
BENPORATH_PROLIFERATION			2.466	2.336
BIOCARTA_CELLCYCLE_PATHWAY			1.785	1.868
BIOCARTA_G1_PATHWAY			1.903	2.032
BIOCARTA_G2_PATHWAY			1.776	1.736
CHANG_CYCLING_GENES		2.064	2.959	2.903
CHIANG_LIVER_CANCER_SUBCLASS_PROLIFERATION_UP		1.708	2.898	2.749
KAUFFMANN_DNA_REPLICATION_GENES			1.893	1.884
KEGG_CELL_CYCLE			2.204	2.252
KEGG_DNA_REPLICATION		1.880	1.868	2.102
REACTOME_CELL_CYCLE			2.373	2.430
REACTOME_CELL_CYCLE_CHECKPOINTS		1.715	2.226	2.417
REACTOME_CELL_CYCLE_MITOTIC			2.447	2.506
REACTOME_CYCLIN_E_ASSOCIATED_EVENTS_ DURING_G1_S_TRANSITION_		1.921	2.186	2.262
REACTOME_DNA_REPLICATION		1.610	2.391	2.504
REACTOME_DNA_STRAND_ELONGATION		1.889	2.018	2.085
REACTOME_G0_AND_EARLY_G1			1.743	1.668
REACTOME_G1_PHASE			1.866	1.831
REACTOME_G1_S_TRANSITION		1.973	2.373	2.421
REACTOME_LAGGING_STRAND_SYNTHESIS		1.668	1.660	1.748
REACTOME_M_G1_TRANSITION		2.121	2.305	2.402
REACTOME_MITOTIC_G1_G1_S_PHASES		1.834	2.429	2.515
REACTOME_MITOTIC_G2_G2_M_PHASES			1.552	1.615
REACTOME_MITOTIC_M_M_G1_PHASES			2.323	2.430
REACTOME_MITOTIC_PROMETAPHASE			2.184	2.260
REACTOME_REGULATION_OF_MITOTIC_CELL_CYCLE		1.942	2.375	2.451
REACTOME_S_PHASE		2.122	2.399	2.500
REACTOME_SYNTHESIS_OF_DNA		2.186	2.347	2.427
ROSTY_CERVICAL_CANCER_PROLIFERATION_CLUSTER		1.953	3.137	3.106
WHITFIELD_CELL_CYCLE_G1_S			1.694	1.535
WHITFIELD_CELL_CYCLE_G2			2.164	2.050
WHITFIELD_CELL_CYCLE_G2_M			2.407	2.344
WHITFIELD_CELL_CYCLE_LITERATURE		1.624	2.584	2.606
WHITFIELD_CELL_CYCLE_M_G1			1.831	1.516
WHITFIELD_CELL_CYCLE_S			1.647	1.594

ZHANG_PROLIFERATING_VS_QUIESCENT			1.904	1.900
PLASARI_TGFB1_SIGNALING_VIA_NFIC_10HR_UP		1.980	1.429	1.573
PLASARI_TGFB1_SIGNALING_VIA_NFIC_1HR_UP			1.543	1.630
PLASARI_TGFB1_TARGETS_10HR_UP		1.672	2.786	2.662
PLASARI_TGFB1_TARGETS_1HR_UP			2.347	2.112
REACTOME_SIGNALING_BY_TGF_BETA_RECEPTOR_COMPLEX			1.737	1.657
VERRECCHIA_RESPONSE_TO_TGFB1_C1			1.501	
VERRECCHIA_RESPONSE_TO_TGFB1_C5		1.758	1.501	1.742
VERRECCHIA_DELAYED_RESPONSE_TO_TGFB1			1.767	1.814
VERRECCHIA_EARLY_RESPONSE_TO_TGFB1			1.569	1.467
SEKI_INFLAMMATORY_RESPONSE_LPS_UP			2.934	2.690
BIOCARTA_INFLAM_PATHWAY			1.701	1.593
OKUMURA_INFLAMMATORY_RESPONSE_LPS			1.805	1.754
REACTOME_ACTIVATED_TLR4_SIGNALLING			1.472	
WUNDER_INFLAMMATORY_RESPONSE_AND_CHOLESTEROL_UP		1.912	2.496	2.471
KEGG_TOLL_LIKE_RECEPTOR_SIGNALING_PATHWAY			1.875	1.778
ALTEMEIER_RESPONSE_TO_LPS_WITH_MECHANICAL_VENTILATION		1.875	2.914	2.774
REACTOME_TOLL_RECEPTOR_CASCADES			1.619	1.526
REACTOME_TRIF_MEDIATED_TLR3_SIGNALING			1.685	1.649
ZHOU_INFLAMMATORY_RESPONSE_FIMA_UP			1.753	1.535
ZHOU_INFLAMMATORY_RESPONSE_LIVE_UP			2.261	2.030
ZHOU_INFLAMMATORY_RESPONSE_LPS_UP			2.066	1.820
ZHANG_INTERFERON_RESPONSE		2.045	2.242	2.040
REACTOME_INTERFERON_SIGNALING		1.788	2.326	2.316
REACTOME_INTERFERON_GAMMA_SIGNALING		1.603	2.166	2.140
REACTOME_IL1_SIGNALING			1.452	
MAHAJAN_RESPONSE_TO_IL1A_UP		1.642	1.969	1.589
BIOCARTA_IL1R_PATHWAY			1.958	1.693
REACTOME_TRAF6_MEDIATED_NFKB_ACTIVATION		1.698	1.541	1.511
JAIN_NFKB_SIGNALING			1.439	1.444
REACTOME_RIP_MEDIATED_NFKB_ACTIVATION_VIA_DAI		1.736	1.806	1.743
HINATA_NFKB_TARGETS_KERATINOCYTE_UP			2.405	2.239
HINATA_NFKB_TARGETS_FIBROBLAST_UP			2.218	2.182
RASHI_NFKB1_TARGETS			2.336	2.170
MANTOVANI_NFKB_TARGETS_UP			2.084	2.073
REACTOME_TAK1_ACTIVATES_NFKB_BY_PHOSPHORYLATION_ AND_ACTIVATION_OF_IKKS_COMPLEX			1.631	1.518

Supplementary Table 2. Poor outcome gene cluster is enriched in MAFs.

GSEA of poor outcome stromal signature[7] in MDA- or MDA-LM2-associated fibroblasts from mouse lungs harboring micro- or macrometastasis. NES, normalized enrichment score. FDR, false discovery rate. *P* values were determined by random permutation tests.

Comparison	NES	*P* value	FDR
Micrometastasis: MDA-LM2 vs MDA	1.50192	0.01877	0.03859
Macrometastasis: MDA-LM2 vs MDA	1.42618	0.02764	0.10624
MDA-LM2: Macrometastasis vs. Micrometastasis	2.06136	< 0.001	< 0.001
MDA: Macrometastasis vs. Micrometastasis	2.11246	< 0.001	< 0.001

Supplementary Table 3. 65-gene signature in CXCR3⁺ breast cancer cells.

Gene	Linear FC	*P* value	BH-adjusted *P* value
AADAC	2.8154	0.0019	0.0773
ABCA13	2.9828	0.0005	0.0368
AMIGO2	2.4061	0.0024	0.0553
CCDC69	2.3784	0.0020	0.0407
CCL20	2.6882	0.0008	0.0317
CCL5	3.3558	0.0002	0.0317
CD1D	3.3792	0.0005	0.0516
CD33	2.6329	0.0024	0.0773
CD55	2.8481	0.0007	0.0397
CD70	2.5847	0.0013	0.0429
CLCA2	2.9282	0.0012	0.0655
CLDN1	2.7511	0.0014	0.0632
CPA4	2.4566	0.0014	0.0358
CYTIP	2.3729	0.0026	0.0560
DHRS3	2.8679	0.0005	0.0317
EDIL3	2.9759	0.0006	0.0414
EHF	3.0035	0.0009	0.0553
EPHA4	2.6451	0.0011	0.0414
FAM83B	2.8089	0.0010	0.0498
FCAR	2.5491	0.0060	0.1311
FCRLA	2.4967	0.0018	0.0516
FYB	2.8154	0.0006	0.0317
GJB2	2.6635	0.0008	0.0324
GJB5	3.6553	0.0010	0.0827
GPR87	2.5198	0.0019	0.0553
IL1B	2.5257	0.0040	0.0991
IL33	2.8350	0.0011	0.0560
IL6	2.7195	0.0018	0.0719
ITGB4	2.4061	0.0023	0.0544
KRT14	2.6635	0.0010	0.0397
KRT15	2.3894	0.0109	0.1687
KRT17	2.7007	0.0014	0.0553
KRT6A	2.7132	0.0030	0.0973
KRT75	2.3620	0.0060	0.1107
LOC100505946	3.2868	0.0003	0.0324
LOC101927787	2.5315	0.0026	0.0722
LOC201651	2.7132	0.0015	0.0616
LPAR3	2.7195	0.0011	0.0482
MAB21L3	3.8637	0.0002	0.0398
MIR205	2.6268	0.0019	0.0686
MMP10	2.7766	0.0077	0.1687

MMP13	6.0210	0.0000	0.0169
MMP3	3.4422	0.0006	0.0627
MRGPRX3	3.9632	0.0001	0.0317
MT2A	2.5315	0.0028	0.0773
NEURL1B	2.9282	0.0022	0.0951
NTN4	2.7830	0.0009	0.0417
NUAK2	2.5257	0.0058	0.1249
NUP62CL	2.9214	0.0008	0.0498
OASL	3.5554	0.0002	0.0355
P2RY1	2.7511	0.0027	0.0951
PAK6	3.2944	0.0004	0.0401
PHEX	2.3403	0.0140	0.1865
PI3	3.1602	0.0015	0.0859
PKIA	2.4396	0.0028	0.0701
PODXL	2.3729	0.0021	0.0419
PPP1R14C	2.8547	0.0008	0.0417
PRSS3	2.7007	0.0009	0.0407
SDC4	2.4737	0.0022	0.0604
SERPINB2	3.5390	0.0011	0.0835
SERPINB5	2.5315	0.0022	0.0655
SHISA2	2.5491	0.0016	0.0505
SLC35F3	2.6208	0.0020	0.0686
SULF1	2.4967	0.0015	0.0414
TRPV3	3.2565	0.0013	0.0809

Supplementary Table 4. GO term analysis of genes induced in CXCR3^{+} population of SUM-LM1 cancer cells compared to the CXCR3^{-} population.

Category	Term	Fold Enrichment	BH-*P* value	FDR
BP	**GO:0032602~chemokine production**	**22.857**	**0.000**	**0.000**
	**GO:0032722~positive regulation of chemokine production**	**22.271**	**0.002**	**0.012**
	**GO:0032642~regulation of chemokine production**	**19.029**	**0.001**	**0.003**
	**GO:0050729~positive regulation of inflammatory response**	**11.548**	**0.005**	**0.053**
	GO:0042098~T cell proliferation	8.874	0.005	0.054
	GO:0032103~positive regulation of response to external stimulus	7.229	0.002	0.016
	GO:0043410~positive regulation of MAPK cascade	4.776	0.005	0.064
	GO:0030334~regulation of cell migration	4.227	0.002	0.016
	GO:0043408~regulation of MAPK cascade	3.985	0.005	0.069
	GO:2000145~regulation of cell motility	3.934	0.004	0.037
	GO:0040012~regulation of locomotion	3.770	0.005	0.059
	GO:0051270~regulation of cellular component movement	3.610	0.006	0.094
	GO:0016477~cell migration	3.586	0.000	0.001
	GO:0023014~signal transduction by protein phosphorylation	3.521	0.005	0.062
	GO:0051674~localization of cell	3.483	0.000	0.000
	GO:0048870~cell motility	3.483	0.000	0.000
	GO:0040011~locomotion	3.280	0.000	0.000
	GO:0006928~movement of cell or subcellular component	2.903	0.000	0.001
	GO:0051240~positive regulation of multicellular organismal process	2.832	0.005	0.049
	GO:0009888~tissue development	2.748	0.002	0.009
	GO:0032268~regulation of cellular protein metabolic process	2.304	0.005	0.047
	GO:0007166~cell surface receptor signaling pathway	2.242	0.003	0.022
	GO:0044707~single-multicellular organism process	1.754	0.000	0.001
CC	GO:0009986~cell surface	3.833	0.002	0.074
	GO:0005887~integral component of plasma membrane	3.203	0.000	0.000
	GO:0005615~extracellular space	3.191	0.000	0.003
	GO:0031226~intrinsic component of plasma membrane	3.079	0.000	0.001
	GO:0044459~plasma membrane part	2.305	0.001	0.022
	GO:0044421~extracellular region part	2.053	0.000	0.006
	GO:0005576~extracellular region	1.903	0.000	0.008
	GO:0071944~cell periphery	1.754	0.001	0.035
MF	GO:0005102~receptor binding	2.819	0.005	0.040
	GO:0060089~molecular transducer activity	2.687	0.008	0.028
	GO:0004872~receptor activity	2.687	0.008	0.028

Supplementary Table 5. List of primers used in the study; (h) - human, (m) – murine.

Primer	Forward primer sequence (5'- 3')	Reverse primer sequence (5'- 3')
RPL13A (h)	AGATGGCGGAGGTGCAG	GGCCCAGCAGTACCTGTTTA
CXCL9 (h)	GAGTGCAAGGAACCCCAGTAG	GGTGGATAGTCCCTTGGTTGG
CXCL10 (h)	TGGCATTCAAGGAGTACCTCTC	GGACAAAATTGGCTTGCAGGA
IL1A (h)	GCTGAAGGAGATGCCTGAGATA	ACAAGTTTGGATGGGCAACTG
IL1B (h)	AACAGGCTGCTCTGGGATTC	AGTCATCCTCATTGCCACTGT
CXCL9 (h) for cloning of full-length cDNA	ATGAAGAAAAGTGGTGTTCTTTTCCTC	TTATGTAGTCTTCTTTTGACGAGAACGT
CXCL10 (h) for cloning of full-length cDNA	ATGAATCAAACTGCCATTCTGATTTG	TTAAGGAGATCTTTTAGACCTTTCCTTG
CXCL9 (h) for comparing to levels of murine Cxcl9	CATCAGCACCAACCAAGGGA	AGGGCTTGGGGCAAATTGTT
CXCL10 (h) for comparing to levels of murine Cxcl10	TGCCATTCTGATTTGCTGCC	TGCAGGTACAGCGTACAGTT
IL1A (h) Primer pair 1 for ChIP-qPCR	GGCTGTAGCTTTAGAGAAGGCA	GGCGTTTGAGTCAGCAAAGG
IL1A (h) Primer pair 2 for ChIP-qPCR	CCTTTGCTGACTCAAACGCC	AGCCACGCCTACTTAAGACAA
IL1B (h) Primer pair 1 for ChIP-qPCR	CCTTGTGCCTCGAAGAGGTT	TCTCAGCCTCCTACTTCTGCT
IL1B (h) Primer pair 2 for ChIP-qPCR	ATGGGTACAATGAAGGGCCAA	GCTCCTGAGGCAGAGAACAG
B2m (m)	CCTGGTCTTTCTGGTGCTTG	CCGTTCTTCAGCATTTGGAT
Cxcl9 (m)	TCGGACTTCACTCCAACACAG	AGGGTTCCTCGAACTCCACAC
Cxcl10 (m)	GAGAGACATCCCGAGCCAAC	GGGATCCCTTGAGTCCCAC
Il1a (m)	CGCTTGAGTCGGCAAAGAAAT	TGGCAGAACTGTAGTCTTCGT
Il1b (m)	TGCCACCTTTTGACAGTGATG	ATGTGCTGCTGCGAGATTTG
Cxcr3 (m)	CCAGCCAAGCCATGTACCTT	TCGTAGGGAGAGGTGCTGTT
Pdgfra (m)	AACCTGAACCCAGACCATCG	CGGAGGAGAACAAAGACCGC
Pdgfrb (m)	GTGGAGATTCGCAGGAGGTCA	TCGGATCTCATAGCGTGGCTTC
Acta2 (m)	AGAGGCACCACTGAACCCTA	CCAGCACAATACCAGTTGTACG
Pecam1 (m)	AGTGGAAGTGTCCTCCCTTG	GCCTTCCGTTCTTAGGGTC
Cdh5 (m)	GGGCAAGCTGGTAGTACAGA	ACTGCCCATACTTGACCGTG
Ptprc (m)	TGGCCTTTGGATTTGCCCTT	CTGTTGTGCTCAGTTCATCACT
Cd14 (m)	GACCATGGAGCGTGTGCTTG	CTGGACCAATCTGGCTTCGG
Cdh1 (m)	CCTGCCAATCCTGATGAAAT	GAACCACTGCCCTCGTAATC
Epcam (m)	TCATCGCGGGGATTGTTGTC	TGTGGATCTCACCCATCTCCT
Cxcl9 (m) for cloning of full-length cDNA	ATGAAGTCCGCTGTTCTTTTCCT	TTATGTAGTCTTCCTTGAACGACGAC
Cxcl10 (m) for cloning of full-length cDNA	ATGAACCCAAGTGCTGCCG	TTAAGGAGCCCTTTTAGACCTTTTTTGG C
Cxcl9 (m) for comparing to levels of human CXCL9	CTGGAGCAGTGTGGAGTTCG	AGGCAGGTTTGATCTCCGTTC
Cxcl10 (m) for comparing to levels of human CXCL10	ATGACGGGCCAGTGAGAATG	TCAACACGTGGGCAGGATAG
miRE-Xho-fw	TGAACTCGAGAAGGTATATTGCTGTTG ACAGTGAGCG	
miRE-EcoOligo-rev		TCTCGAATTCTAGCCCCTTGAAGTCCGA GGCAGTAGGC
BstBI-MKK7Jnk1a1-Fw	CATCTTCGAACGGCAGCCAACATGGA C	
XmaI-MKK7Jnk1a1-Rv		CATCCCCGGGGAGCTCGAGTCACTGCT GC

Supplementary methods

Gel contraction assay

Number of lung fibroblasts, isolated from NSG mice, was adjusted to 1.5×10^5 cells/ml in PBS. Then, 800 µl of the fibroblast suspension were mixed with 400 µl of a 3 mg/ml bovine collagen solution (Advanced BioMatrix). This was followed by 8 µl of 1 M NaOH added to the cell-collagen mixture and the solution was mixed by pipetting. 1 ml of the mixture was immediately transferred to a 12-well plate and gels were allowed to solidify at room temperature for 20 min. Cancer cell CM obtained from 250,000 MDA or MDA-LM2 cancer cells seeded in 2 ml MEMα medium per 6-well for 48 h was filtered through 0.45 µm filter and 1 ml CM or MEMα control medium was added per well. Gels were dissociated from the wells by gently running a pipet tip along gel edges and swirling the plate. 12-well plates were incubated at 37 °C and gel diameters were recorded after 24 h.

Supplementary references

1. Naba, A. *et al*. The matrisome: in silico definition and in vivo characterization by proteomics of normal and tumor extracellular matrices. *Molecular & Cellular Proteomics : MCP* **11**, M111 014647 (2012).

2. Insua-Rodriguez, J. *et al*. Stress signaling in breast cancer cells induces matrix components that promote chemoresistant metastasis. *EMBO Molecular Medicine* **10** (2018).

3. Minn, A.J. *et al*. Genes that mediate breast cancer metastasis to lung. *Nature* **436**, 518-524 (2005).

4. Pece, S. *et al*. Biological and molecular heterogeneity of breast cancers correlates with their cancer stem cell content. *Cell* **140**, 62-73 (2010).

5. Huper, G. & Marks, J.R. Isogenic normal basal and luminal mammary epithelial isolated by a novel method show a differential response to ionizing radiation. *Cancer Research* **67**, 2990-3001 (2007).

6. Perou, C.M. *et al*. Molecular portraits of human breast tumours. *Nature* **406**, 747-752 (2000).

7. Finak, G. *et al*. Stromal gene expression predicts clinical outcome in breast cancer. *Nature Medicine* **14**, 518-527 (2008).
