# Supplementary material for: Metastasis-initiating cells induce and exploit a fibroblast niche to fuel malignant colonization of the lungs
Source: Nat Commun. 2020 Mar 20;11:1494. doi: 10.1038/s41467-020-15188-x (PMC7083860; doi:10.1038/s41467-020-15188-x)
Supplement: Supplementary file 2 — Reporting Summary [file 41467_2020_15188_MOESM2_ESM.pdf]

nature research

Corresponding author(s): Thordur Oskarsson

Last updated by author(s): Jan 19, 2020

Reporting Summary

Nature Research wishes to improve the reproducibility of the work that we publish. This form provides structure for consistency and transparency in reporting. For further information on Nature Research policies, see Authors & Referees and the Editorial Policy Checklist .

Statistics

For all statistical analyses, confirm that the following items are present in the figure legend, table legend, main text, or Methods section.

n/a	Confirmed	
☐	☒	The exact sample size (n) for each experimental group/condition, given as a discrete number and unit of measurement
☐	☒	A statement on whether measurements were taken from distinct samples or whether the same sample was measured repeatedly
☐	☒	The statistical test(s) used AND whether they are one- or two-sided<br>*Only common tests should be described solely by name; describe more complex techniques in the Methods section.*
☒	☐	A description of all covariates tested
☐	☒	A description of any assumptions or corrections, such as tests of normality and adjustment for multiple comparisons
☐	☒	A full description of the statistical parameters including central tendency (e.g. means) or other basic estimates (e.g. regression coefficient) AND variation (e.g. standard deviation) or associated estimates of uncertainty (e.g. confidence intervals)
☐	☒	For null hypothesis testing, the test statistic (e.g. F, t, r) with confidence intervals, effect sizes, degrees of freedom and P value noted<br>*Give P values as exact values whenever suitable.*
☒	☐	For Bayesian analysis, information on the choice of priors and Markov chain Monte Carlo settings
☒	☐	For hierarchical and complex designs, identification of the appropriate level for tests and full reporting of outcomes
☐	☒	Estimates of effect sizes (e.g. Cohen's d, Pearson's r), indicating how they were calculated

Our web collection on statistics for biologists contains articles on many of the points above.

Software and code

Policy information about availability of computer code

Data collection	- ImageScope (version 11.2.0.780)<br>- Living Image (version 4.4)<br>- Quant Studio Real-Time PCR Software v1.3 (appliedbiosystems)<br>- BD FACSDiva Software version 8.0.1 (acquisition)
Data analysis	- GraphPad Prism 7 (version 7.02)<br>- Chipster (version 3.8.1)<br>- GSEA Software (version 2.2.0)<br>- Database for Annotation, Visualization and Integrated Discovery (DAVID) version 6.8<br>- RStudio Version 1.0.143<br>- ZEN 2 (blue edition)<br>- Fiji extension of the ImageJ software (version 1.51d)<br>- ImageScope (version 11.2.0.780)<br>- Living Image (version 4.4)<br>- Quant Studio Real-Time PCR Software v1.2<br>- FlowJo 10.4.1 for Windows 7, Java Version: 1.8.0

For manuscripts utilizing custom algorithms or software that are central to the research but not yet described in published literature, software must be made available to editors/reviewers. We strongly encourage code deposition in a community repository (e.g. GitHub). See the Nature Research guidelines for submitting code & software for further information.

Data

Policy information about availability of data

All manuscripts must include a data availability statement. This statement should provide the following information, where applicable:
- Accession codes, unique identifiers, or web links for publicly available datasets
- A list of figures that have associated raw data
- A description of any restrictions on data availability

The datasets generated during and/or analyzed during the current study are available in the GEO repository, https://www.ncbi.nlm.nih.gov/geo/browse/, under the Accession names: GSE121947 (datasets generated in this study), GSE16446, GSE14020 (datasets analyzed in this study).

Field-specific reporting

Please select the one below that is the best fit for your research. If you are not sure, read the appropriate sections before making your selection.

☒ Life sciences ☐ Behavioural & social sciences ☐ Ecological, evolutionary & environmental sciences

For a reference copy of the document with all sections, see nature.com/documents/nr-reporting-summary-flat.pdf

Life sciences study design

All studies must disclose on these points even when the disclosure is negative.

Sample size	Sample sizes were generally determined based on previous studies involving similar experimental setup.<br>For animal experiments, the number of mice per group was determined by a statistician. As target parameters the bioluminescence of the lung at the end of the experiment was used to compare the control group with the experimental group from previous experiments. As type I error we used 5 %, as type II error 20 % and for the standard deviation 0.3 log bioluminescence units. The simulation was done with the software R, version 3.2.0.
Data exclusions	Data exclusions were not needed.
Replication	Experimental results were reproduced. Repeats are indicated in Figure Legends and Methods.
Randomization	In vivo experiments were performed using inbred NOD Scid gamma (NSG) mice, wildtype or Il1r1tm1Roml C57BL/6 mice (IL-1RI-) mice, or commercially available BALB/c mice (bought from Janvier Labs or Envigo). Mice were randomized from different cages (with similar age) for control- and treatment groups before injection / treatment.
Blinding	Blinding was used when immunostained histology samples were analyzed. The investigator was not aware of the sample genotype, -treatment or clinical association when analyzing immunostaining.<br><br>Mouse bioluminescence imaging /analysis of bioluminescent signals was performed without any knowledge of experimental treatment or mouse/cancer cell genotype.<br><br>Sphere quantification was done in a blinded manner by four different scientists obtaining identical results.

Reporting for specific materials, systems and methods

We require information from authors about some types of materials, experimental systems and methods used in many studies. Here, indicate whether each material, system or method listed is relevant to your study. If you are not sure if a list item applies to your research, read the appropriate section before selecting a response.

Materials & experimental systems

n/a	Involved in the study
☐	☒ Antibodies
☐	☒ Eukaryotic cell lines
☒	☐ Palaeontology
☐	☒ Animals and other organisms
☐	☒ Human research participants
☒	☐ Clinical data

Methods

n/a	Involved in the study
☒	☐ ChIP-seq
☐	☒ Flow cytometry
☒	☐ MRI-based neuroimaging

Antibodies

Antibodies used	Flow Cytometry:

- APC anti-mouse CD140a-APC, eBioscience, Cat. 17-1401-81, Clone APA5, Lot 4303763
- APC anti-mouse CD140b-APC, eBioscience, Cat. 17-1402-82, Clone APB5, Lot 4303343
- APC rat IgG2a k IsoControl, eBioscience, Cat. 17-4321-81, Clone eBR2a, Lot 4299699
- PE anti-mouse CD45-PE, eBiosciences, Cat. 12-0451-82, Clone 30-F11, Lot E013632
- PE anti-mouse CD11b, BD Biosciences, Cat. 553311, Clone M1/70, Lot 04806
- PE anti-mouse CD31-PE, eBioscience, Cat. 12-0311-83, Clone 390, Lot 4311526
- PE anti-mouse CD326 (EpCAM), eBioscience, Cat. 12-5791-83, Clone G8.8, Lot E01779-1631
- PE anti-human CD183 (CXCR3) Antibody, BioLegend, Cat 353706, Clone G025H7, Lot B236934
- PE mouse IgG1, κ Isotype Ctrl (FC) Antibody, BioLegend, Cat 400114, Clone MOPC-21 , Lot B245983
- PE anti-mouse CD183 (CXCR3) Antibody, BioLegend, Cat. 126506, Clone ,CXCR3-173 Lot B240523
- PE Armenian Hamster IgG Isotype Ctrl Antibody, BioLegend, Cat. 400908, Clone HTK888, Lot B243156
- FITC Anti-Cytokeratin, Miltenyi, Cat. 130-080-101, Clone CK3-6H5, Lot 5190722032
- FITC mouse IgG1 isotype, Miltenyi, Cat. 130-113-199, Clone IS5-21F5
- APC anti-mouse CD4 Antibody, BioLegend, Cat. 100411, Clone GK1.5, Lot B237268
- PE/Cy5 anti-mouse CD8a Monoclonal Antibody, BioLegend, Cat. 100709, Clone 53-6.7
- PE/Cy7 anti-mouse CD223 (LAG-3) Antibody, BioLegend, Cat. 125226, Clone C9B7W, Lot B249815
- Brilliant Violet 421™ anti-mouse CD279 (PD-1) Antibody, BioLegend, Cat. 135217, Clone 29F.1A12, Lot B256184
- APC anti-mouse CD274 (B7-H1, PD-L1) Antibody, BioLegend, Cat. 124311, Clone 10F.9G2

Immunohistochemistry:
- Novocastra™ Vimentin Antibody, clone SRL-33, product code: NCL-L-VIM-572, Leica Biosystems
- Anti-alpha smooth muscle Actin antibody, abcam, Cat. ab7817, Clone 1A4

Immunofluorescence:
- Anti-mouse/rat Ki-67, eBioscience, Cat. 14-5698-80, Clone SolA15, Lot 4316838
- Anti-alpha smooth muscle Actin antibody, abcam, Cat. ab7817, Clone 1A4
- Anti-Cytokeratin 8 antibody, abcam, Cat. ab53280, Clone EP1628Y
- Anti-Ki-67 Monoclonal Antibody, eBioscience, Cat. 14-5698-82, Clone SolA15, Lot 4304318
- anti-mouse CD183 (CXCR3), BioLegend, Cat. 126502, Clone CXCR3-173, Lot B256288
- Phospho-c-Jun (Ser73) (D47G9) XP® Rabbit mAb, Cell Signaling, Cat. 3270S, Lot 5

Chromatin-IP:
- c-Jun, Cat. 9165, Clone 60A8, Cell Signaling Technology (validated by the company). The use of this antibody for ChIP assays has been previously reported (Zhang et al., Nature Structural & Molecular Biology 2016).
- Isotype IgG, cat. No 2729, Cell Signaling Technology

Neutralizing Antibodies:
- Anti-hIL1-RI neutralizing antibody, R&D, Cat. AB-269-NA, Lot MU0312071
- Normal goat IgG control, R&D, at. AB-108-C, Lot ES4116101

Validation

Primary antibodies used in this study have been validated by the respective companies for the respective species and application. We used indicated isotype controls as negative controls.

Eukaryotic cell lines

Policy information about cell lines

Cell line source(s)

MDA-MB-231, 4T1 and MRC-5 cells were obtained from ATCC. MDA231-LM2 was generated by the Massague group by in vivo selection using the breast cancer cell line MDA-MB-231 (Minn et al., Nature 2005). E0771 cells were obtained from CH3 BioSystems. SUM159 cell line was obtained from Asterand Bioscience. SUM159-LM1 was generated by the Oskarsson lab by in vivo selection using the breast cancer cell line SUM159 (Insua-Rodríguez et al, 2018).

Authentication

Cell lines were authenticated using Multiplex Cell Authentication (MCA) by Multiplexion (Heidelberg, Germany). Cell line Identification was done using Single Nucleotide Polymorphism (SNP)-profiling and the complete genotype information is compared to a reference database of currently 850 distinct reference cell lines authenticated Short tandem repeat (STR) Profiling. The SNP profiles matched known profiles (cell lines) or were unique (patient samples).

Mycoplasma contamination

The purity of cell lines was validated using the Multiplex cell Contamination Test (McCT) by Multiplexion (Heidelberg, Germany). Contaminations are detected with specific primer sequences in a multiplex PCR targeting cellular, bacterial and viral genome regions followed by hybridization using specific oligonucleotide probes. No Mycoplasma, SMRV or interspecies contamination was detected.

Commonly misidentified lines
(See ICLAC register)

No cell lines listed in the ICLAC database were used.

Animals and other organisms

Policy information about studies involving animals; ARRIVE guidelines recommended for reporting animal research

Laboratory animals	NOD Scid gamma (NSG) mice from in-house breeding, BALB/c mice (Janvier Labs or Envigo), and Il1r1tm1Roml (IL-1RI-) or wildtype C57BL/6 mice (Jackson Laboratory) mice were used for experiments. Only female mice were used for the study and mice were 6-8 weeks of age.
Wild animals	The study did not involve wild animals.
Field-collected samples	The study did not involve samples collected from the field.
Ethics oversight	Animal care and procedures were approved by the governmental review board of the state of Baden-Wuerttemberg, Regierungspraesidium Karlsruhe, under the authorization numbers G-51/13, G-81/16, G-289/16, G-218/16, DKFZ-299 and DKFZ-356 and followed the German legal regulations.

Note that full information on the approval of the study protocol must also be provided in the manuscript.

Human research participants

Policy information about studies involving human research participants

Population characteristics	The study involves human material derived from two different sources: archived lung metastasis tissue sections and patient-derived pleural effusions or ascites.<br><br>Archived samples were acquired from the National Center for Tumor Diseases (NCT) in Heidelberg. The samples correspond to tissue sections of lung metastasis lesions from 12 female breast cancer patients.<br><br>Pleural effusion and ascites were collected from female, late-stage breast cancer patients undergoing treatment for metastatic breast cancer at the University clinics in Heidelberg or Mannheim. Exudative fluids were obtained as part of patient care and would be otherwise discarded. Patients signed a consent stating that isolated samples can be used for research purposes.<br><br>After collection by medical personnel, fluid samples were directly sent to the laboratory and processed for cancer cell isolation as described in the materials and methods section of the manuscript. No sensitive or personal data were collected. The usage of these samples was approved by the ethical committees in Heidelberg and Mannheim (Ethikkommision Medizinische Fakultät). All biological samples from patients were collected in compliance with EU legislation.
Recruitment	Pleural effusion and ascites were collected from female, late-stage breast cancer patients undergoing treatment for metastatic breast cancer at the University clinics in Heidelberg or Mannheim. These exudative fluids are removed as an important part of patient care, in order to relieve pain, pressure in the lungs and abdomen and facilitate breathing.
Ethics oversight	Collection of archived tissue samples has been approved by the ethical committee at the Faculty Clinic (Ethikkommision Medizinische Fakultät Heidelberg).<br><br>The usage of these samples was approved by the ethical committees in Heidelberg and Mannheim (Ethikkommision Medizinische Fakultät). All biological samples from patients were collected in compliance with EU legislation.

Note that full information on the approval of the study protocol must also be provided in the manuscript.

Flow Cytometry

Plots

Confirm that:

- [x] The axis labels state the marker and fluorochrome used (e.g. CD4-FITC).
- [x] The axis scales are clearly visible. Include numbers along axes only for bottom left plot of group (a 'group' is an analysis of identical markers).
- [x] All plots are contour plots with outliers or pseudocolor plots.
- [x] A numerical value for number of cells or percentage (with statistics) is provided.

Methodology

Sample preparation	For flow cytometric analysis and sorting of cells obtained from mouse lungs, lungs were digested at 37 °C in PBS supplemented with 0.5 % Collagenase, 1 % Dispase and 30 ug/ml DNase for 30-45 min. Single cell suspensions were obtained by filtering through 70 um nylon filters and red blood cells were lysed with ACK lysis buffer. Cells were then counted on a ViCell Automated Cell Counter to ensure use of sufficient amounts of antibodies. Mouse FcR Blocking Reagent was used to block unwanted binding

of antibodies to mouse cells expressing Fc receptors for 10 min on ice, after which antibody cocktails were added to the desired final concentration. Staining was done for 30 min on ice in the dark. Afterwards, cells were washed three times, resuspended in FACS buffer contaning DAPI, and analyzed on BD LSR Fortessa or FACSAria1 machines.

For flow cytometric staining of cultured cells, cells were detached from culture flasks using trypsin, or dissociated with accutase, when grown under non-adherent conditions. Cells were counted on a ViCell Automated Cell Counter to ensure use of sufficient amounts of antibodies. Staining was done for 30 min on ice in the dark. Afterwards, cells were washed three times, resuspended in FACS buffer contaning DAPI, and analyzed on BD LSR Fortessa or FACSAria1 machines.

Instrument	LSR Fortessa and FACSAria1 (BD)
Software	Data collection:<br>BD FACSDiva Software version 8.0.1<br>Data Analysis:<br>FlowJo 10.4.1 for Windows 7
Cell population abundance	Sorting purities were evaluated by acquisition of post-sort fractions directly after the sort using the same gates as for sorting. Purities were generally above 90 %.
Gating strategy	Gating strategies for fibroblast isolation is shown in Supplementary Fig. 1b-d. Briefly, cells were gated on FSC-A/SSC-A, doublets were excuded by FSC-H/FSC-A and dead cells were excluded by DAPI stain. Gating for DAPI was determined using an unstained control. Cancer cell exclusion was done by gating on GFP-negative cell fraction, and the gate boundary was determined by acquisition of a healthy lung sample that does not contain GFP-positive cancer cells. Fibroblasts were gated on the PE-negative, APC-positive fraction, where PE stain was a panel of negative selection markers of which individual antibody concentrations had been adjusted to similar fluorescence intensities during acqusition, so that PE-negative fractions were indeed negative for all PE-conjugated antibodies used for negative selection. Gates for APC-positive fractions were set based on APC-unstained controls and confirmed with APC-conjugated isotype controls to ensure specific staining.<br><br>Gating strategy for CXCR3+/- cancer cells included gating on cell populations based on FSC-A/SSC-A, doublets exclusion by FSC-H/FSC-A and dead cell exclusion using DAPI stain. Isotype controls were used to set gates for CXCR3-positive and CXCR3-negative fractions.

☒ Tick this box to confirm that a figure exemplifying the gating strategy is provided in the Supplementary Information.
